# Supplementary material for: 3,4‐Dimethoxychalcone induces autophagy through activation of the transcription factors TFE3 and TFEB
Source: EMBO Mol Med. 2019 Oct 14;11(11):e10469. doi: 10.15252/emmm.201910469 (PMC6835206; doi:10.15252/emmm.201910469)
Supplement: Supplementary file 1 — Appendix [file EMMM-11-e10469-s001.pdf]

Appendix

3,4-Dimethoxychalcone induces autophagy through activation of the transcription factors TFE3 and TFEB

Guo Chen, Wei Xie, Jihoon Nah, Allan Sauvat, Peng Liu, Federico Pietrocola, Valentina Sica, Didac Carmona-Gutierrez, Andreas Zimmermann, Tobias Pendl, Jelena Tadic, Martina Bergmann, Sebastian J. Hofer, Lana Domuz, Sylvie Lachkar, Maria Markaki, Nektarios Tavernarakis, Junichi Sadoshima, Frank Madeo, Oliver Kepp* & Guido Kroemer*

Table of content

- Appendix Fig S1 3,4-DC does not cause traits of cell death.
- Appendix Fig S2 3,4-DC induces the turnover of autophagic cargo.
- Appendix Fig S3 3,4-DC induces transcription-dependent p62 and LC3 expression.
- Appendix Fig S4 3,4-DC increases LAMP1 expression.
- Appendix Fig S5 3,4-DC increases lysosomal biogenesis.
- Appendix Fig S6 3,4-DC and 4,4'-DC induce distinct phenotypes.
- Appendix Fig S7 3,4-DC is non-toxic and well tolerated in vivo.
- Appendix Table S1 List of agents used for HTS screen.
- Appendix Table S2 Abbreviations of the chalcones analyzed in this study.
- Appendix Table S3 List of utilized RT-PCR primers.
- Appendix Table S4 Statistics.
- Appendix Table S5 Statistics for tumor growth curves.
- Appendix Table S6 Statistics for in vivo experimentation.

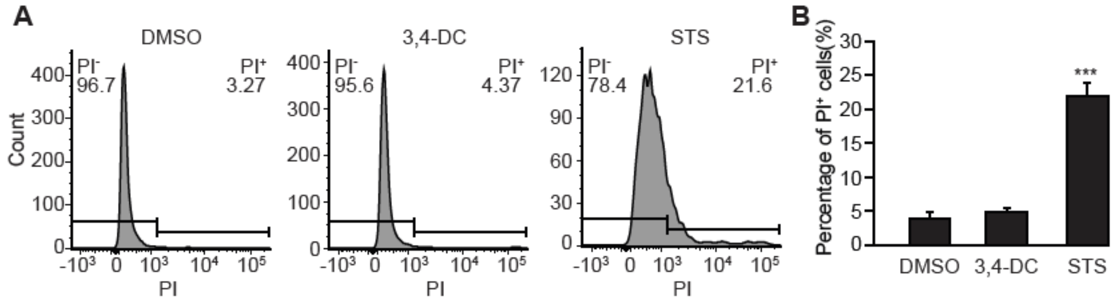

Appendix Figure S1

Appendix Fig S1 3,4-DC does not cause traits of cell death **(A,B)** U2OS cells treated with 30 µM 3,4-DC or 1 µM staurosporine for 16 h were stained with 1 µg/ml propidium iodide (PI) for 20 min at 37℃ and analyzed by flow cytometry. The percentage of PI positive (PI^+) cells indicating cell death are shown in (**B**). Data are means ± SD (*** = p < 0.001).

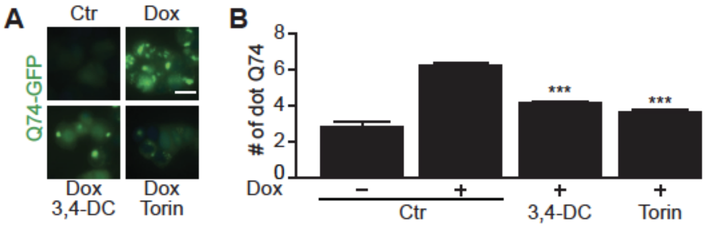

Appendix Figure S2

Appendix Fig S2 3,4-DC induces the turnover of autophagic cargo **(A,B)** PC12 pheochromocytoma cells expressing doxycycline (Dox)-inducible polyglutamine-74 (Q74)-tagged GFP were treated induced with Dox for 8 h and then treated with 3,4-DC for additional 48 h. Fluorescent micrographs were acquired and data was analyzed using automated segmentation software. Data are means ± SD *** = $p < 0.001$ versus Ctr/Dox). Representative images are shown in **(A)**. Scale bar equals 10 µm.

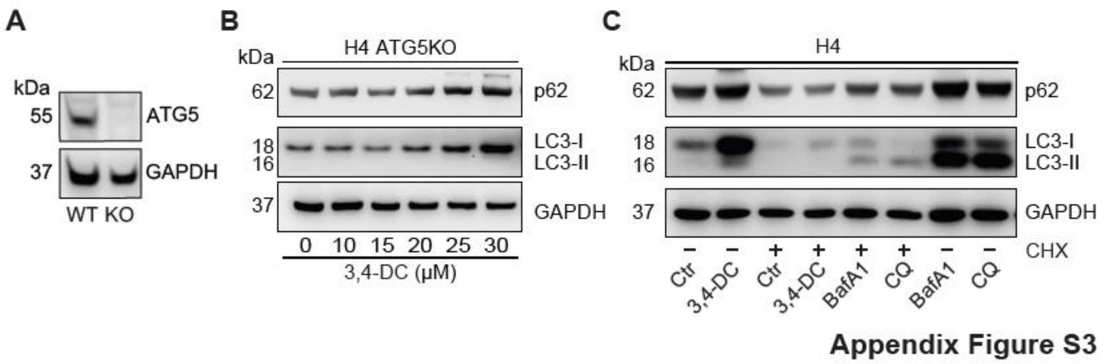

Appendix Fig S3 3,4-DC induces transcription-dependent p62 and LC3 expression **(A,B)** Atg5 knockout (Atg5KO) H4 cells were treated with the indicated increasing doses of 3,4-DC for 16 h. SDS-PAGE and immunoblots were performed to detect LC3, p62, and GAPDH protein levels. **(C)** H4 cells were treated with 30 µM 3,4-DC in the presence or absence of cycloheximide (CHX) for 8 h with bafilomycin A1 (BafA1) and chloroquine (CQ) as controls, as indicated. LC3, p62 and GAPDH protein levels were measured by SDS-PAGE and immunoblot. Samples for immunoblots in A-C were run together, then cut into stripes and probed separately.

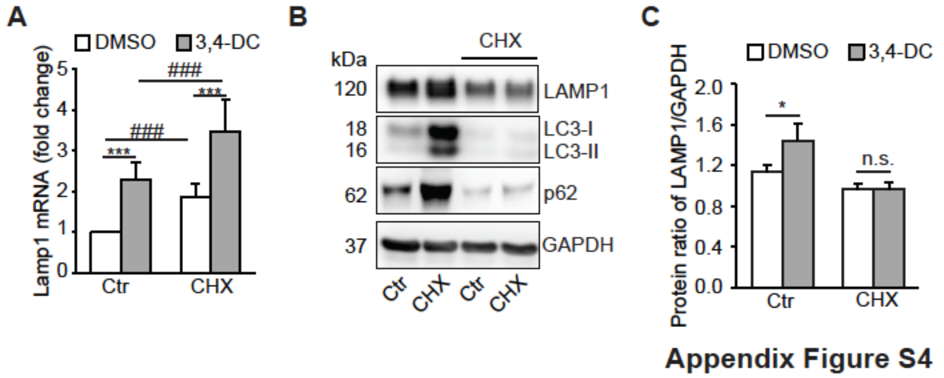

Appendix Fig S4 3,4-DC increases LAMP1 expression **(A)** U2OS cells were treated with 3,4-DC in the presence or absence of cycloheximide (CHX), and then RNA was extracted, followed by cDNA synthesis. Quantitative real time PCR was performed to measure lamp1 mRNA level with GAPDH as a loading control. Data are means ± SD (*** = $p < 0.001$; ###=$p<0.001$). **(B, C)** U2OS cells were treated as in **(A)**, and then cells were collected and processed for western

blot. LAMP1, LC3, p62, and GAPDH protein levels were measured with the respective antibodies. Bands intensities of LAMP1 and GAPDH were measured and their ratio was calculated in **(C)**. Data are means ± SEM of at least three independent experiments (* = p < 0.05). Samples for immunoblots in B were run in parallel blots, then cut into horizontal stripes and probed separately.

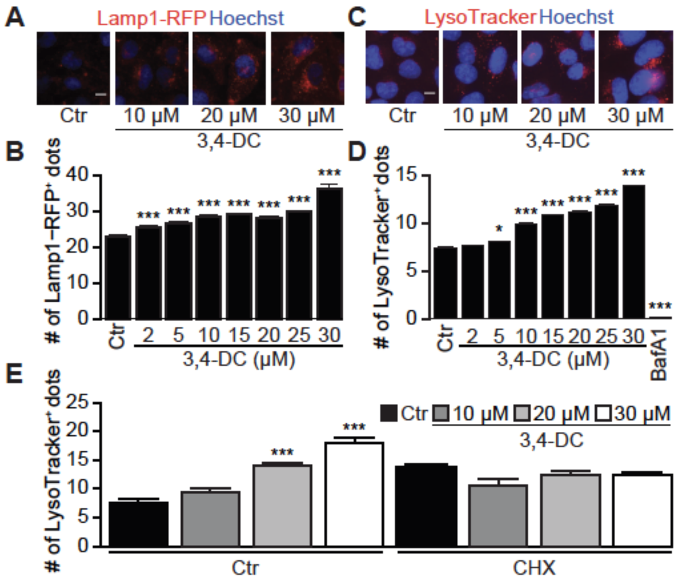

Appendix Figure S5

Appendix Fig S5 3,4-DC increases lysosomal biogenesis **(A,B)** U2OS cells stably expressing Lamp1-RFP were treated with indicated rising doses of 3,4-DC for 24 h, and then Lamp1-RFP dots **(B)** was measured to indicate the quantity of lysosomes. Data are means ± SD (** = $p < 0.01$; *** = $p < 0.001$). Representative images are shown in **(A)**. Scale bar equals 10 µm. **(C-E)** U2OS cells treated with mounting concentrations of 3,4-DC in the presence or absence of CHX as indicated were stained with LysoTracker Red for 30 min. BA1 was used as a negative control, as it inhibits lysosomal acidification (**D**). Thereafter, the red (positive) dots was measured **(D, E)**. Data are means ± SD (* = $p < 0.05$; ** = $p < 0.01$; *** = $p < 0.001$). Representative images are shown in **(C)**. Scale bar equals 10 µm.

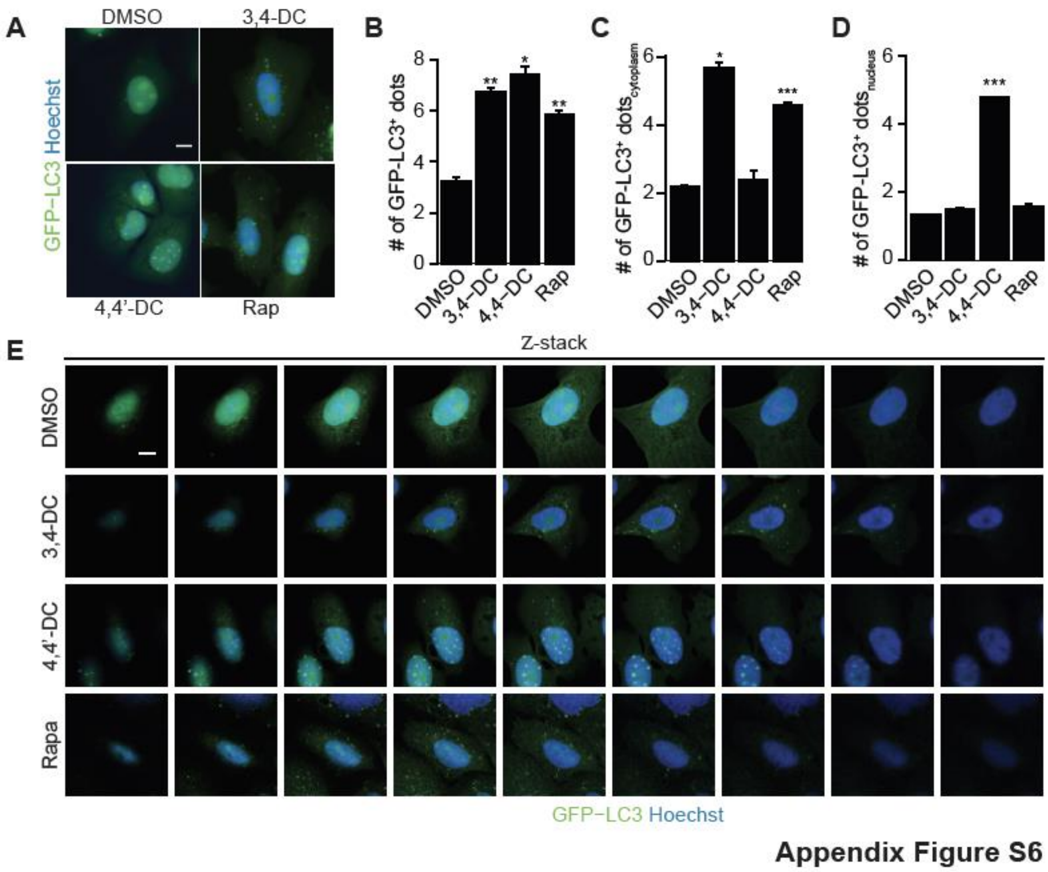

Appendix Fig S6 3,4-DC and 4,4'-DC induce distinct phenotypes (**A-E**) U2OS-GFP-LC3 cells were treated with vehicle (DMSO), 3,4-DC, 50 µM 4,4'-DC, or Rapamycin (Rap) for 16 h. The cells were fixed with PFA. Images were acquired with wide field microscopy (**A-D**). Cytoplasmic (**C**), nuclear (**D**), and total (**B**) GFP-LC3 dots were counted. Data are means ± SD (* = $p < 0.05$, ** = $p < 0.01$, *** = $p < 0.001$ vs DMSO). Representative images are shown in (**A**). Scale bars equal 10 µm. In addition to wide field imaging confocal microscopy was performed to acquire Z-stacks which are shown in (**E**).

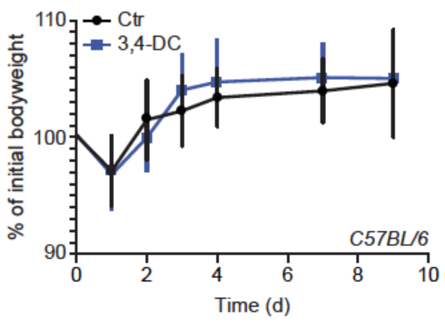

Appendix Figure S7

Appendix Fig S7: 3,4-DC is non-toxic and well tolerated in vivo. **(A)** C57/BL6 animals were i.p. injected with 230 mg/kg 3,4-DC or vehicle (Ctr) every other day. Animals were observed regularly and the body mass was monitored as an indicator for toxicity (n=6).

Appendix Table S**1**: List of agents used for HTS screen

Appendix Table 1
Compounds
Hinokiflavone
Luteolin
Fisetin
Luteolin−4'−O−glucoside
3,6−Dihydroxyflavone
4−Hydroxychalcone
7−Hydroxyflavonol
Isosakuranetin
Ornithine
Cysteamine
Trigonellin
Geraldol
Flavanone
Kaempferol−3,4',7−trimethylether
Didymin
Butein
3' Me EC
Caffein
Fortunellin
Neodiosmin
6−Methoxyflavone
Isorhamnetin
Karanjin
Datiscin
Tricetin
Tepa
Fustin
Naringenin−7−O−glucoside
Spiraeoside
EC−4' Gluc
5,6−Benzoflavone
Homoeriodictyol
7,8−Benzoflavone
Isorhoifolin
Poncirin
3,4dimethoxycinnamic
Apigenin−7−O−glucoside
Bavachinin
Isovitexin
Pratol
Deta

Quercetin−3−O−glucose−6''−acetate
Glycitein
Kaempferol−7−O−neohesperidoside
Marein
Isorhamnetin−3−O−glucoside
Luteolin−3',7−di−O−glucoside
Quercetin−3−O−glucopyranoside
3',4',7,8−Tetramethoxyflavone
Dihydrorobinetin
5,7−Dimethoxyflavanone
4'−Methoxyflavanone
Teta
DTPA
Pyrogallol
2',6'−Dihydroxy−4−methoxychalcone−4'−O−neohesperidoside
Agmatine
Trilobatin
Quercetin−3,4'−di−O−glucoside
Datiscetin
4'−Me Epic
Phloretin
4',6,7−Trihydroxyisoflavone
N−2−aminoethylpropanediamine
4',7−Dimethoxyisoflavone
catechol (1,2 dihydroxybenzene)
EC 4 sulfate
7−Methoxyflavone
Luteolin−7−O−glucoside
Cystamine
Eupatorin−5−methylether
Quercetagetin
Dihydromyricetin
Cupressuflavone
6,7 Dihydroxyflavone
3'−Me EC7S
Diaminocyclohexane
Kaempferol−3−O−glucosideNarirutin
4'−Hydroxyflavanone
Putrescine
3',4',7−Trimethoxyflavone
Baicalein−7−methylether
7−Hydroxy−5−methylflavone
Maritimein
Glycitin
EC 7 sulfate

Ethylenediamine
gallic acid
5 hydroxymethylfurfural
Genistein−4',7−dimethylether
3',4'Dimethoxyflavone
3',4',7,8−Tetrahydroxyflavone
Kaempferide
Saponarin
LiquiritigeninIsorhamnetin−3−O−rutinoside
4−Deoxyphloridzin
Neohesperidin dihydrochalcone
Hexamethylenetetramine
Peha
Epigallocatechingallate
3',4',7−Trihydroxyflavone
Isoliquiritigenin
Baicalein−5,6,7−trimethylether
Ononin
chlorogenic acid
Quercetin−3,4,7,3',4'−pentamethylether
2'−Methoxyflavone
Neoeriocitrin
Robinin
3,4 dihydroxycinnamic
Rhamnetin
N−ethylenediamine
Sissotrin
Flavanomarein
3,3, diamino−N−methyldipropylamine
Trienthine
Genistin
(−)−Homoeriodictyol
Gardenin A
Eriodictyol
caffeic acid
7,8−Dimethoxyflavone
6−Methoxyflavanone
2'−Hydroxyflavanone
Flavone
Quercetagetin−7−O−glucoside
EC−3'Sulfate
Hexemethylenediamine
3 meEC 5Sulfate
Cadaverine
Laricitrin

Kaempferol−3−O−rutinoside
Myricitrin
3',4',5,7−Tetrahydroxy−3−methoxyflavone
Eupatorin
nicotinamide
Eriocitrin
Epicatechin 3' Glucuronide
Ipriflavone
3'−Me EC 4' Sulfate
4',6,7−Trimethoxyisoflavone
Ethanolamine
7−Hydroxyflavanone
Bis 3 aminopropilamine
7−Methoxyflavonol
Tannic acid
6−Hydroxyflavanone
5−Methoxyflavanone
6−Methoxyflavonol
Penicillamine
Nicotinic Acid
3−Methoxyflavone
1,3 diaminopropane
Pinocembrin−7−methylether
6−Methoxyluteolin
3',4'−Dihydroxyflavone
Homobutein
5−Methyl−7−methoxyisoflavone
Chrysoeriol
Pinocembrin
5−Methoxyflavone
Apigenin−4',5,7−trimethylether
Syringetin
Spermine
Luteolin tetramethylether
3,7−Dihydroxy−3',4',5'−trimethoxyflavone
3',4',5,5',6,7−Hexamethoxyflavonepiceatannol
3',5,7−Trihydroxy−3,4'−dimethoxyflavone
Rhamnazin
(+/−)−Equol
3,4−Dimethoxychalcone
Spermidine
4−Methoxychalcone
4'−Hydroxychalcone
3,5,7−Trihydroxy−3',4',5'−trimethoxyflavone
Prunetin

2',4−Dihydroxy−4',6'−dimethoxychalcone
2−Hydroxychalcone

Appendix Table S2: Abbreviations of the chalcones analyzed in this study

Appendix Table 2	
Abbreviation	Drug names
	Chalcone
	2',4-Dihydroxy-4',6'-dimethoxychalcone(Flavokawain C)
2',6'-D-4,4'-DC	2',6'-Dihydroxy-4,4'-dimethoxychalcone
2',6'-D-4-MC-4'-O-nH	2',6'-Dihydroxy-4-methoxychalcone-4'-O-neohesperidoside
3,4-DC	3,4-Dimethoxychalcone
4,4'-DC	4,4'-Dimethoxychalcone
2,3-D-2'HC	2,3-Dimethoxy-2'-hydroxychalcone
2-HC	2-Hydroxychalcone
2'-HC	2'-Hydroxychalcone
4-HC	4-Hydroxychalcone
4'-HC	4'-Hydroxychalcone
	2'-Hydroxy-4,4',6'-trimethoxychalcone(Flavokawain A)
4-MC	4-Methoxychalcone
4'-MC	4'-Methoxychalcone
NH DHC	Neohesperidin dihydrochalcone
	2',3,3',4,4'-Pentahydroxy-4'-glucosylchalcone(Marein)
	2',3,4,4'-Tetrahydroxychalcone (Butein)
	2',4,4',6'-Tetrahydroxydihydrochalcone (Phloretin)
	2',4,4'-Trihydroxychalcone (Isoliquiritigenin)
	2',4,4'-Trihydroxy-3-methoxychalcone (Homobutein)
	Phloridzin (Phloretin-2'-O-glucoside)
	Trilobatin (Phloretin-4'-O-glucoside)
	Sieboldin (Phloretin-3-hydroxy-4'-O-glucoside)
4-D-Phloridzin	4-Deoxyphloridzin
EC	Eriodictyolchalcone (2',4',6',3,4-Pentahydroxychalcone)

Appendix Table S3: List of utilized RT-PCR primers.

Appendix Table 3		
Gene Name	Forward Primer(5'-3')	Reverse Primer(5'-3')
Lamp1	TCTCAGTGAACTACGACACCA	AGTGTATGTCCTCTTCCAAAAGC
Lamp2	GAAAATGCCACTTGCCTTTATGC	AGGAAAAGCCAGGTCCGAAC
Ulk1	GGCAAGTTCGAGTTCTCCCG	CGACCTCCAAATCGTGCTTCT
Atg14	TTCAGAGGCATAATCGCAAACT	CCAGACGCTCATAATGACTTCTT
Atg9B	TGTGCTCACCGTCTACGAC	GGGAGGTAGTGCATGTGGG
Atg9A	CCAGAACTACATGGTGGCACT	GTCCCCAGAAGAGGATCAGC
Atg5	AAAGATGTGCTTCGAGATGTGT	CACTTTGTCAGTTACCAACGTCA
Atg7	ATGATCCCTGTAACTTAGCCCA	CACGGAAGCAAACAACTTCAAC

Sqstm1/p62	AAGCCGGGTGGGAATGTTG	CCTGAACAGTTATCCGACTCCAT
LC3A	AACATGAGCGAGTTGGTCAAG	GCTCGTAGATGTCCGCGAT
LC3B	AAGGCGCTTACAGCTCAATG	CTGGGAGGCATAGACCATGT
LC3C	GAGCCACGGAAGCCTTTTACT	TGGGAGGCGTAGGTCATGT
UVRAG	ATGCCAGACCGTCTTGATACA	TGACCCAAGTATTTCAGCCCA
WIPI1	AGTCAGTCACACAAAACCACG	AGAGCACATAGACCTGTTGGG
WIPI2	CCATCGTCAGCCTTAAAGCAC	TCCAGGCATACTATCAGCCTC

Appendix Table S4: Statistics.

Appendix Table 4				
Figures	Groups	Symbol	p-Value	n
Figure 1F	Rapa (vs. Ctr)	***	<0.001	4
	2,3-D-2'-HC (vs. Ctr)	***	<0.001	4
	2-HC (vs. Ctr)	***	0.00015	4
	2'-HC (vs. Ctr)	**	0.0020	4
	3,4-DC (vs. Ctr)	***	<0.001	4
	Butein (vs. Ctr)	**	0.0056	4
	4-MC (vs. Ctr)	**	0.0071	4
Figure 1H	Rapa(vs. Ctr)	***	0.00060	4
	2'-HC(vs. Ctr)	*	0.025	4
	4-HC(vs. Ctr)	**	0.0024	4
	4'-HC(vs. Ctr)	**	0.0031	4
	4'-MC(vs. Ctr)	**	0.0032	4
	3,4-DC(vs. Ctr)	**	0.0047	4
	Phloretin(vs. Ctr)	*	0.012	4
	Butein(vs. Ctr)	*	0.018	4
	Homobutein(vs. Ctr)	**	0.0011	4
	4-MC(vs. Ctr)	*	0.025	4
	4,4'-DC(vs. Ctr)	*	0.034	4
Figure 1J	DMSO-KIC (vs. DMSO-Ctr)	***	<0.001	6
	3,4-DC-DCA(vs. 3,4-DC-Ctr)	##	0.0030	6
	3,4-DC-KIC(vs. 3,4-DC-Ctr)	###	0.00011	6
	3,4-DC-Leu(vs. 3,4-DC-Ctr)	###	<0.001	6
Figure 1K	3,4-DC-DCA(vs. 3,4-DC-Ctr)	###	<0.001	6
	3,4-DC-KIC(vs. 3,4-DC-Ctr)	###	<0.001	6
	3,4-DC-Leu(vs. 3,4-DC-Ctr)	###	<0.001	6
Figure 2B (LC3-II /GAPDH)	3,4-DC 20μM (vs 0 μM)	*	0.048	3
	3,4-DC 25μM(vs 0 μM)	*	0.030	3
	3,4-DC 30μM(vs 0 μM)	*	0.031	3
Figure 2B (p62 /GAPDH)	3,4-DC 20μM (vs 0 μM)	#	0.014	3
	3,4-DC 25μM(vs 0 μM)	#	0.011	3
	3,4-DC 30μM(vs 0 μM)	#	0.028	3
Figure 2D (LC3-II/LC3-I)	1h (vs. 0h)	$	0.023	3
	2h (vs. 0h)	$	0.017	3

Figure 2D (p62/GAPDH)	1h (vs. 0h)	###	0.00086	3
	2h (vs. 0h)	#	0.028	3
	6h (vs. 0h)	#	0.021	3
	8h (vs. 0h)	#	0.014	3
Figure 2D (LC3-II /GAPDH)	1h (vs. 0h)	*	0.035	3
	4h (vs. 0h)	*	0.037	3
	6h (vs. 0h)	**	0.0092	3
	8h (vs. 0h)	**	0.0055	3
Figure 2F	3,4-DC (vs. Ctr)	*	0.011	3
	CQ (vs. Ctr)	**	0.0018	3
	CQ/3,4-DC (vs. CQ)	##	0.0030	3
Figure 2H	3,4-DC(vs. Ctr)	***	<0.001	4
	Rapa(vs. Ctr)	***	<0.001	4
	CQ/3,4-DC(vs. CQ)	###	<0.001	4
	CQ/Rapa(vs. CQ)	###	<0.001	4
Figure 2J (GFP+)	3,4-DC/5μM(vs. Ctr)	##	0.0072	4
	Rapa (vs. Ctr)	#	0.025	4
	CQ(vs. Ctr)	##	0.0020	4
	BA1(vs. Ctr)	###	0.00021	4
Figure 2J (GFP-)	3,4-DC/5μM(vs. Ctr)	***	0.00098	4
	3,4-DC/10μM(vs. Ctr)	***	0.00060	4
	3,4-DC/20μM(vs. Ctr)	**	0.0014	4
	3,4-DC/30μM(vs. Ctr)	***	<0.001	4
	Rapa(vs. Ctr)	***	0.00091	4
	CQ(vs. Ctr)	***	0.00015	4
	BA1(vs. Ctr)	***	<0.001	4
Figure 3D	3,4-DC/10μM(vs. Ctr)	**	0.0045	3
	3,4-DC/20μM(vs. Ctr)	*	0.042	3
	3,4-DC/30μM(vs. Ctr)	*	0.013	3
	3,4-DC/5μM(vs. CQ)	#	0.028	3
	3,4-DC/10μM(vs. CQ)	#	0.031	3
	3,4-DC/20μM(vs. CQ)	#	0.034	3
	3,4-DC/30μM(vs. CQ)	#	0.034	3
Figure 3E	Atg14(vs. Ctr)	*	0.046	3
	Atg9A(vs. Ctr)	*	0.047	3
	Lamp1(vs. Ctr)	*	0.028	3
	LC3B(vs. Ctr)	***	<0.001	3
	Sqstm1(vs. Ctr)	***	<0.001	3
	Ulk1(vs. Ctr)	**	0.0043	3
Figure 3G	Torin(vs. Ctr)	**	0.0030	4
Figure 4A	2-HC(vs. Ctr)	**	0.0017	4
	Chalcone(vs. Ctr)	***	0.00013	4
	2,3-D-2'-HC(vs. Ctr)	***	0.00012	4
	4’-MC(vs. Ctr)	***	<0.001	4
	EBSS(vs. Ctr)	***	<0.001	4
	2’-HC(vs. Ctr)	***	<0.001	4

	3,4-DC(vs. Ctr)	**	0.0023	4
	4-MC(vs. Ctr)	*	0.016	4
	4-HC(vs. Ctr)	**	0.0098	4
	4'-HC(vs. Ctr)	***	0.00076	4
	4,4'-DC(vs. Ctr)	*	0.013	4
	4-D-Phloridzin(vs. Ctr)	*	0.014	4
	Flavokawain C(vs. Ctr)	***	0.00066	4
	Flavokawain A(vs. Ctr)	**	0.0021	4
Figure 4D	3,4-DC (vs. Ctr)	***	0.00018	4
Figure 4H	3,4-DC/WT (vs. Ctr/WT)	***	<0.001	8
	3,4-DC/TSC2KO(vs. Ctr/TSC2KO)	***	<0.001	8
	3,4-DC/TSC2KO(vs. 3,4-DC/WT)	###	<0.001	8
Figure 4K	3,4-DC/WT (vs. Ctr/WT)	**	0.0060	3
	3,4-DC/TSC2KO(vs. 3,4-DC/WT)	##	0.0085	3
Figure 4L	3,4-DC/WT (vs. Ctr/WT)	***	0.00038	3
	3,4-DC/TSC2KO(vs. Ctr/TSC2KO)	**	0.0031	3
	3,4-DC/TSC2KO(vs. 3,4-DC/WT)	#	0.026	3
Figure 4N	Torin	*	0.022	3
	3,4-DC	*	0.043	3
Figure 5C	siTFEB-1/3,4-DC(vs. siCtr/3,4-DC)	**	0.0012	3
	siTFEB-2/3,4-DC(vs. siCtr/3,4-DC)	**	0.0030	3
	siTFEB-3/3,4-DC(vs. siCtr/3,4-DC)	*	0.034	3
Figure 5F	3,4-DC/15μM/KO(vs. 3,4-DC/15μM/WT)	*	0.031	3
	3,4-DC/20μM/KO(vs. 3,4-DC/20μM/WT)	*	0.038	3
	3,4-DC/30μM/KO(vs. 3,4-DC/30μM/WT)	*	0.012	3
Figure 5H	3,4-DC/WT(vs. Ctr/WT)	***	0.00036	4
	3,4-DC/TFEBKO(vs. Ctr/ TFEBKO)	**	0.0050	4
	3,4-DC/TFE3KO(vs. Ctr/ TFE3KO)	*	0.016	4
Figure 5I	3,4-DC/WT(vs. Ctr/WT)	***	0.00023	4
	3,4-DC/TFEBKO(vs. Ctr/ TFEBKO)	**	0.0058	4
	3,4-DC/TFE3KO(vs. Ctr/ TFE3KO)	*	0.037	4
	3,4-DC/TF DKO(vs. Ctr/ TF DKO)	*	0.046	4
Figure 5J (LC3B)	3,4-DC/WT(vs. Ctr/WT)	**	0.0029	3
	3,4-DC/TF DKO(vs. 3,4-DC/WT)	#	0.031	3
Figure 5J	3,4-DC/WT(vs. Ctr/WT)	***	0.00011	3

(p62)	3,4-DC/TF DKO(vs. 3,4-DC/WT)	##	0.0087	3
Figure 5J (lamp1)	3,4-DC/WT(vs. Ctr/WT)	*	0.011	3
	3,4-DC/TF DKO(vs. 3,4-DC/WT)	#	0.046	3
Figure 6B	TFEB/3,4-DC (vs. Ctr)	*	0.048	3
	TFE3/3,4-DC (vs. Ctr)	**	0.0051	3
Figure 6D	TFEB/3,4-DC (vs. Ctr)	**	0.0076	3
	TFE3/3,4-DC (vs. Ctr)	*	0.019	3
Figure 6F	3,4-DC (vs. Ctr)	*	0.048	3
	Leu (vs. Ctr)	*	0.016	3
	3,4-DC/Leu (vs. Leu)	#	0.030	3
Figure 6J	3,4-DC (vs. Ctr)	*	0.028	3
	Leu (vs. Ctr)	*	0.041	3
	3,4-DC/Leu (vs. Leu)	#	0.010	3
Figure 6H	3,4-DC (vs. Ctr)	**	0.0010	3
Figure 6L	3,4-DC (vs. Ctr)	*	0.044	3
Figure 7B	3,4-DC/WT (vs. Ctr/WT)	**	0.0026	5
	Ctr/Atg7 cKO (vs. Ctr/WT)	##	0.0093	3
Figure 8C	MTX (vs. Ctr)	*	0.012	8
	3,4-DC/MTX (vs. MTX)	##	0.0073	10
Figure 8E	MTX (vs. Ctr)	***	0.00097	8
	3,4-DC/MTX (vs. MTX)	#	0.025	10
EV 1A	3,4-DC (vs. Ctr)	**	0.0060	3
EV 1D	3,4-DC (vs. Ctr)	***	0.00040	5
EV 1F (p62/GAPDH)	1h (vs. 0h)	##	0.0015	3
	2h (vs. 0h)	##	0.0044	3
EV 1F (LC3-II /GAPDH)	1h(vs. 0h)	*	0.031	3
	2h(vs. 0h)	**	0.0039	3
	4h(vs. 0h)	**	0.0052	3
	6h(vs. 0h)	*	0.017	3
	8h(vs. 0h)	*	0.028	3
EV 1J	3,4-DC (vs. Ctr)	***	0.00023	4
	Rapa (vs. Ctr)	***	0.00049	4
EV 2G	3,4-DC/20μM (vs. Ctr)	*	0.030	3
	3,4-DC/30μM (vs. Ctr)	***	0.00017	3
	3,4-DC/20μM (vs. CQ)	###	0.00016	3
	3,4-DC/30μM (vs. CQ)	##	0.0011	3
EV 2H	Atg14 (vs. Ctr)	*	0.042	3
	Atg9B(vs. Ctr)	**	0.0028	3
	Lamp1(vs. Ctr)	***	0.00076	3
	LC3B(vs. Ctr)	**	0.0098	3
	Sqstm1(vs. Ctr)	***	<0.001	3
	Ulk1(vs. Ctr)	***	<0.001	3
	WIPI-1(vs. Ctr)	**	0.0020	3
EV 2I	3,4-DC(vs. Ctr)	***	<0.001	6
	Rapa(vs. Ctr)	***	<0.001	6
	Resv(vs. Ctr)	***	<0.001	6
	Spd(vs. Ctr)	***	<0.001	6

	Torin(vs. Ctr)	***	<0.001	6
	Rapa/CHX(vs. CHX)	###	<0.001	6
	Resv/CHX(vs. CHX)	###	0.00010	6
	Spd/CHX(vs. CHX)	###	<0.001	6
	Torin/CHX(vs. CHX)	###	<0.001	6
EV 3B	Spd(vs. Ctr)	***	<0.001	6
	Torin(vs. Ctr)	***	<0.001	6
EV 3C	Spd(vs. Ctr)	***	<0.001	5
	Torin(vs. Ctr)	*	0.035	5
EV 3E	3,4-DC (vs. Ctr)	***	<0.001	4
	Torin(vs. Ctr)	***	0.00018	4
EV 4B	4,4-DC/WT (vs. DMSO/WT)	***	<0.001	3
EV 4D	4,4-DC/WT (vs. DMSO/WT)	***	<0.001	3
EV 4F	3,4-DC/WT (vs. DMSO/WT)	**	0.0011	6
	4,4-DC/WT (vs. DMSO/WT)	***	<0.001	6
	4,4-DC/TF DKO (vs. DMSO/ TF DKO)	###	<0.001	6
EV 4G	3,4-DC/WT (vs. DMSO/WT)	***	0.00044	6
	4,4-DC/WT (vs. DMSO/WT)	***	0.00037	6
	4,4-DC/TF DKO (vs. DMSO/ TF DKO)	###	<0.001	6
EV 4H	4,4-DC/WT (vs. DMSO/WT)	***	<0.001	6
	4,4-DC/TF DKO (vs. DMSO/ TF DKO)	###	0.00038	6
EV 4J	siGATA2-1/4,4-DC(vs. SiCtr/4,4-DC)	**	0.0022	4
	siGATA2-2/4,4-DC(vs. SiCtr/4,4-DC)	*	0.017	4
EV 4K	siGATA2-1/4,4-DC(vs. SiCtr/4,4-DC)	**	0.0021	4
	siGATA2-2/4,4-DC(vs. SiCtr/4,4-DC)	*	0.021	4
EV 4L	siGATA2-1/4,4-DC(vs. SiCtr/4,4-DC)	*	0.013	4
	siGATA2-2/4,4-DC(vs. SiCtr/4,4-DC)	*	0.025	4
Fig S1B	STS (vs. Ctr)	***	<0.001	3
Fig S2B	3,4-DC/Dox (vs. Dox)	***	<0.001	8
	Torin/Dox (vs. Dox)	***	<0.001	8
Fig S4A	3,4-DC (vs. DMSO)	***	0.00013	8
	3,4-DC/CHX (vs. CHX)	***	0.00035	8
	CHX (vs. DMSO)	###	0.00013	8
	3,4-DC/CHX (vs. 3,4-DC)	###	0.00035	8
Fig S4C	3,4-DC (vs. DMSO)	*	0.027	4
	3,4-DC/CHX (vs. CHX)	ns	0.43	4
Fig S5B	3,4-DC/2µM (vs. Ctr)	***	0.00054	4
	3,4-DC/5µM (vs. Ctr)	***	0.00013	4
	3,4-DC/10µM (vs. Ctr)	***	0.00055	4
	3,4-DC/15µM (vs. Ctr)	***	<0.001	4
	3,4-DC/20µM (vs. Ctr)	***	0.00010	4
	3,4-DC/25µM (vs. Ctr)	***	<0.001	4
	3,4-DC/30µM (vs. Ctr)	***	<0.001	4

Fig S5D	3,4-DC/5µM (vs. Ctr)	*	0.025	4
	3,4-DC/10µM (vs. Ctr)	***	0.00034	4
	3,4-DC/15µM (vs. Ctr)	***	0.00031	4
	3,4-DC/20µM (vs. Ctr)	***	<0.001	4
	3,4-DC/25µM (vs. Ctr)	***	<0.001	4
	3,4-DC/30µM (vs. Ctr)	***	<0.001	4
Fig S5E	3,4-DC/20µM (vs. Ctr)	***	0.00025	5
	3,4-DC/30µM (vs. Ctr)	***	0.00021	5
Fig S6B	3,4-DC (vs. DMSO)	**	0.0042	3
	4,4-DC (vs. DMSO)	*	0.036	3
	Rap (vs. DMSO)	**	0.0052	3
Fig S6C	3,4-DC (vs. DMSO)	*	0.014	3
	Rap (vs. DMSO)	***	0.00084	3
Fig S6D	4,4-DC (vs. DMSO)	***	0.00020	3

Appendix Table S5: Statistics for tumor growth curves

Appendix Table 5 Tumor Growth : Two-way ANOVA, Dunnett's multiple comparisons test					
Figures	Compared Groups	Mean Diff.	95% Cl of diff.	Significant?	Symbol
Figure 8F	MTX vs. Ctr	-86	-130.8 to -41.21	Yes	***
	Ctr vs. 3,4-DC+MTX	141.6	77.28 to 205.9	Yes	***
	MTX vs. 3,4-DC+MTX	55.6	12.77 to 98.42	Yes	##
Figure 8G	MTX vs. Ctr	-72.49	-98.82 to -46.16	Yes	***
	Ctr vs. 3,4-DC+MTX	69.63	43.30 to 95.96	Yes	***
	MTX vs. MTX/3,4-DC	-2.855	-27.02 to 21.31	No	ns
Figure 8I	MTX vs. Ctr	-56.23	-79.57 to -32.89	Yes	***
	Ctr vs. 3,4-DC+MTX	72.04	48.34 to 95.75	Yes	***
	MTX vs. 3,4-DC+MTX	15.82	-5.893 to 37.53	No	ns
Figure 8J	MTX vs. Ctr	-97.09	-123.4 to -70.81	Yes	***
	Ctr vs. 3,4-DC+MTX	137.7	111.9 to 163.5	Yes	***
	MTX vs. 3,4-DC+MTX	40.61	15.5 to 65.72	Yes	###
Figure 8L	MTX vs. Ctr	-88.03	-110.7 to -65.31	Yes	***
	Ctr vs. 3,4-DC+MTX	78.81	56.80 to 100.8	Yes	***
	MTX vs. 3,4-DC+MTX	-9.224	-29.49 to 11.04	No	ns
EV 5B	OXA vs. Ctr	-148.8	-167.2 to -130.4	Yes	***
	Ctr vs. 3,4-DC+OXA	192.5	174.1 to 210.9	Yes	***
	OXA vs. 3,4-DC+OXA	43.7	27.22 to 60.18	Yes	###
EV 5C	OXA vs. Ctr	-74.97	-107 to -42.96	Yes	***
	Ctr vs. 3,4-DC+OXA	47.75	15.74 to 79.76	Yes	**
	OXA vs. 3,4-DC+OXA	-27.21	-59.22 to 4.797	no	ns
EV 5E	MTX vs. Ctr	-94.56	-117.8 to -71.35	Yes	***
	Ctr vs. 3,4-DC+MTX	139.8	117.1 to 162.4	Yes	***

	MTX vs. 3,4-DC+MTX	45.21	24.33 to 66.09	Yes	###
EV 5F	MTX vs. Ctr	-44.48	-76.89 to -12.07	Yes	**
	Ctr vs. 3,4-DC+MTX	51.38	20.15 to 82.61	Yes	***
	MTX vs. 3,4-DC+MTX	6.879	-24.34 to 38.13	no	ns

Appendix Table S6: Statistics for tumor in vivo experimentation

Appendix table 6: Number of animals									
	Figure 8F	Figure 8G	Figure 8I	Figure 8J	Figure 8L	EV1B	EV 1C	EV1E	EV 1F
Ctr	6	8	10	10	9	8	6	6	6
3,4-DC	8	9	10	10	9	8	6	6	6
MTX/OXA	9	11	14	11	12	12	6	8	6
3,4-DC+ MTX/OXA	7	11	13	12	14	12	6	9	7
