# Supplementary material for: 3,4‐Dimethoxychalcone induces autophagy through activation of the transcription factors TFE3 and TFEB
Source: EMBO Mol Med. 2019 Oct 14;11(11):e10469. doi: 10.15252/emmm.201910469 (PMC6835206; doi:10.15252/emmm.201910469)
Supplement: Supplementary file 2 — Expanded View Figures PDF [file EMMM-11-e10469-s002.pdf]

Expanded View Figures

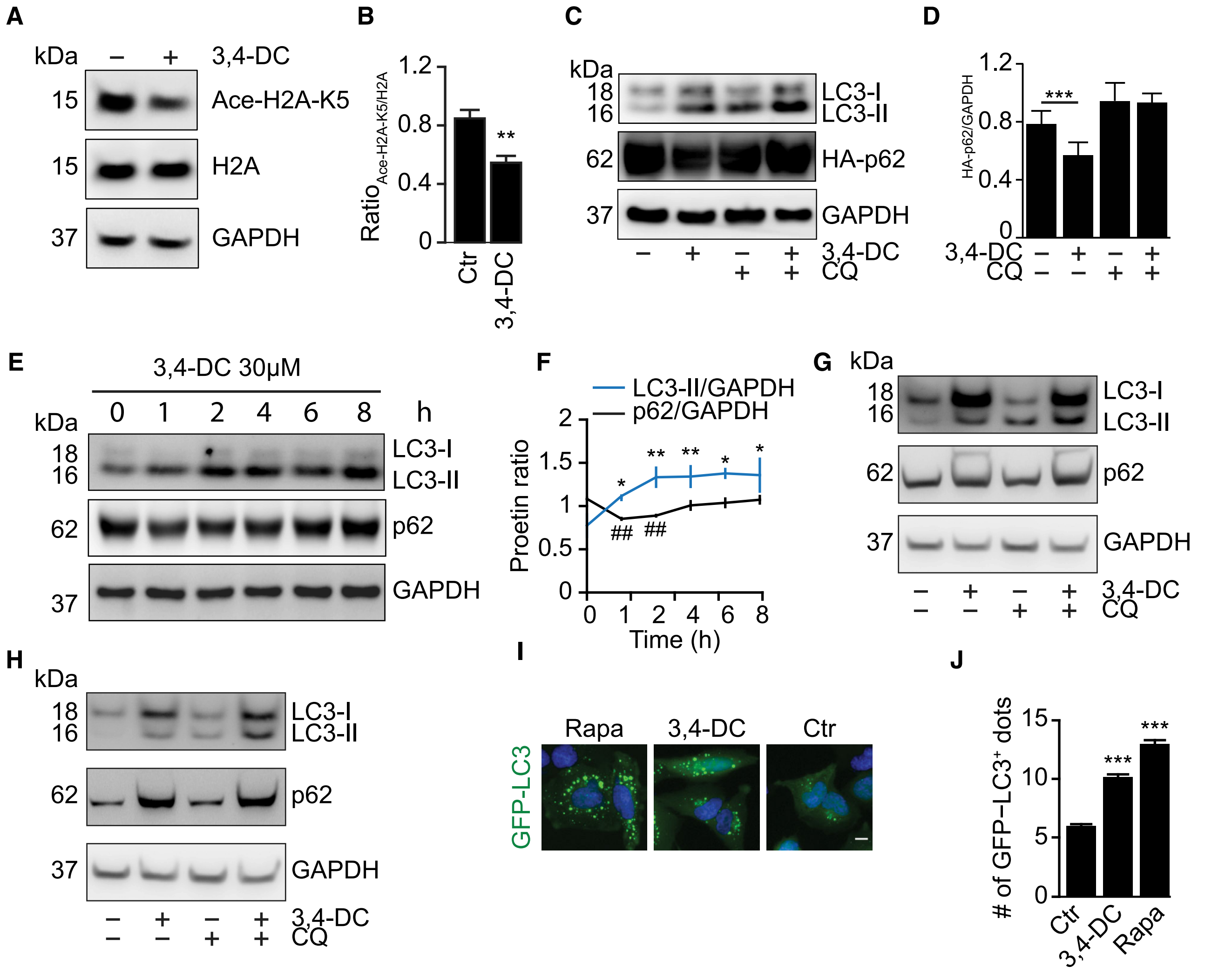

Figure EV1. 3,4-DC increases autophagic flux.

A, B HepG2 cells treated with 30 μM 3,4-DC for 8 h were collected for SDS–PAGE and immunoblot. Acetylation at lysine 5 of histone 2A (Ace-H2A-K5) was detected (A). Band intensities of Ace-H2A-K5 and histone 2A (H2A) were measured, and their ratio was calculated in (B). Data are means ± SEM of at least three independent experiments (**$P < 0.01$; Student's *t*-test).

C, D U2OS cells transfected with a plasmid expressing p62 protein fused with an HA tag (HA-p62) were treated with 30 μM 3,4-DC for 16 h in the presence or absence of CQ for 4 h. Band intensities of HA-p62 and GAPDH were assessed, and their ratio (HA/GAPDH) was calculated in (D). Data are means ± SEM of at least three independent experiments (***$P < 0.001$; Student's *t*-test).

E, F H4 cells were treated with 30 μM 3,4-DC for a time course as indicated. SDS–PAGE and immunoblot were performed to monitor LC3-I, LC3-II, p62, and GAPDH protein levels. The band intensities were measured, and the ratio of LC3-II and GAPDH or p62 and GAPDH was calculated in (F). Data are means ± SEM of at least three independent experiments (LC3-II/GAPDH: *$P < 0.05$, **$P < 0.01$; p62/GAPDH: $^{##}P < 0.01$; Student's *t*-test).

G, H H4 cells (G) or U2OS cells (H) were treated with 30 μM 3,4-DC for 16 h in the presence or absence of chloroquine (CQ) for the last 4 h. LC3, p62, and GAPDH protein levels were detected by SDS–PAGE and immunoblot.

I, J H4-GFP-LC3 cells were treated with 30 μM 3,4-DC, 10 μM rapamycin as a positive control, or left untreated for 16 h. GFP-LC3 dots were assessed to indicate autophagy activity. Data are means ± SD of four replicates (***$P < 0.001$; Student's *t*-test). Representative images are shown in (I). Scale bar equals 10 μm.

Data information: Samples for immunoblots were run on the same gel (E, G, H) or several parallel gels (A, C), then blotted, cut into horizontal stripes, and probed separately.

Source data are available online for this figure.

 © 2019 The Authors

Figure EV2. 3,4-DC triggers transcription-dependent autophagy induction.

A–C ATG5 knockout (ATG5KO) U2OS (A, B) or H4 cells (C) were treated with the indicated doses of 3,4-DC, CQ, and BafA1 (200 nM) as controls for 16 h (C). SDS–PAGE and immunoblots were performed to detect LC3, p62, and GAPDH protein levels.

D, E HepG2 cells (D) and U2OS cells (E) were treated with 30 μM 3,4-DC in the presence or absence of cycloheximide (CHX) or actinomycin D (AMD) for 8 h with CQ and BafA1 as controls, as indicated. LC3, p62, and GAPDH protein levels were measured by SDS–PAGE and immunoblot.

F, G U2OS-GFP-LC3 cells were treated with 30 μM 3,4-DC in the presence or absence of CHX, AMD, or CQ for 16 h. GFP-LC3 dots were quantified in (G). Data are means ± SD of three replicates (* $P < 0.05$, *** $P < 0.001$ vs. Ctr; ## $P < 0.01$, ### $P < 0.001$ vs. Ctr/CQ; Student's *t*-test). Representative images are shown in (F). Scale bar equals 10 μm.

H RNA expression levels of the indicated genes in U2OS cells were measured by RT–PCR and normalized to the expression of the housekeeping gene (GAPDH). Data are means ± SD of three replicates (* $P < 0.05$; ** $P < 0.01$; *** $P < 0.001$; Student's *t*-test).

I U2OS-GFP-LC3 cells were treated with 3,4-DC, rapamycin, resveratrol, spermidine, or torin1 in the presence or absence of CHX for 6 h. GFP-LC3 dots were quantified. Data are means ± SD of six replicates (*** $P < 0.001$ vs. Ctr; ### $P < 0.001$ vs. Ctr/CHX; Student's *t*-test).

Data information: Samples for immunoblots in (A–E) were run together on the same gel, then blotted, probed, stripped and reprobed (A) or cut into horizontal stripes, and probed separately (B–E).

Source data are available online for this figure.

© 2019 The Authors

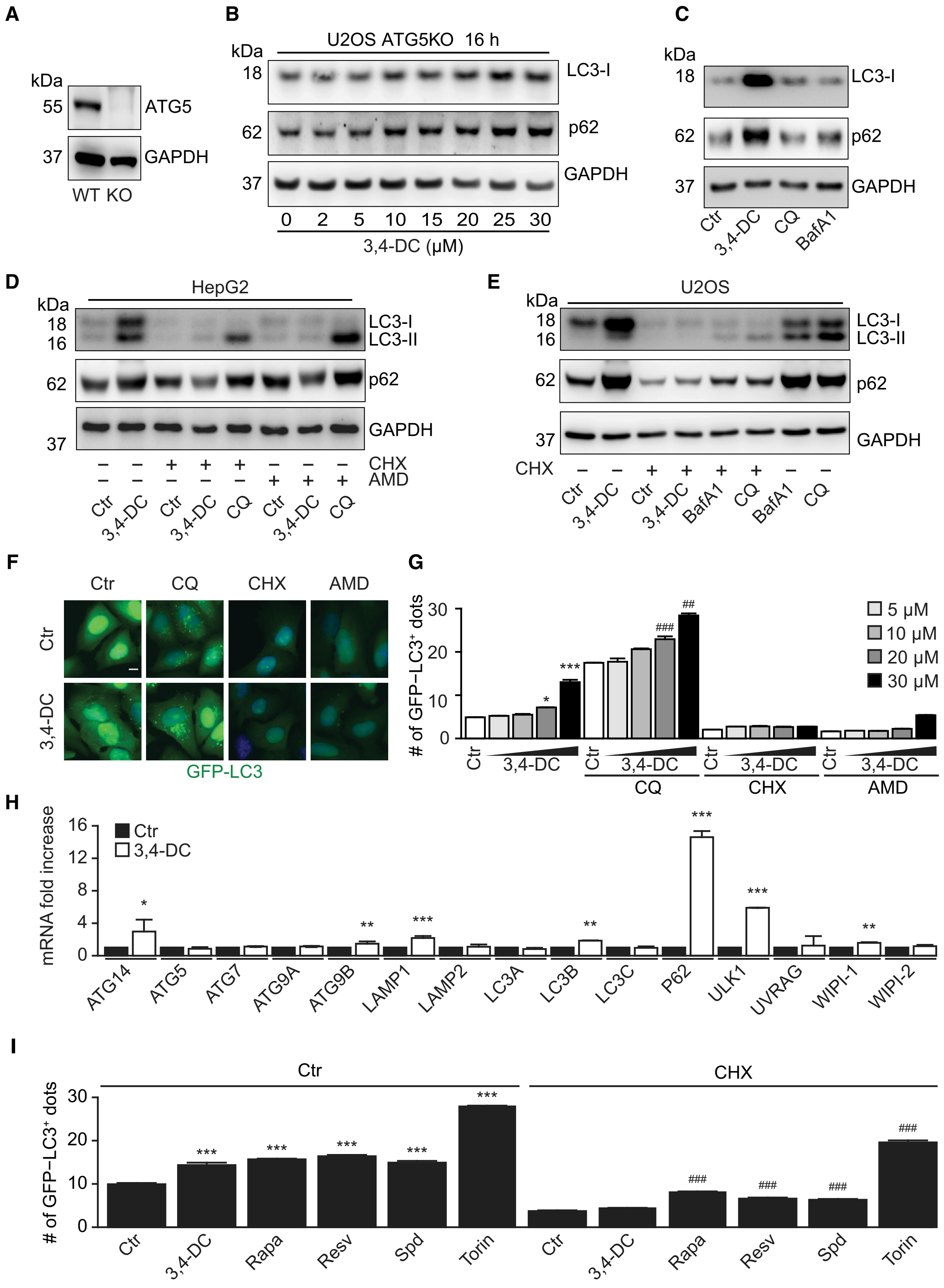

Figure EV2.

 © 2019 The Authors

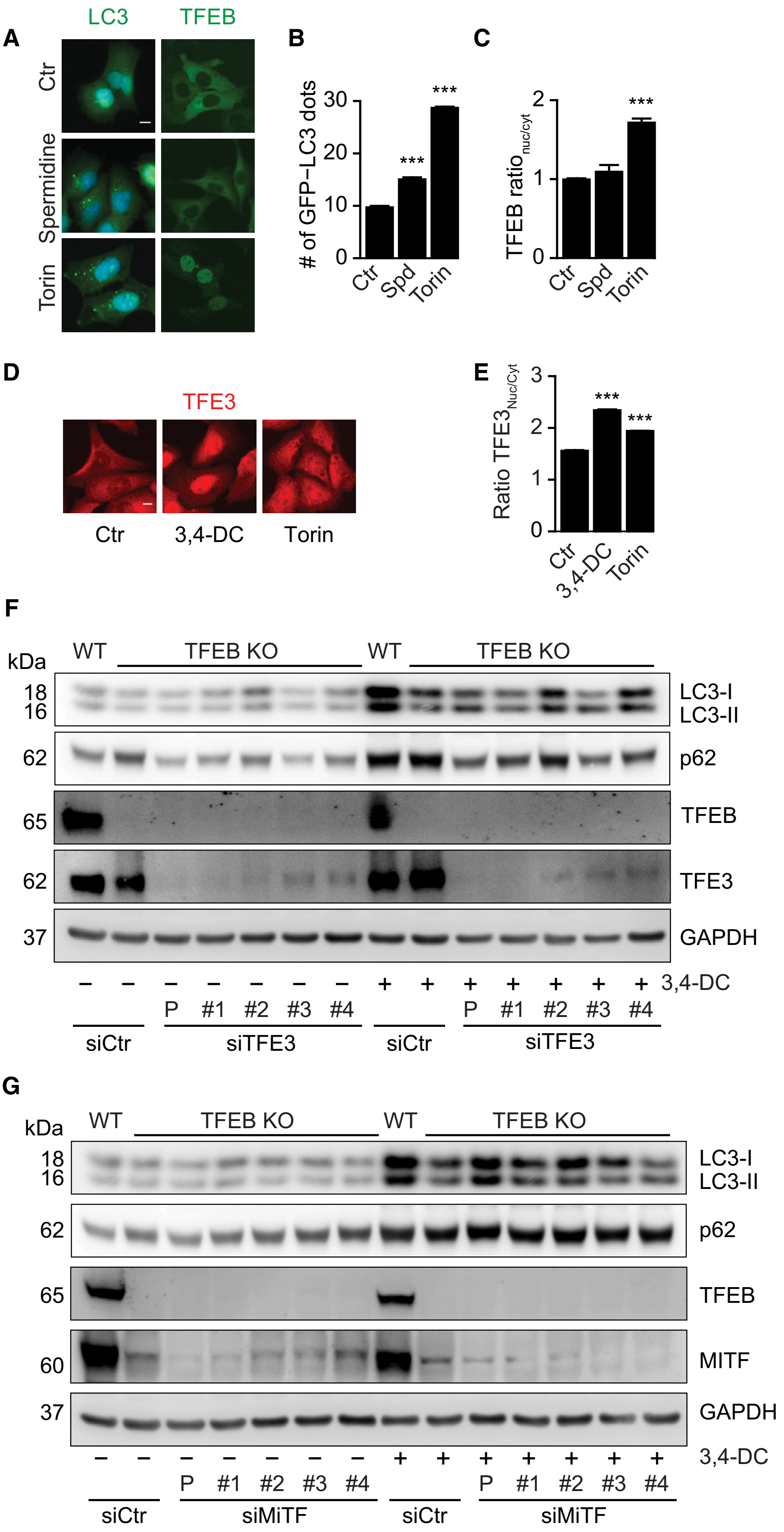

Figure EV3. Induction of p62 expression by 3,4-DC requires TFE3.

A–C U2OS cells stably expressing GFP-LC3 or TFEB-GFP were treated with spermidine or torin1 as indicated, and GFP-LC3 dot formation (B) and TFEB-GFP nuclear translocation (C) were quantified. Data are means ± SD of triplicate assessments (****P* < 0.001; Student's *t*-test). Representative images are shown in (A). Scale bar equals 10 µm.

D, E Endogenous TFE3 in U2OS cells was detected by immunofluorescence after 6 h of treatment with 3,4-DC or torin1. TFE3 intensity in nuclei and cytoplasm was measured, and their ratio was calculated in (E). Data are means ± SD of four replicates (****P* < 0.001; Student's *t*-test). Representative images are shown in (D). Scale bar equals 10 µm.

F, G U2OS wild-type or TFEB KO cells transfected with four individual siRNA oligos specifically targeting TFE3 (F) or MITF (G) (1#, 2#, 3#, and 4#) or a collection of the four individual siRNAs (s#), or non-targeting scramble siRNA (siCtr) were treated with or without 30 µM 3,4-DC for 16 h, as indicated. SDS–PAGE and immunoblot were performed to detect LC3, p62, and GAPDH protein levels. Samples for immunoblots were run on parallel gels, then blotted, cut into stripes, and probed separately.

Source data are available online for this figure.

© 2019 The Authors

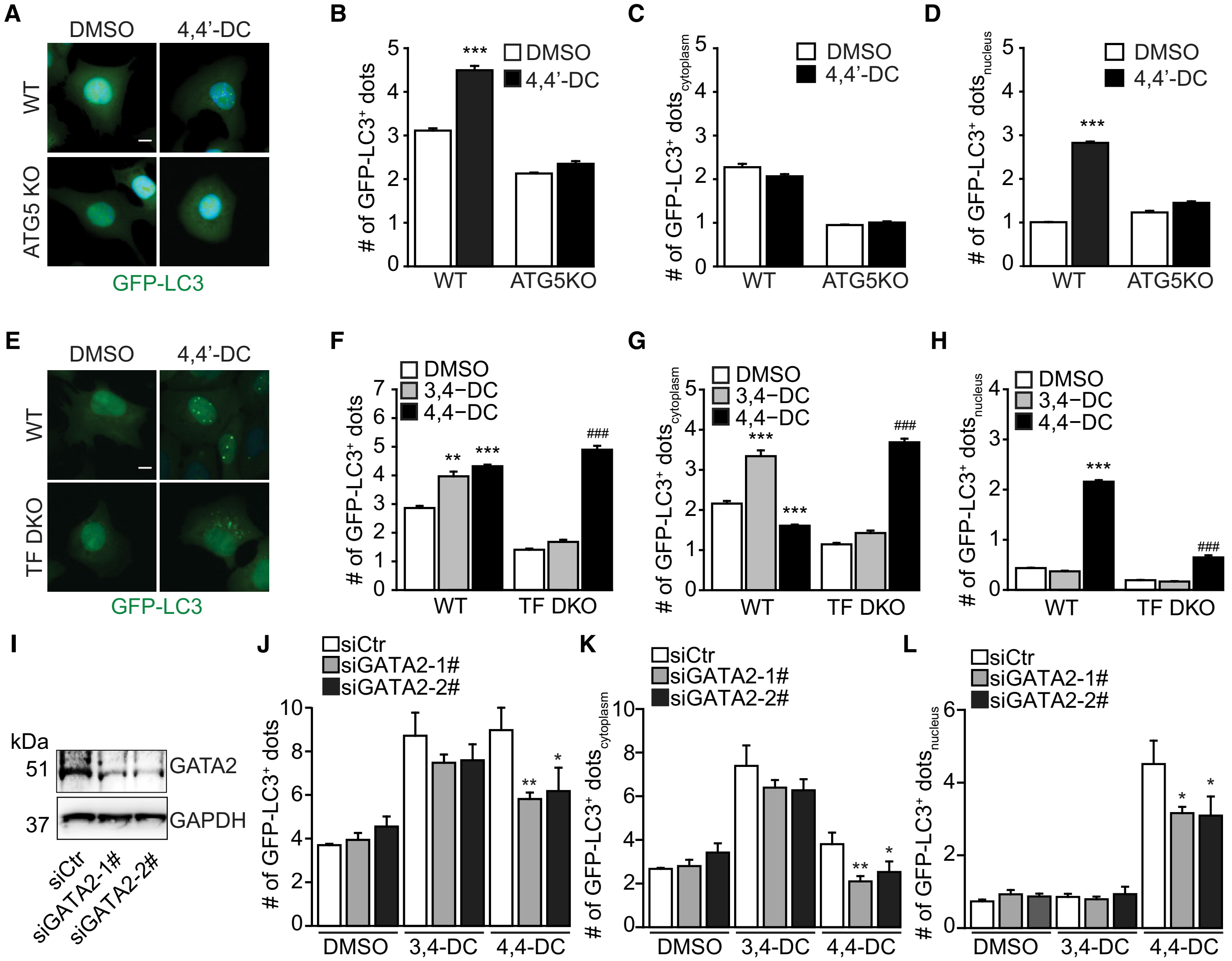

Figure EV4. Distinct modes of action of 3,4-DC and 4,4′-DC.

A–D U2OS wild-type (WT) and ATG5 knockout (ATG5 KO) cells stably expressing GFP-LC3 were treated with 4,4′-DC, and then, the cells were fixed and images were acquired by microscopy. Cytoplasmic (C), nuclear (D), and total (B) GFP-LC3 dots were counted. Data are means ± SD of three replicates (***$P < 0.001$ vs. WT/DMSO; Student's *t*-test). Representative images are shown in (A). Scale bar equals 10 μm.

E–H U2OS wild-type (WT) and TFEB and TFE3 double knockout (TF DKO) cells stably expressing GFP-LC3 were treated with 3,4-DC or 4,4′-DC, and then, the cells were fixed and images were acquired by microscopy. Cytoplasmic (G), nuclear (H), and total (F) GFP-LC3 dots were counted. Data are means ± SD of six replicates (**$P < 0.01$, ***$P < 0.001$ vs. WT/DMSO; ###$P < 0.001$ vs. TF DKO/DMSO; Student's *t*-test). Representative images are shown in (E). Scale bar equals 10 μm.

I–L U2OS-GFP-LC3 cells were transfected with two different siRNAs targeting GATA2 (siGATA2-1# and siGATA2-2#) or scramble siRNA as a control (siCtr), and then treated with 3,4-DC or 4,4′-DC. The cells were fixed, and images were acquired by microscopy. Cytoplasmic (K), nuclear (L), and total (J) GFP-LC3 dots were counted. Data are means ± SD of four replicates (*$P < 0.05$, **$P < 0.01$; Student's *t*-test). Samples for immunoblots in (I) were run in parallel instances and probed separately.

Source data are available online for this figure.

 © 2019 The Authors

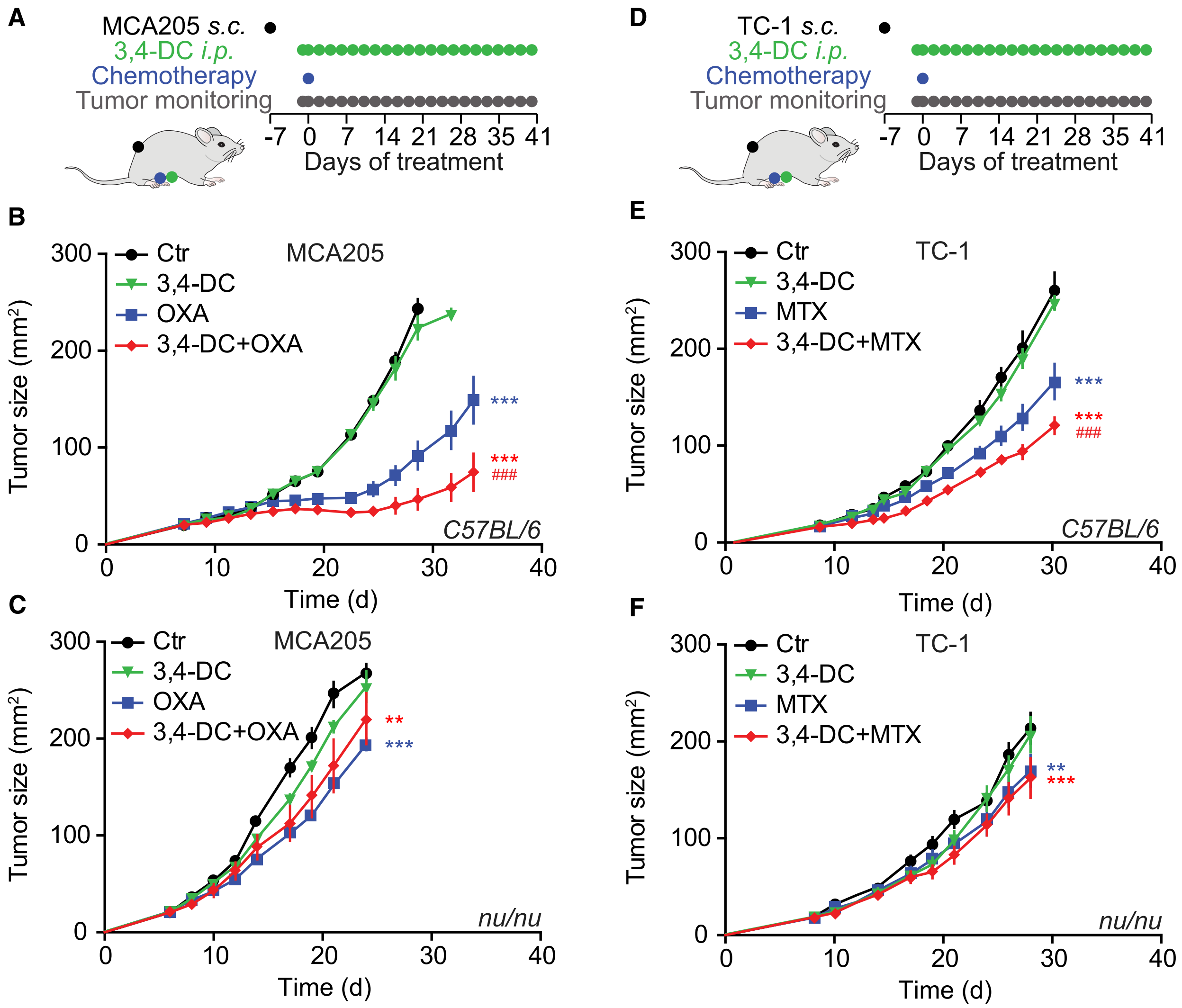

Figure EV5. 3,4-DC reduces tumor growth in several models, in combination with two different chemotherapeutics.

A Schematic overview of the treatment of MCA205 fibrosarcomas with oxaliplatin (OXA) and 3,4-DC, alone, or in combination.

B, C Growth kinetic of MCA205 fibrosarcomas that were evolving in immunocompetent C57Bl/6 mice (B) or immunodeficient *nu/nu* mice (C), treated as indicated in (A). Asterisks indicate significant effects of OXA with respect to untreated controls (mean value ± SEM, $^{**}P < 0.01$, $^{***}P < 0.001$; Student's *t*-test), while hash symbols refer to the comparison of the effects of OXA plus 3,4-DC to OXA alone ($^{\#\#\#}P < 0.001$; Student's *t*-test) ($n = 6$–12).

D Schematic overview of the treatment of TC-1 lung cancers with mitoxantrone (MTX) and 3,4-DC, alone or in combination.

E, F Growth kinetic of TC-1 lung cancers that were evolving in immunocompetent C57Bl/6 mice (E) or immunodeficient *nu/nu* mice (F), treated as indicated in (D). Asterisks indicate significant effects of MTX with respect to untreated controls (mean value ± SEM, $^{**}P < 0.01$, $^{***}P < 0.001$; Student's *t*-test), while hash symbols refer to the comparison of the effects of OXA and MTX plus 3,4-DC to OXA or MTX alone ($^{\#\#\#}P < 0.001$; Student's *t*-test) ($n = 6$–12).

© 2019 The Authors
