# Supplementary figures and images for: 3,4‐Dimethoxychalcone induces autophagy through activation of the transcription factors TFE3 and TFEB
Source: EMBO Mol Med. 2019 Oct 14;11(11):e10469. doi: 10.15252/emmm.201910469 (PMC6835206; doi:10.15252/emmm.201910469)

EV 1A

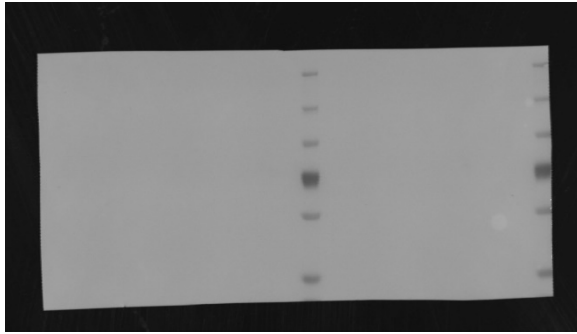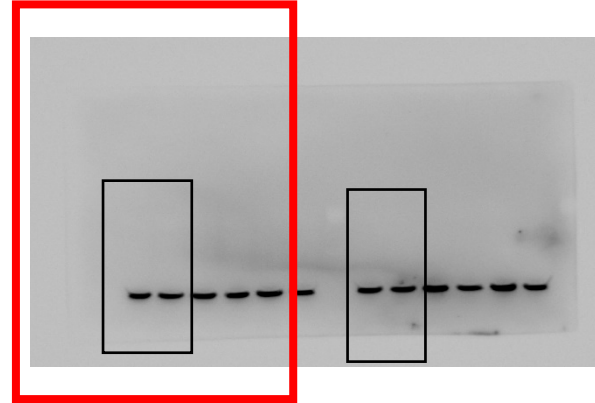

GAPDH

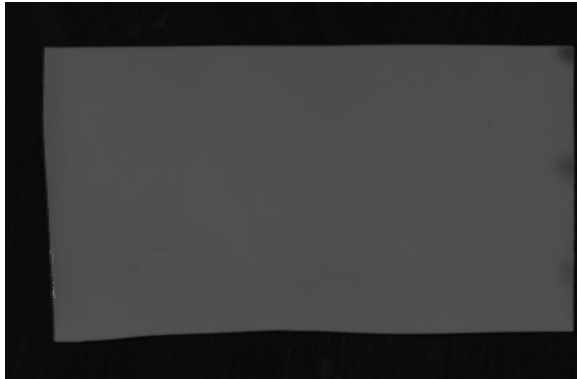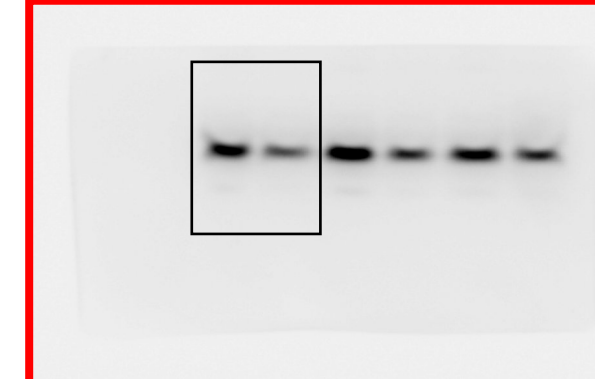

Ace-H2A-K5

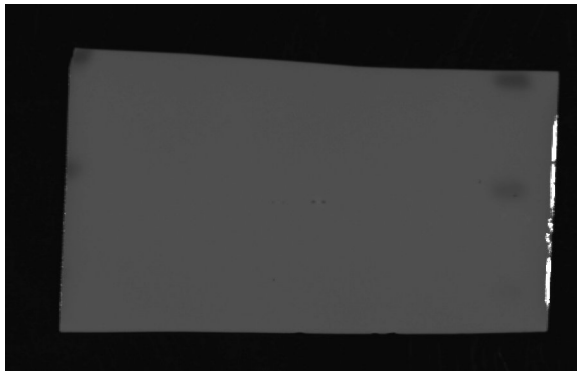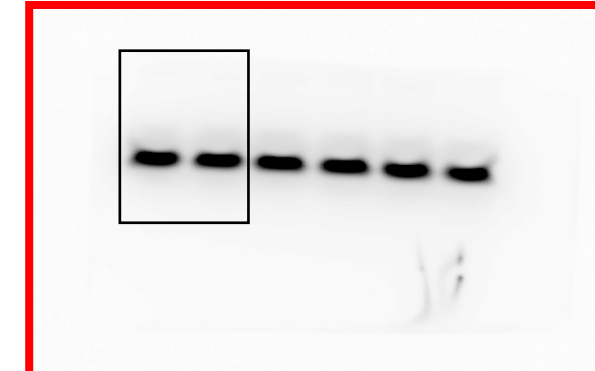

H2A

EV 1C

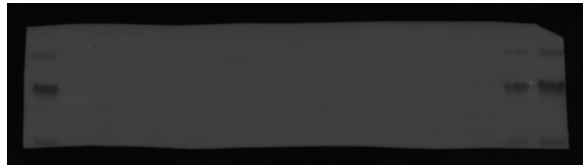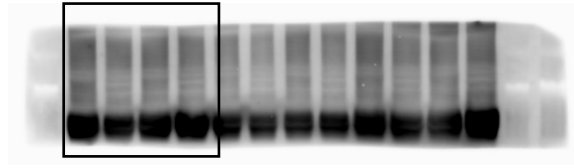

HA

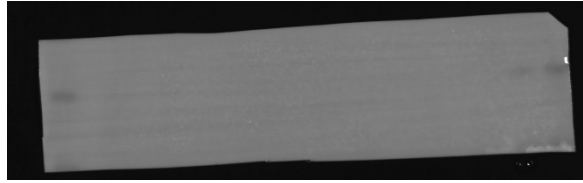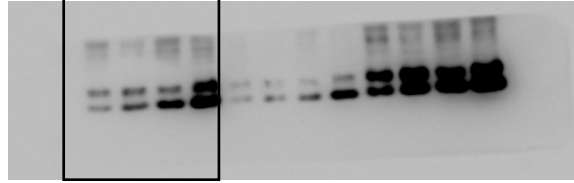

LC3

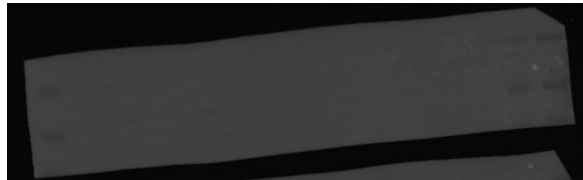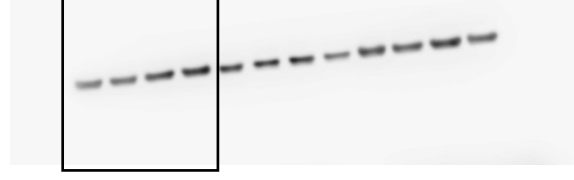

GAPDH

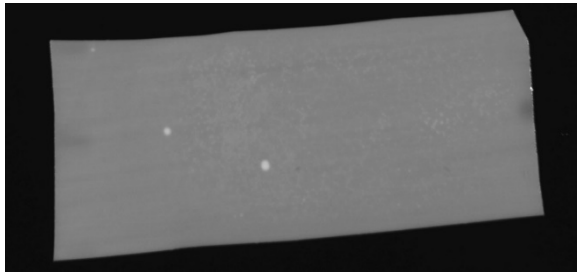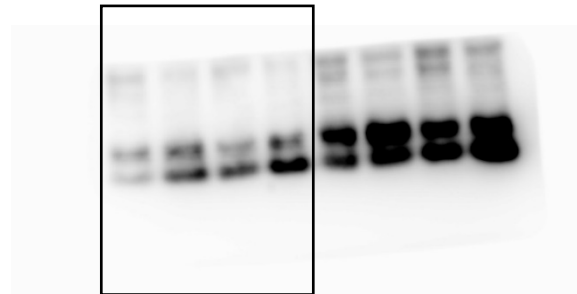

LC3

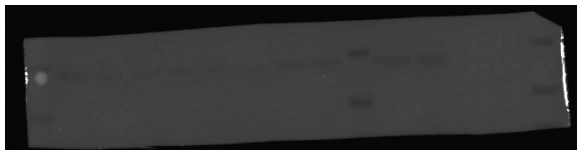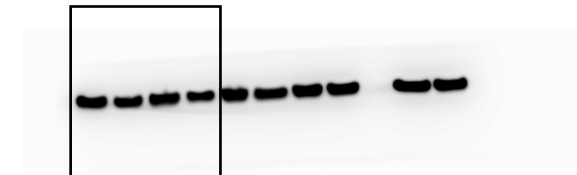

GAPDH

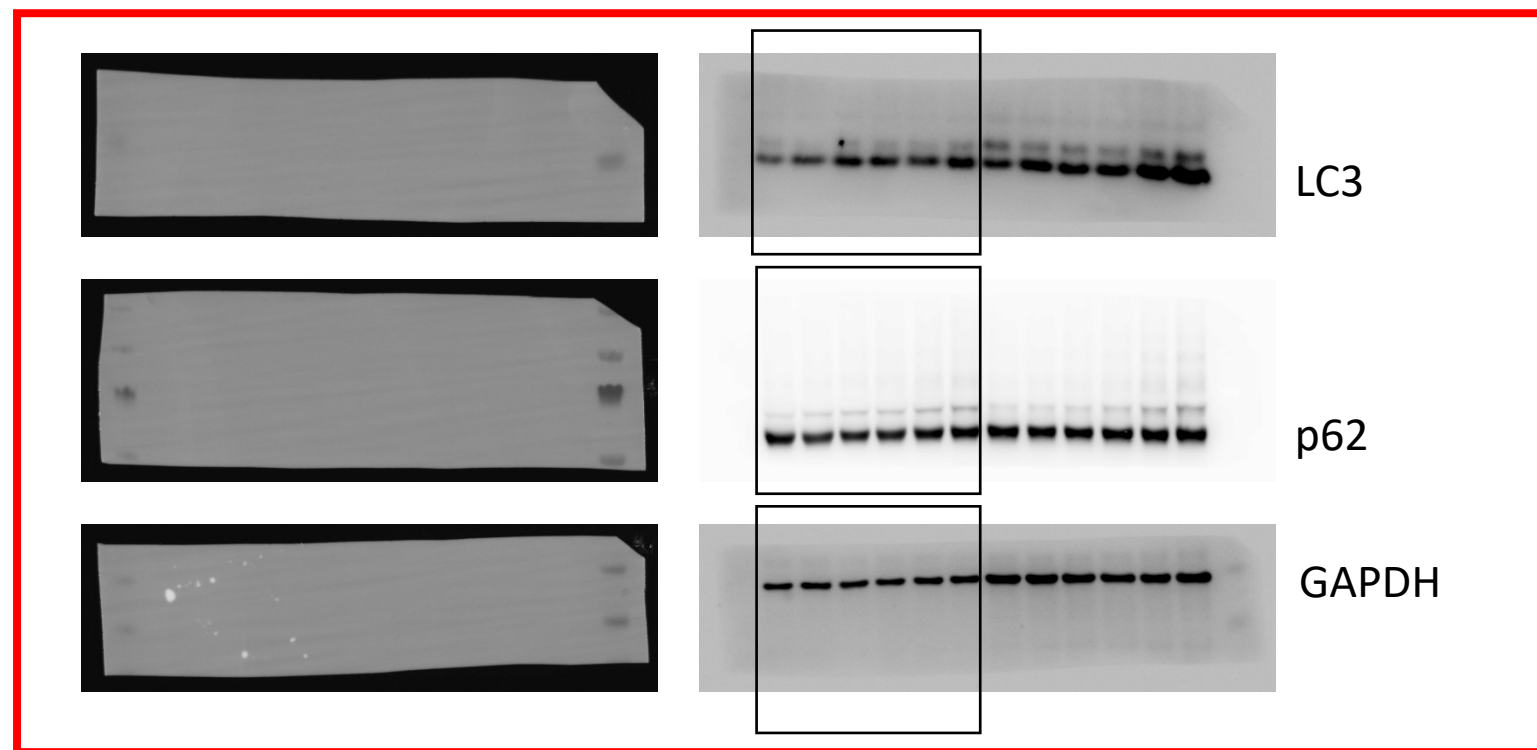

EV1G

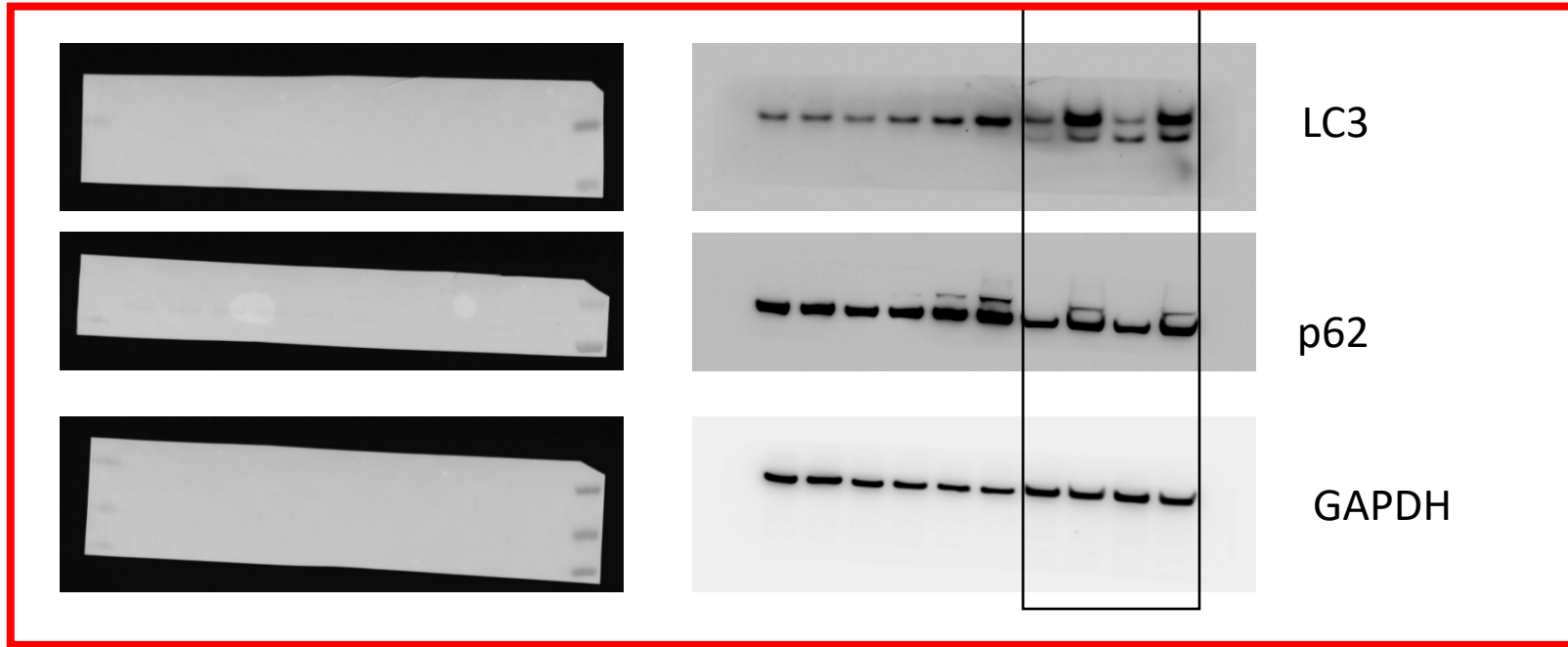

EV1H

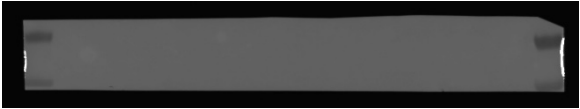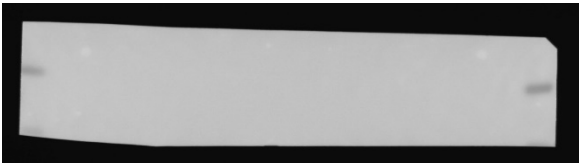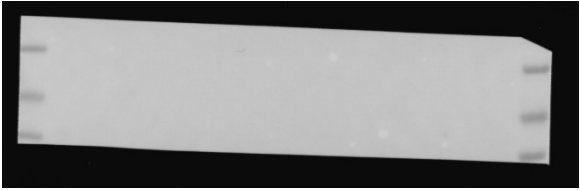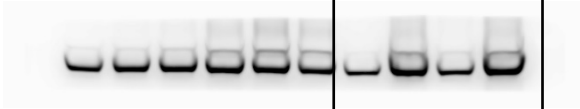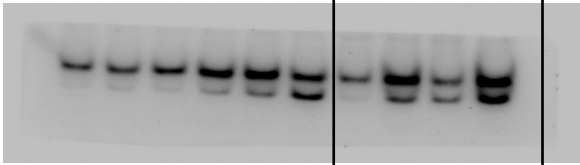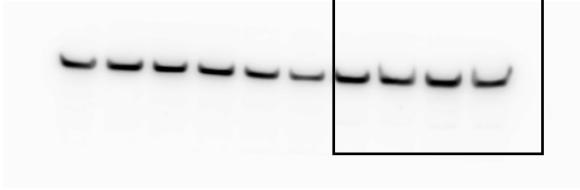

p62

LC3

GAPDH

Supplement: Supplementary file 3 — Source Data for Expanded View and Appendix [file EMMM-11-e10469-s009.zip › EMM-10469_EV_Appendix_source_data/EMM-10469-source-data-figev1.pdf]

EV 3F

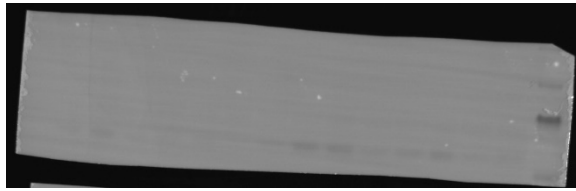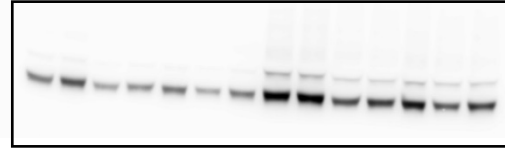

p62

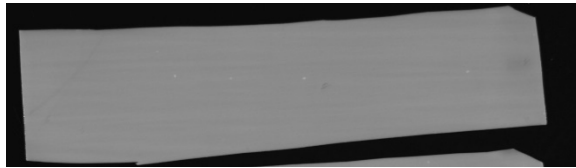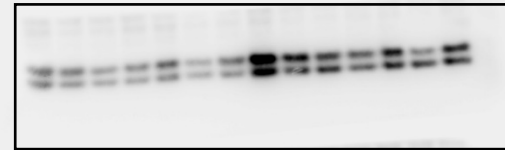

LC3

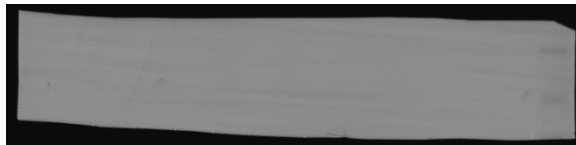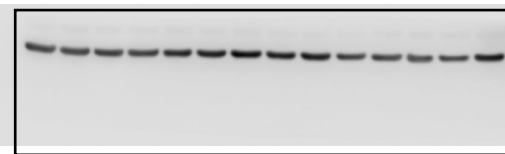

GAPDH

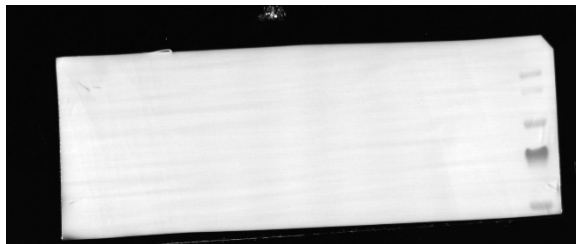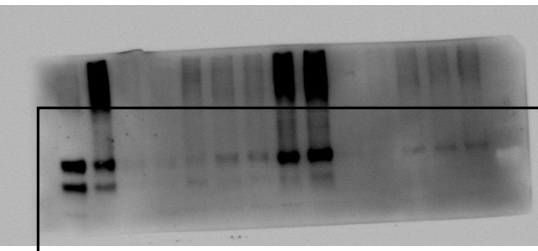

TFE3

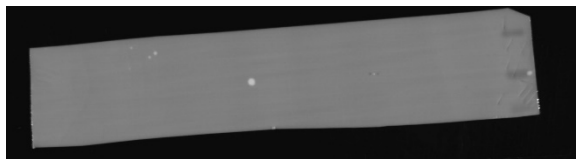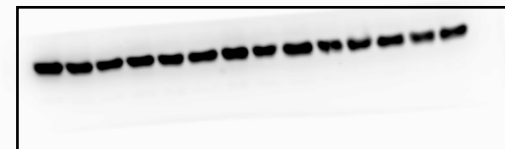

GAPDH

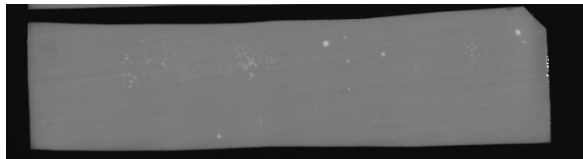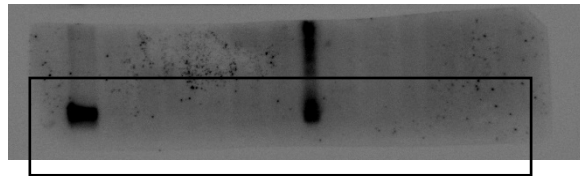

TFEB

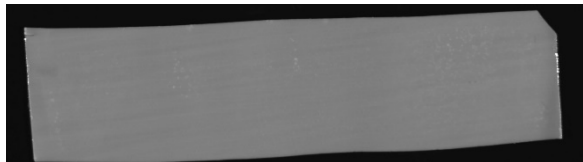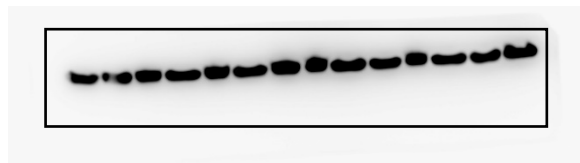

GAPDH

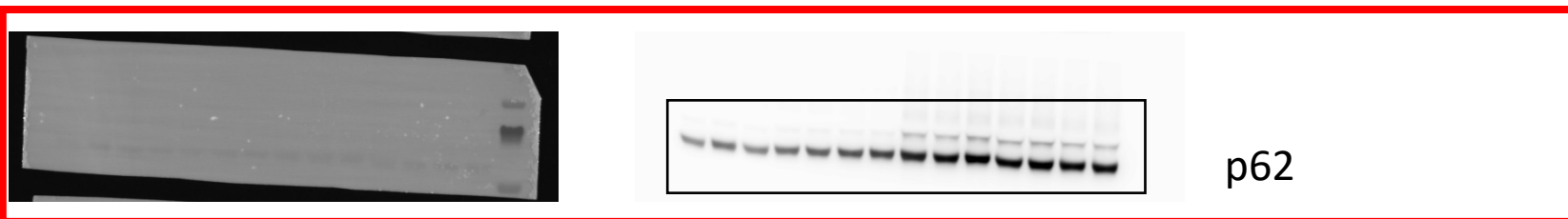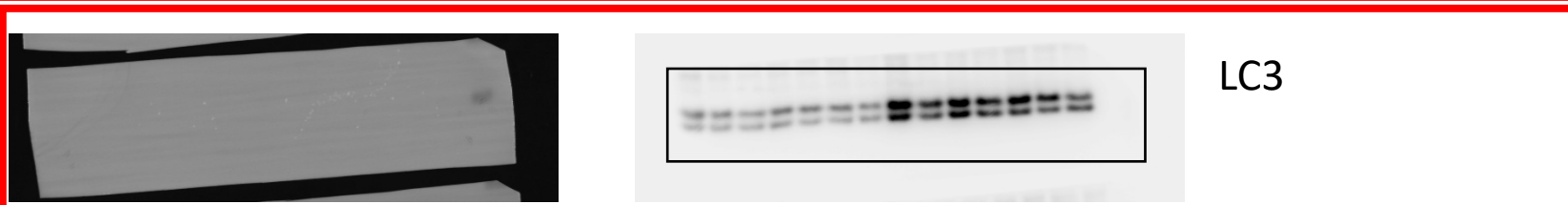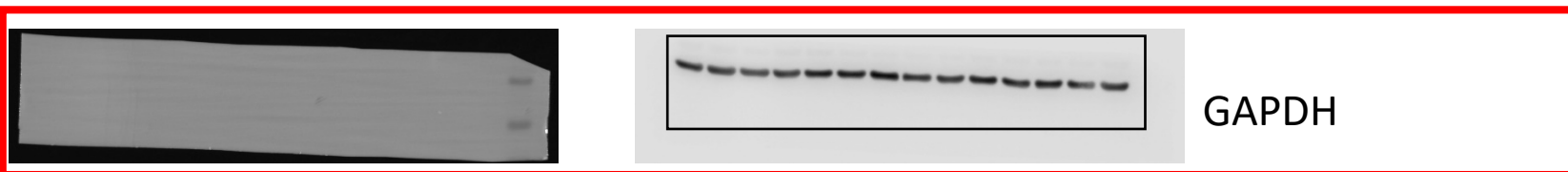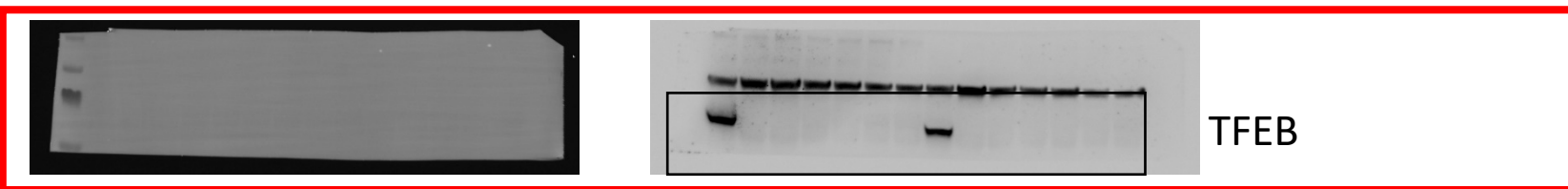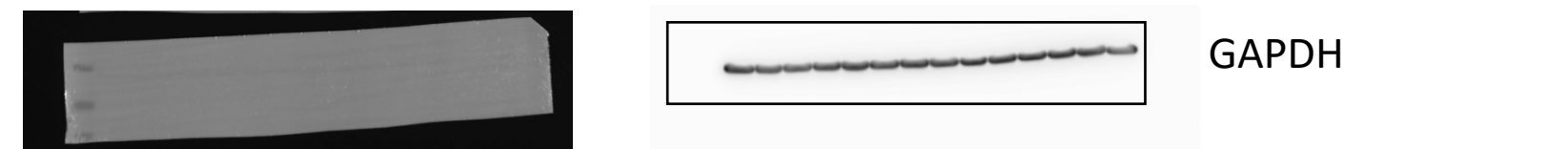

EV 3G

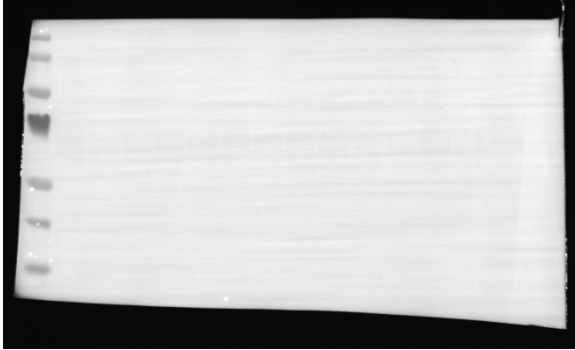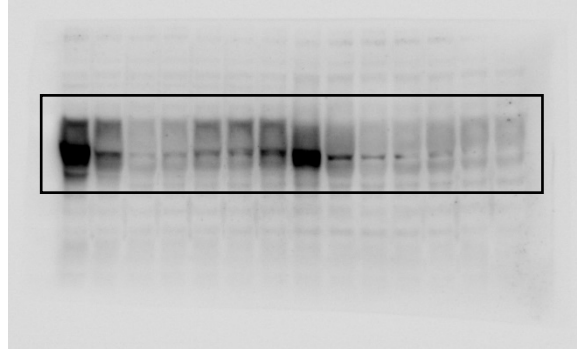

MiTF

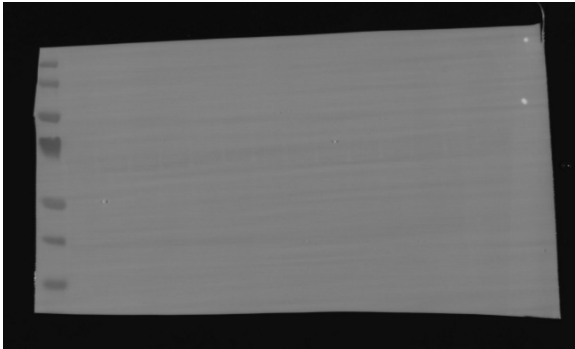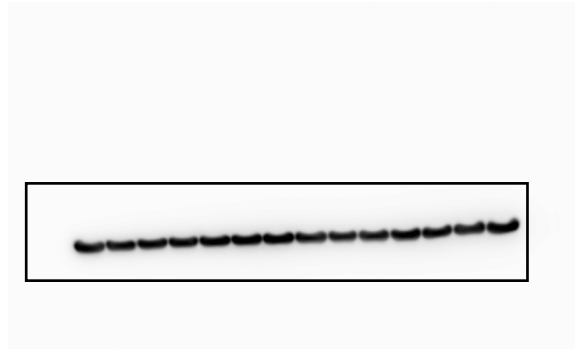

GAPDH

Supplement: Supplementary file 3 — Source Data for Expanded View and Appendix [file EMMM-11-e10469-s009.zip › EMM-10469_EV_Appendix_source_data/EMM-10469-source-data-figev3.pdf]

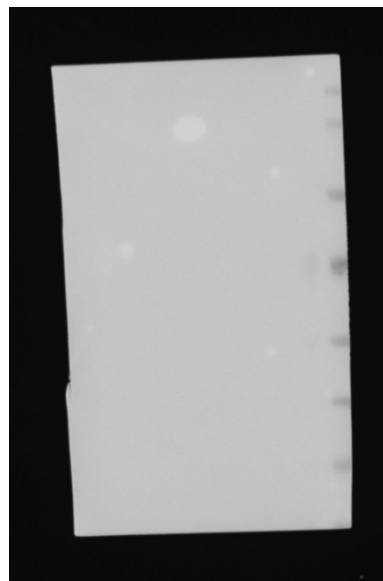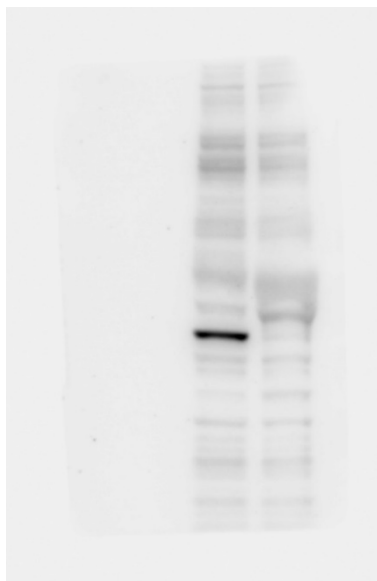

ATG5

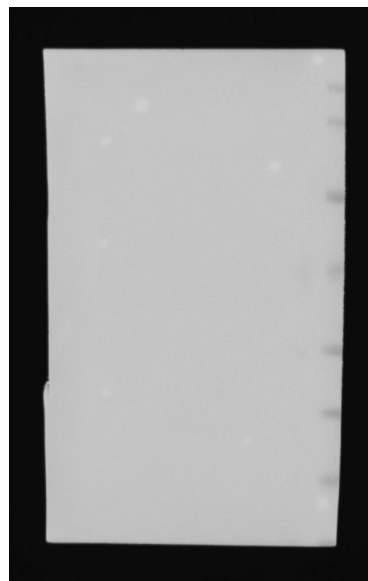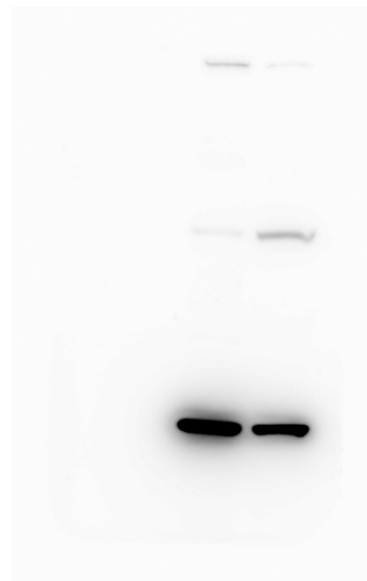

GAPDH

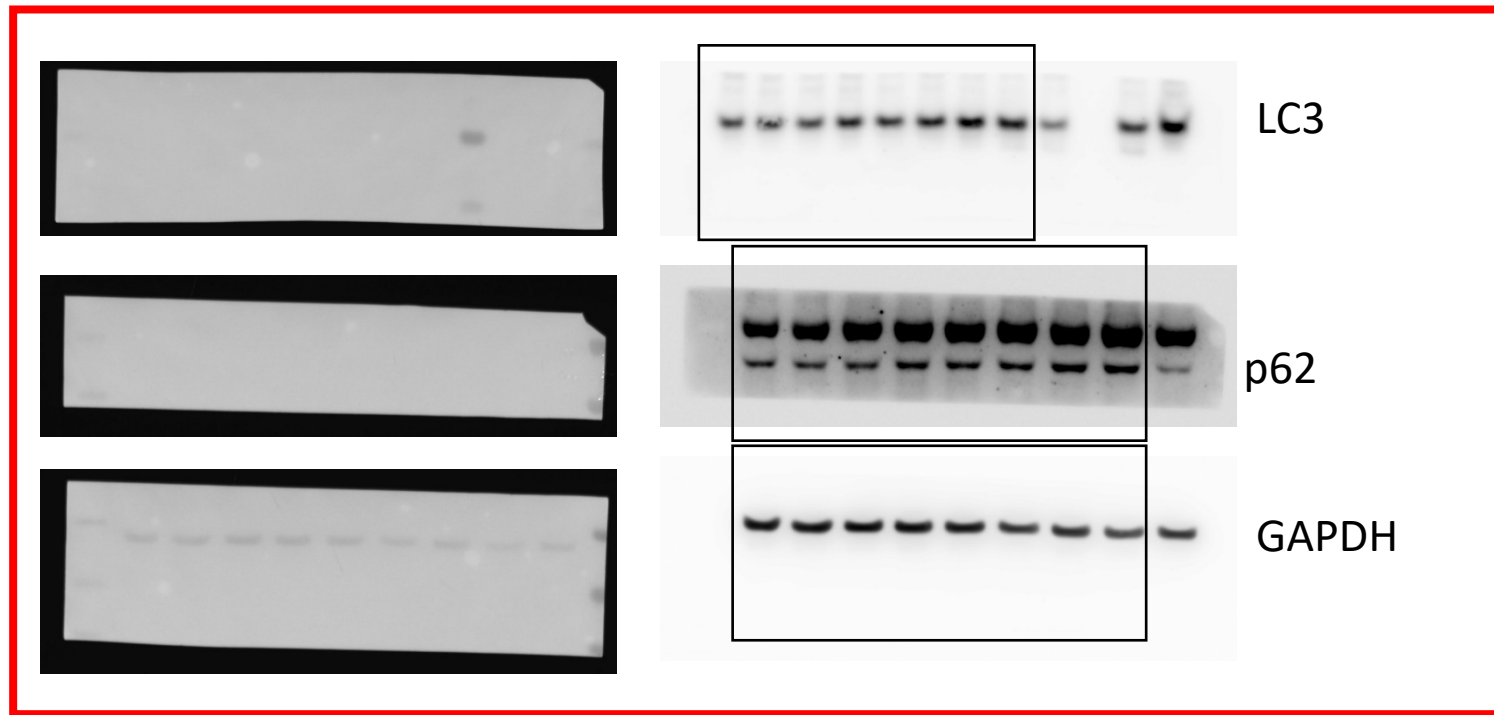

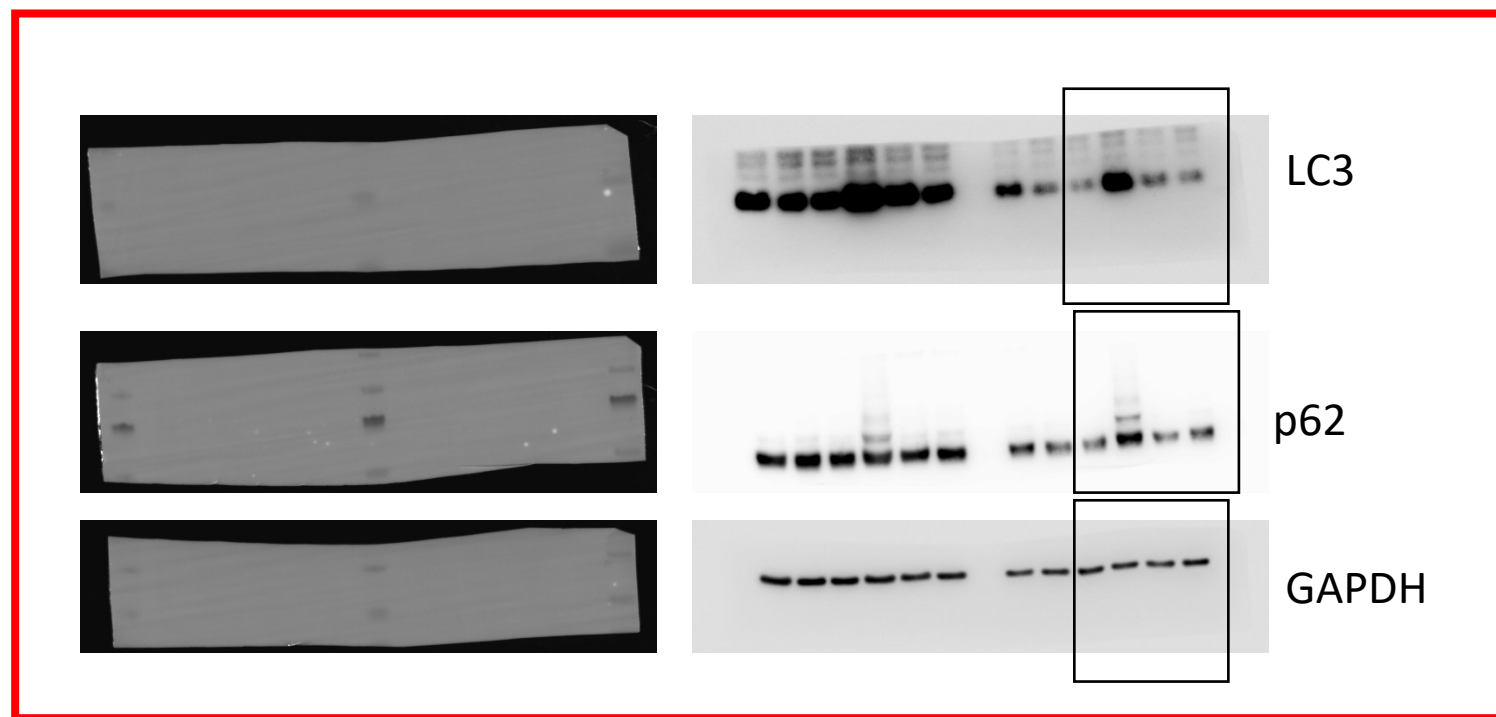

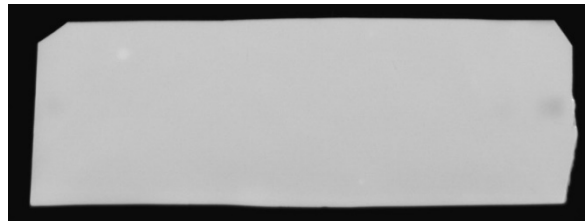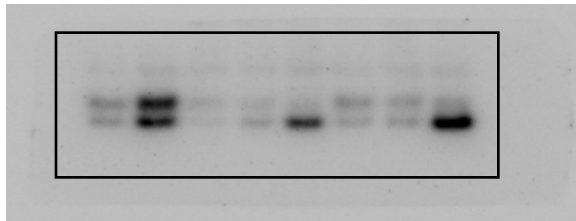

LC3

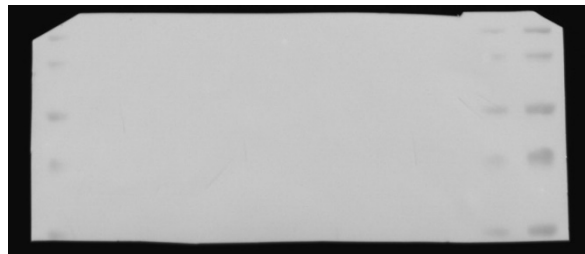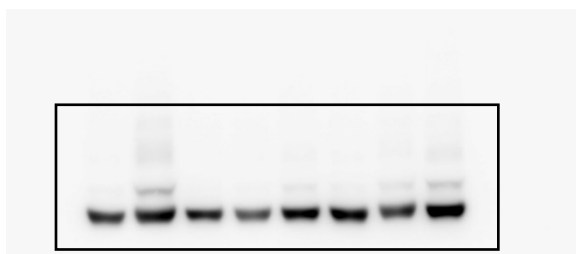

p62

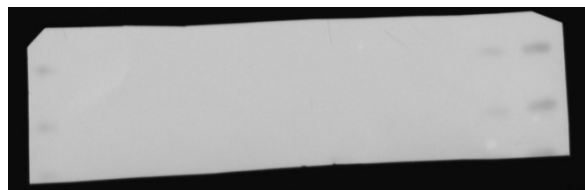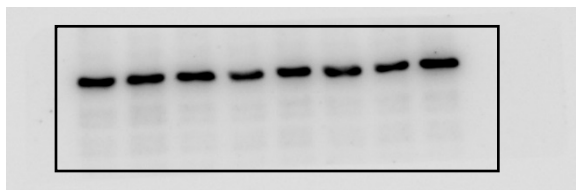

GAPDH

EV 2E

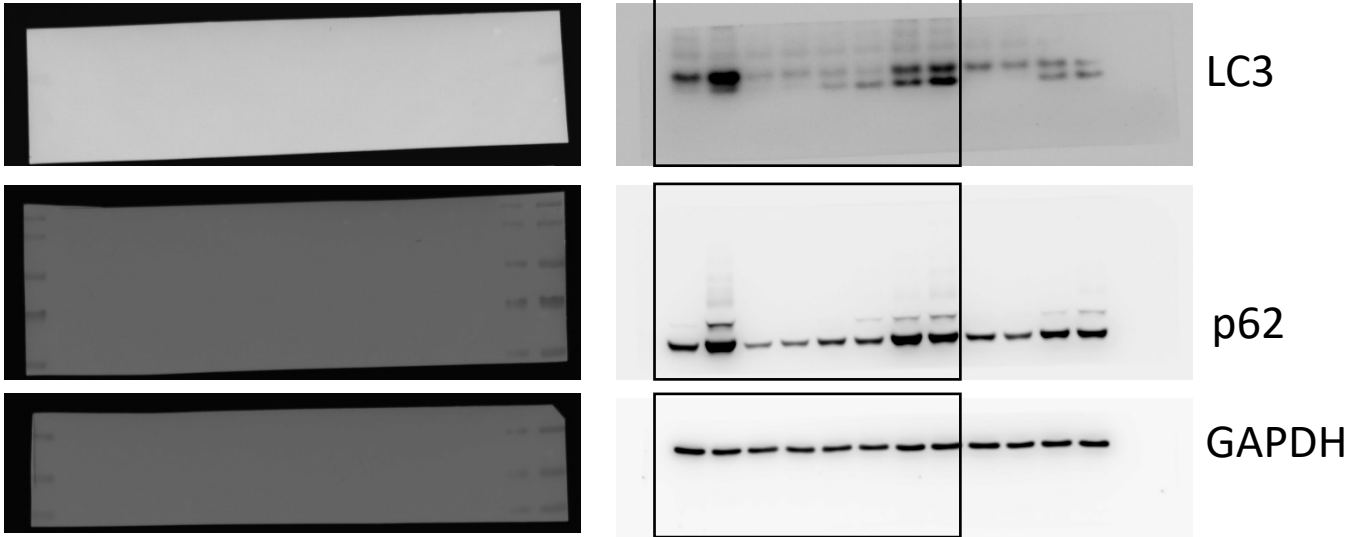

Supplement: Supplementary file 3 — Source Data for Expanded View and Appendix [file EMMM-11-e10469-s009.zip › EMM-10469_EV_Appendix_source_data/EMM-10469-source-data-figev2.pdf]

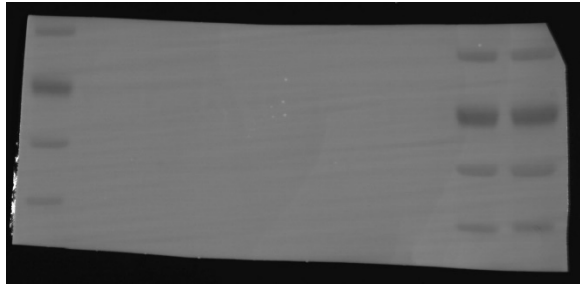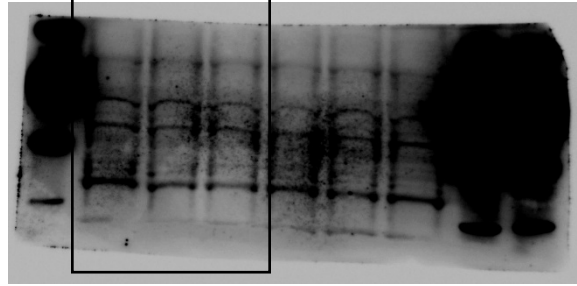

GATA2

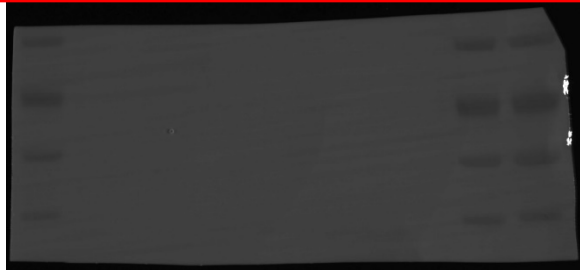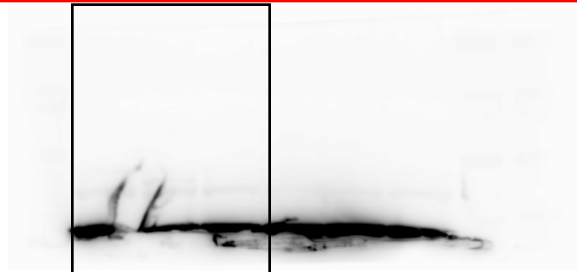

GAPDH

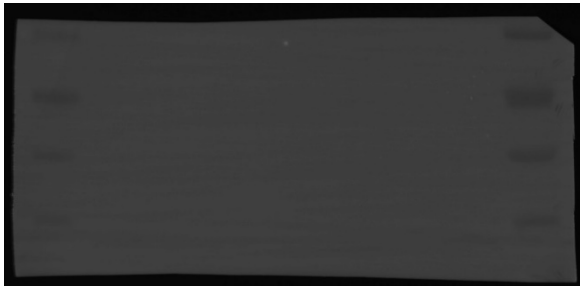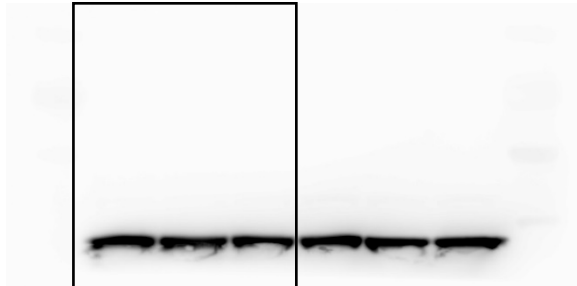

GAPDH

Supplement: Supplementary file 3 — Source Data for Expanded View and Appendix [file EMMM-11-e10469-s009.zip › EMM-10469_EV_Appendix_source_data/EMM-10469-source-data-figev4.pdf]

Figure S3A

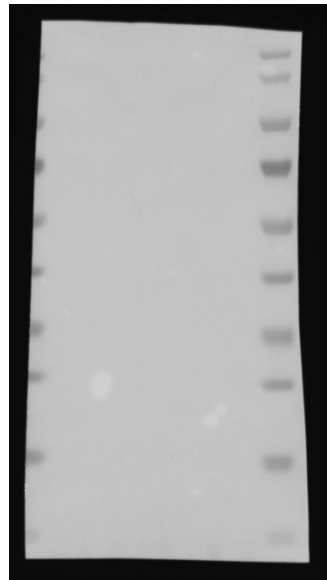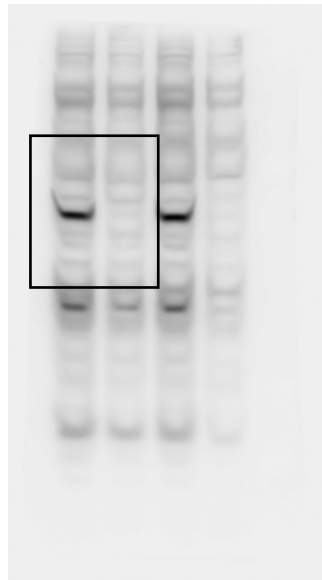

ATG5

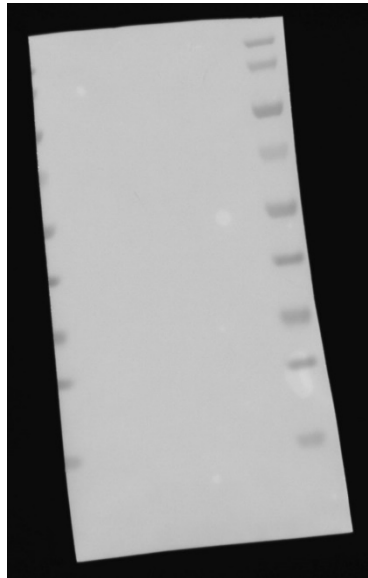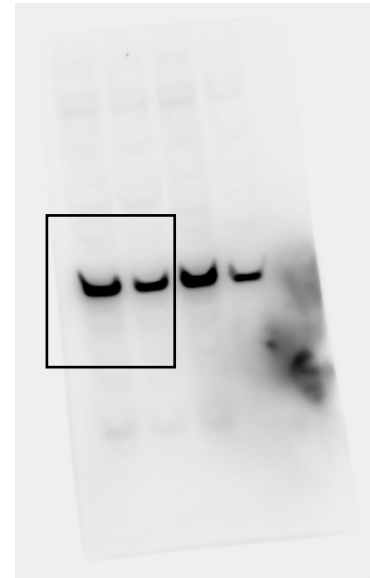

GAPDH

Figure S3B

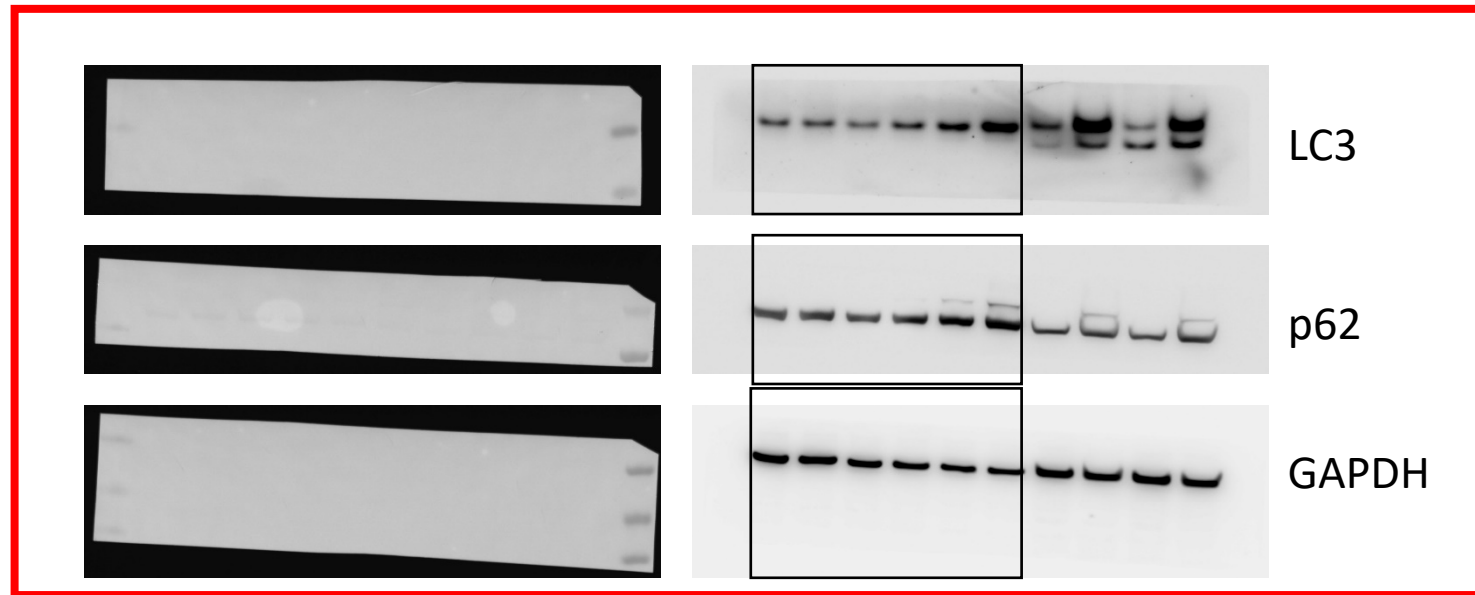

Figure S3C

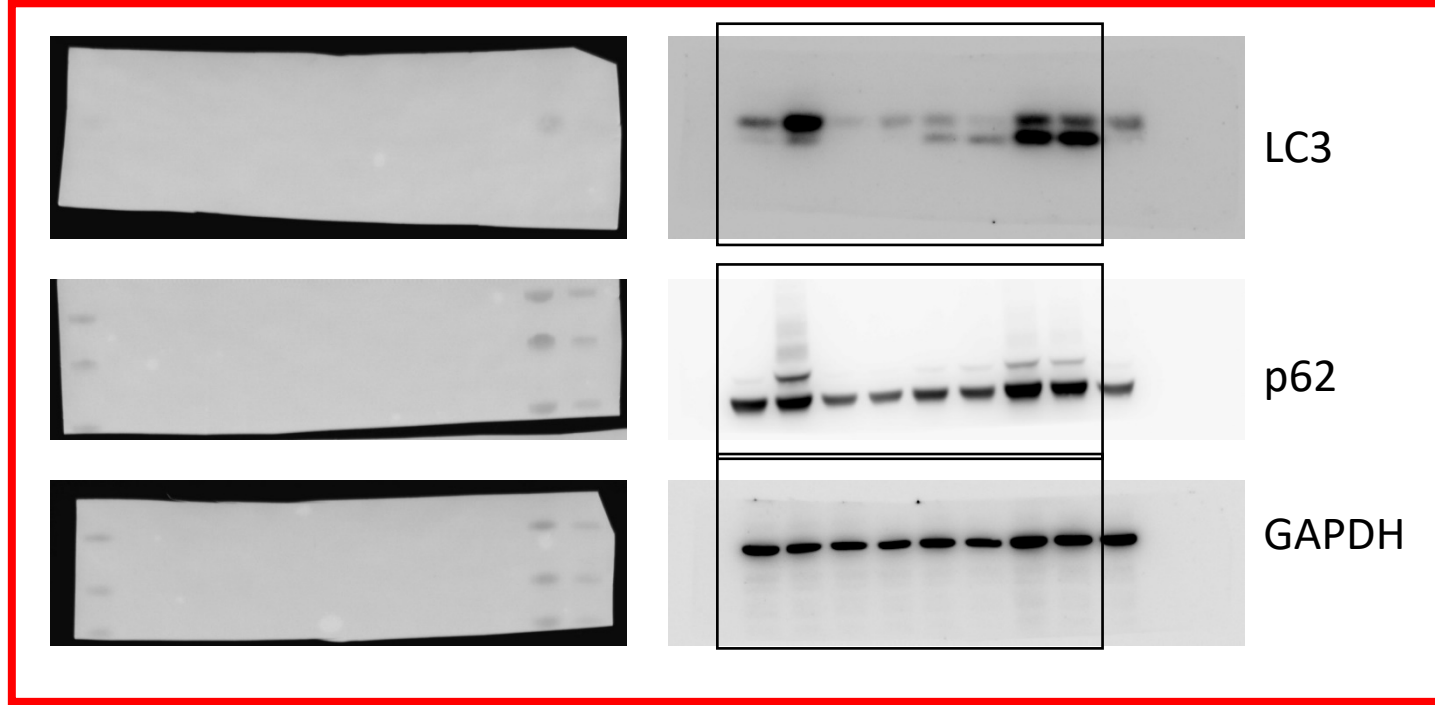

Supplement: Supplementary file 3 — Source Data for Expanded View and Appendix [file EMMM-11-e10469-s009.zip › EMM-10469_EV_Appendix_source_data/EMM-10469-source-data-figs3.pdf]

Figure S4B

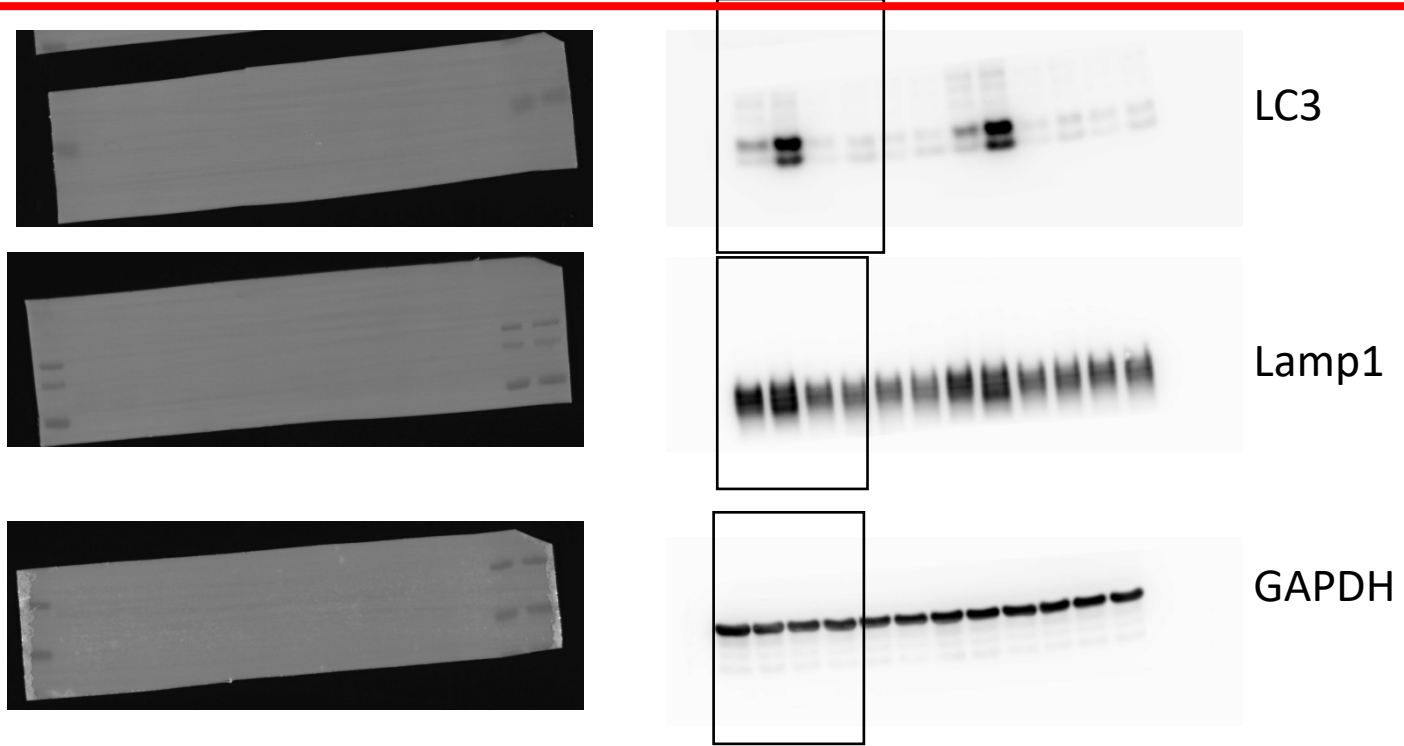

Figure S4B

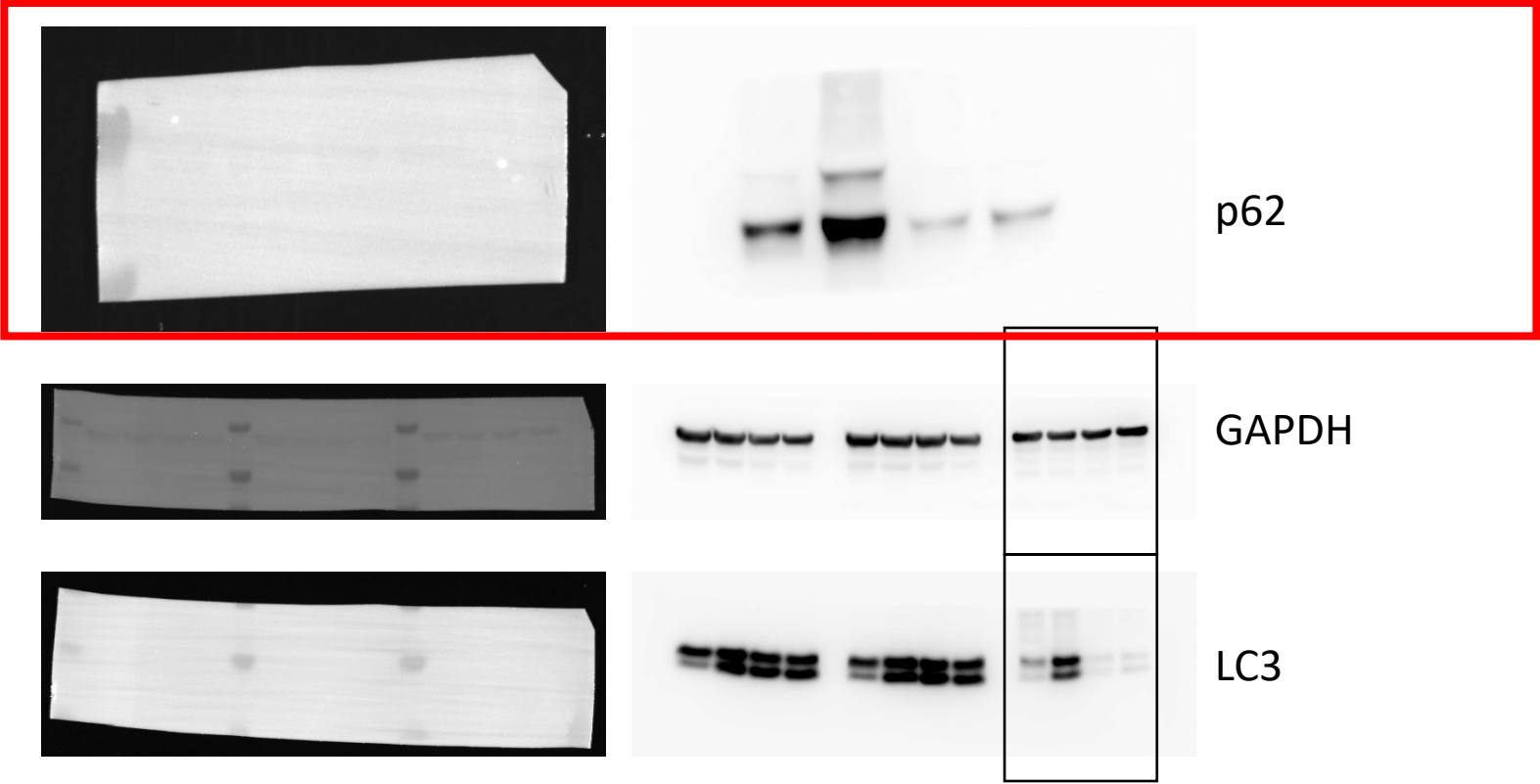

Supplement: Supplementary file 3 — Source Data for Expanded View and Appendix [file EMMM-11-e10469-s009.zip › EMM-10469_EV_Appendix_source_data/EMM-10469-source-data-figs4.pdf]

Figure 3A

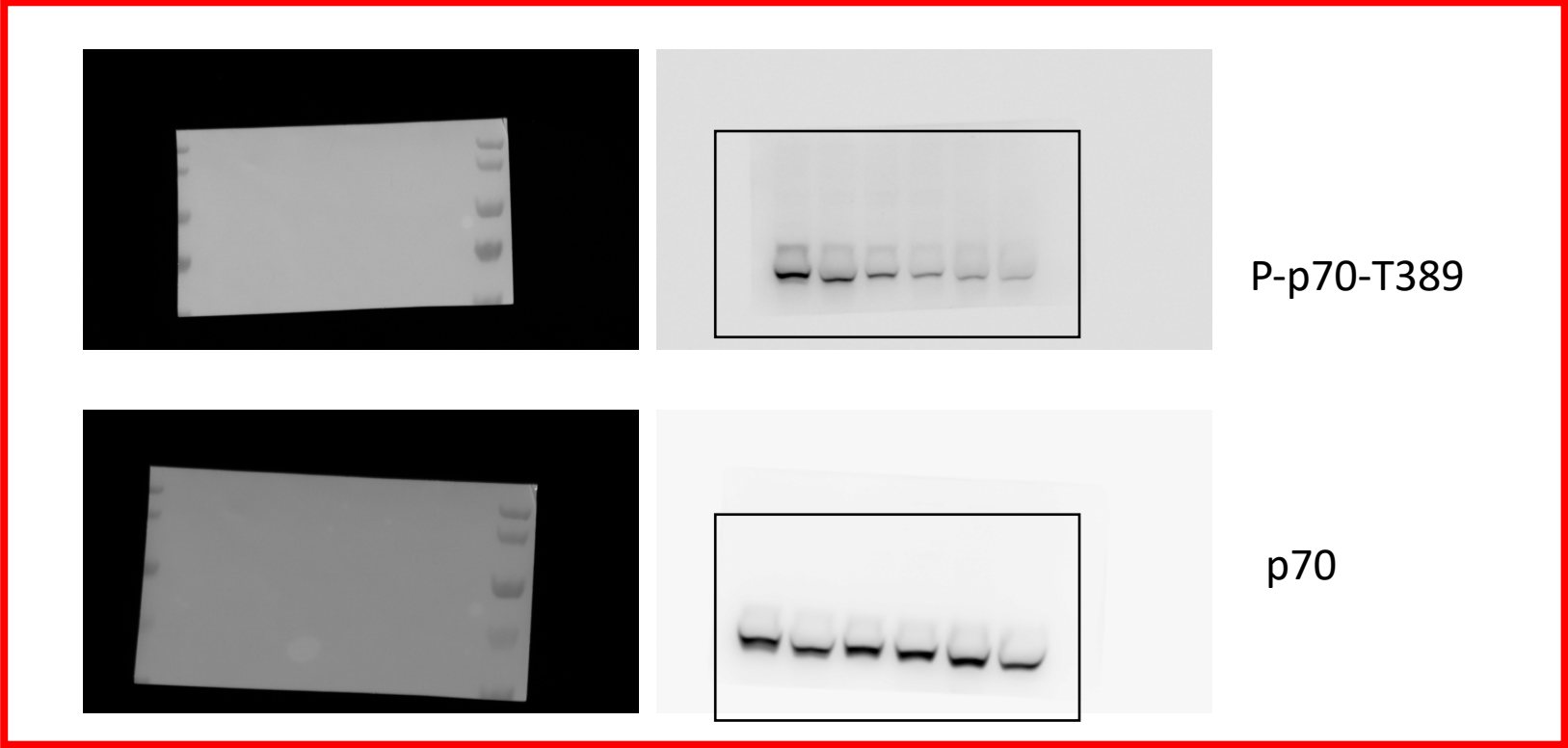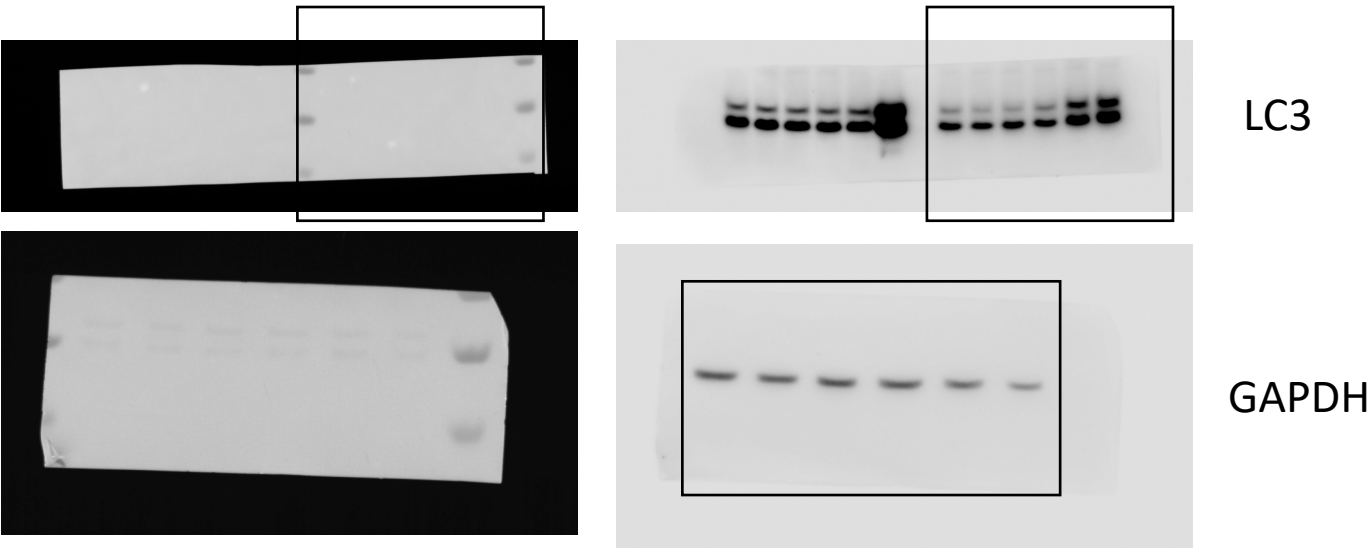

Figure 3A

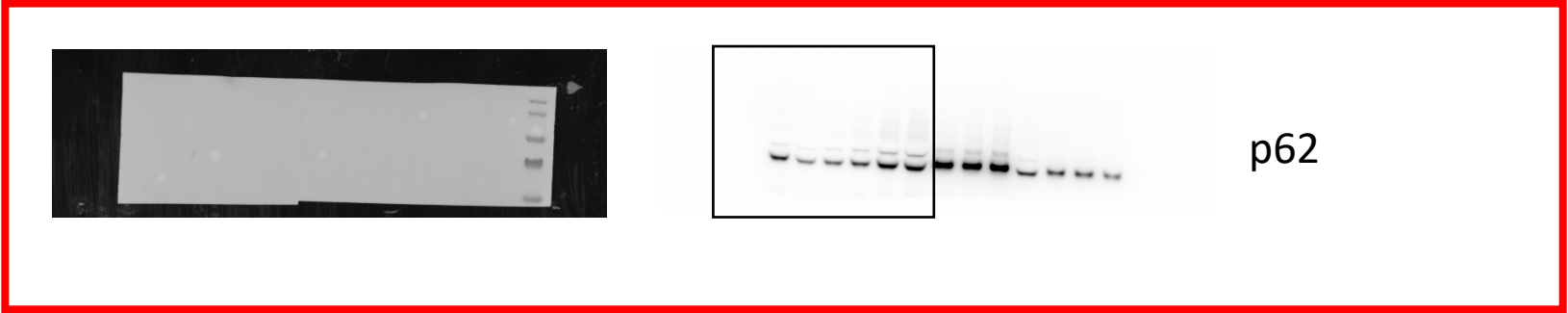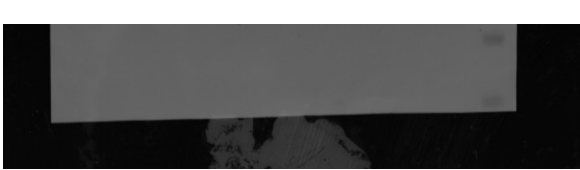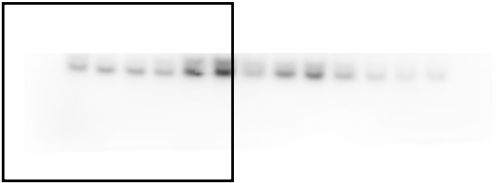

LC3

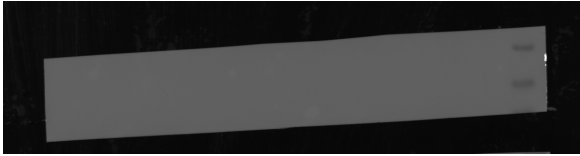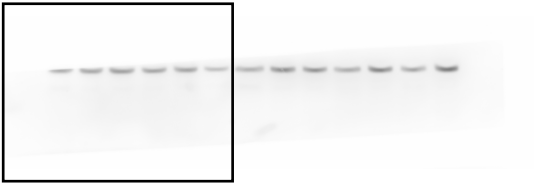

GAPDH

Figure 3A

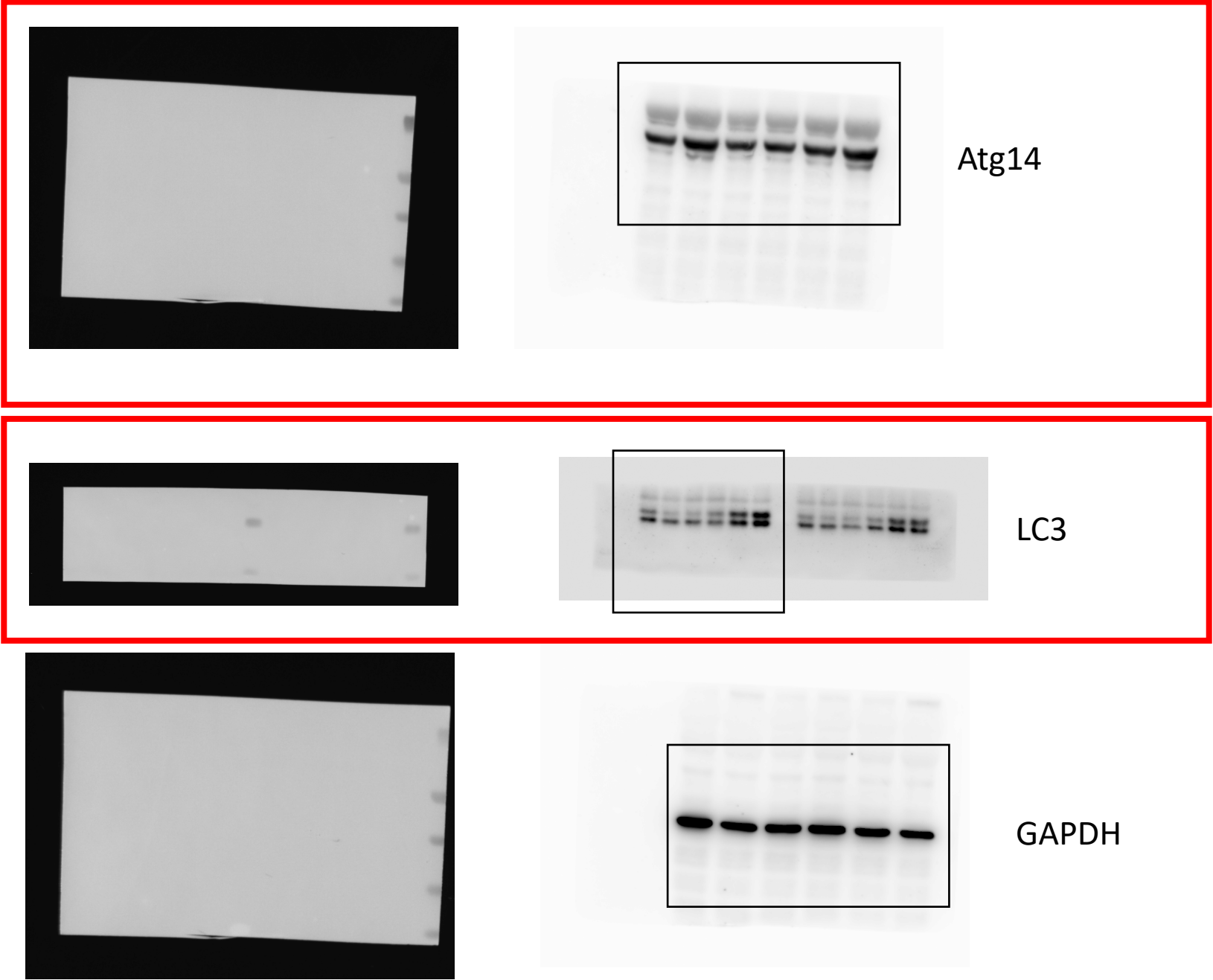

Figure 3A

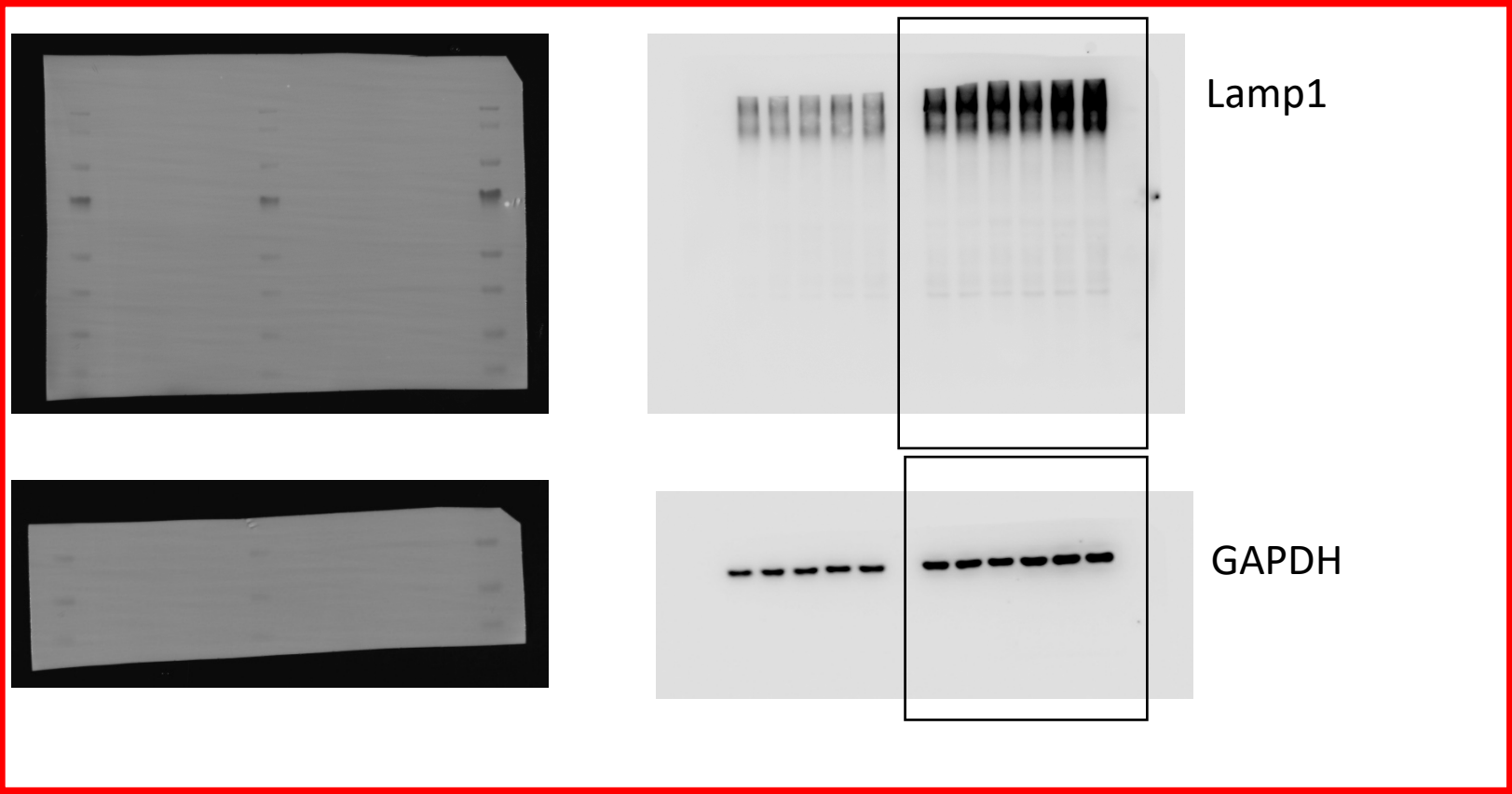

Figure 3B

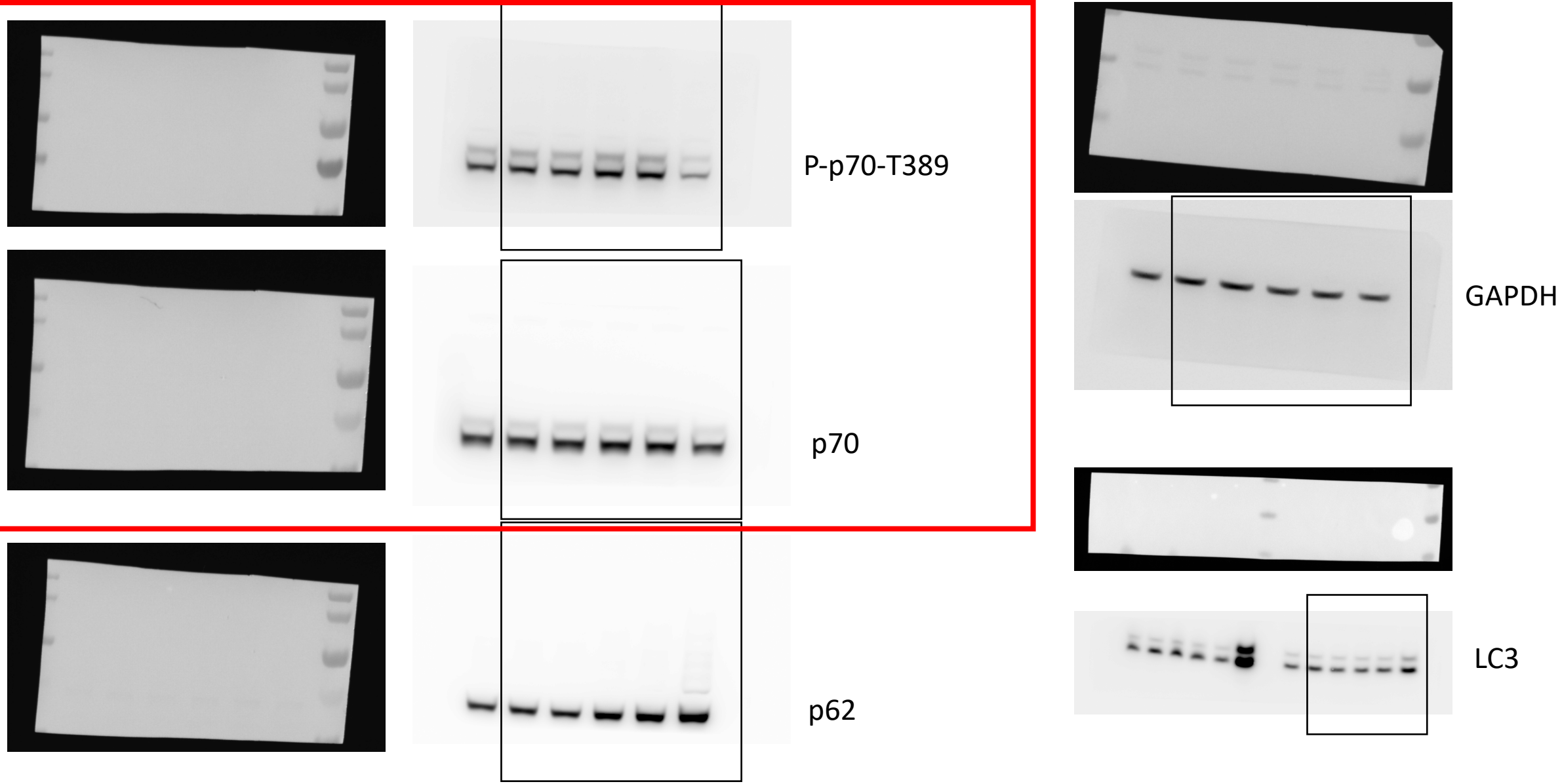

Figure 3B

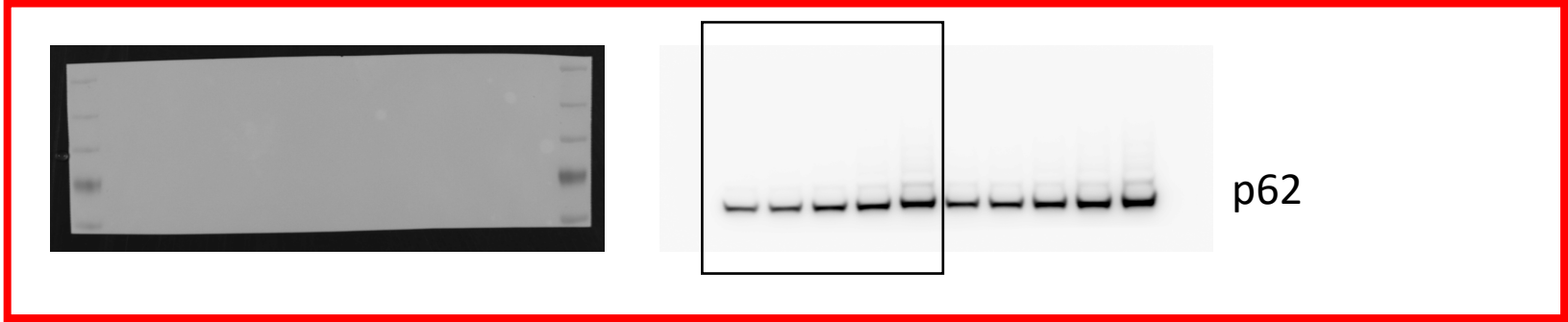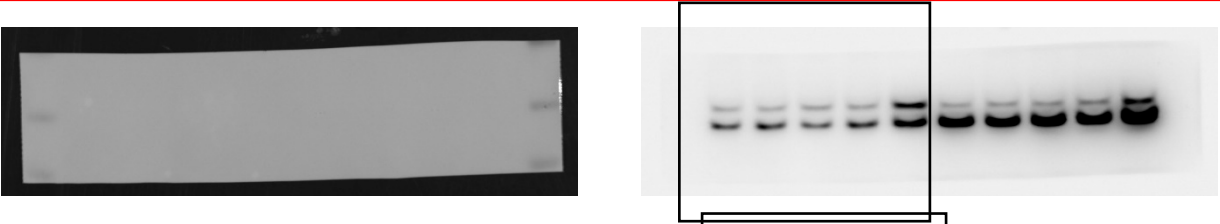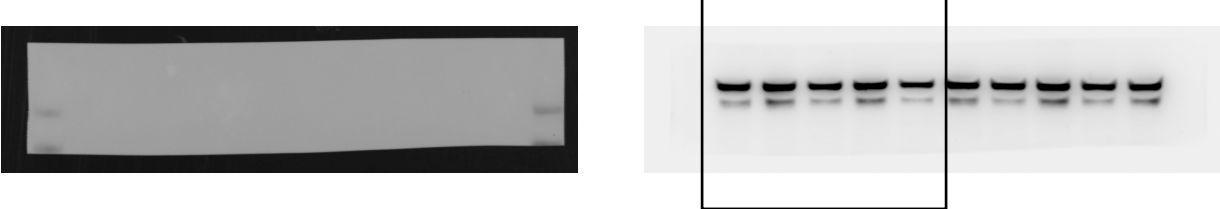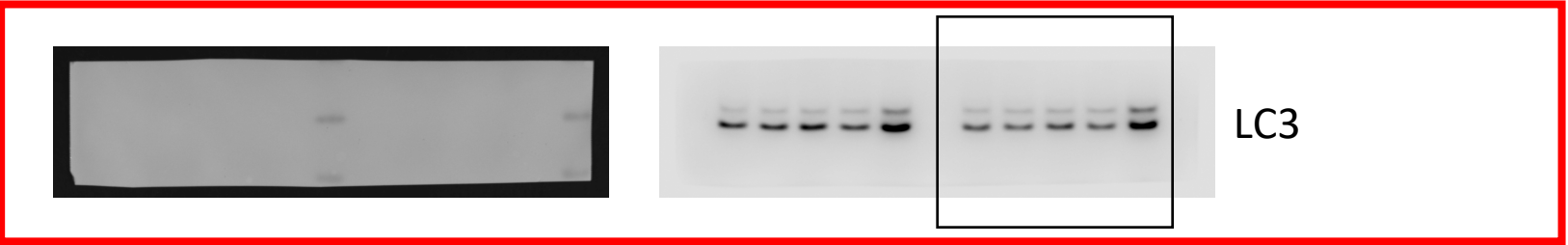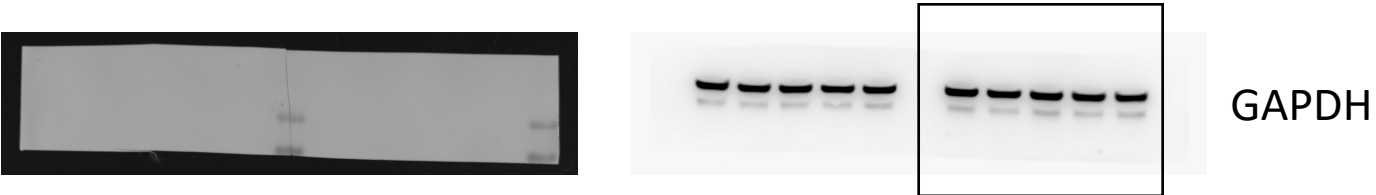

Figure 3B

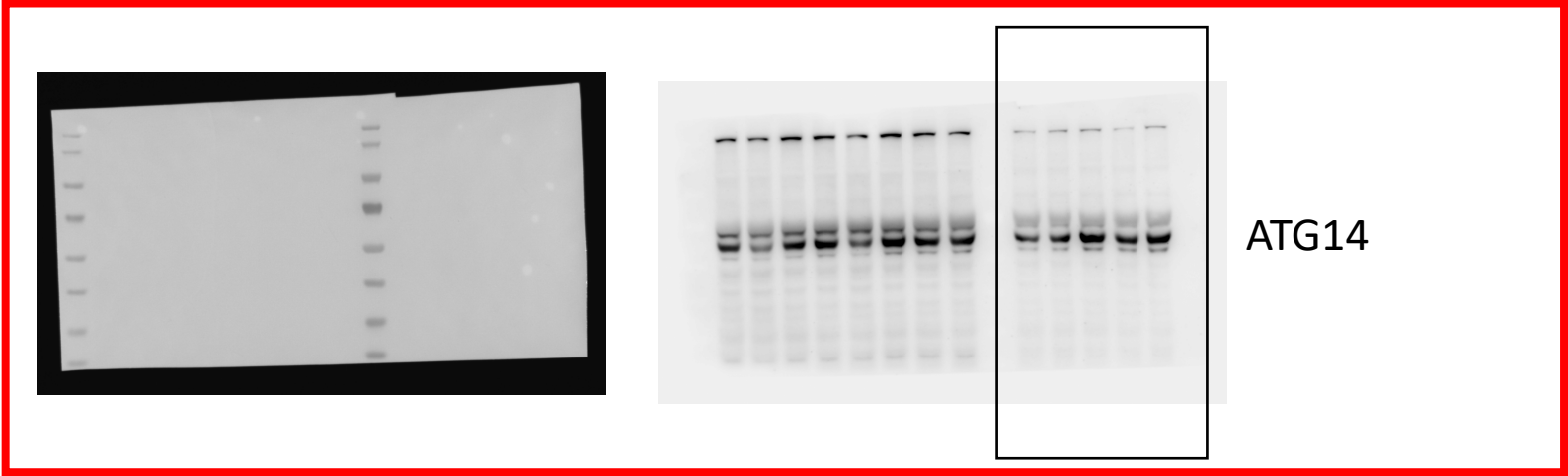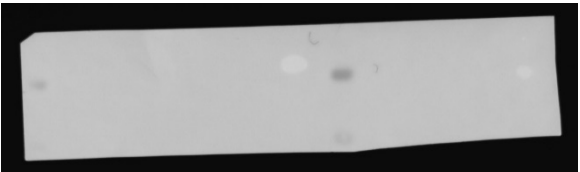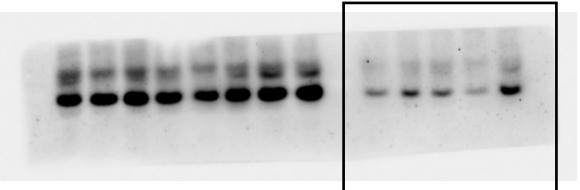

LC3

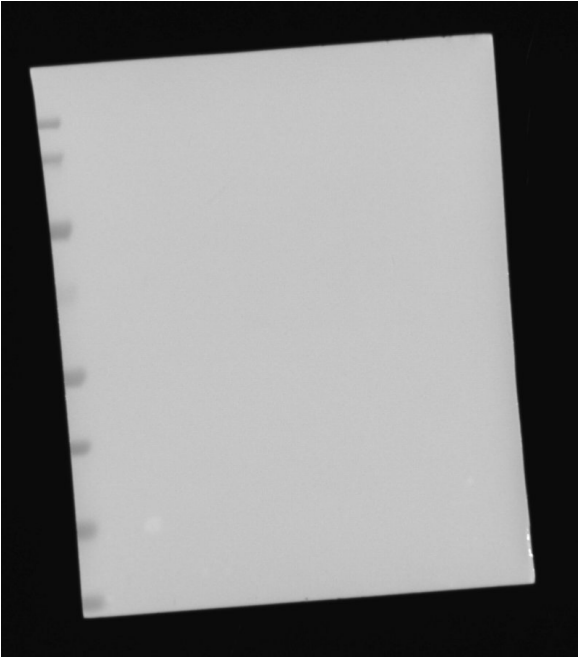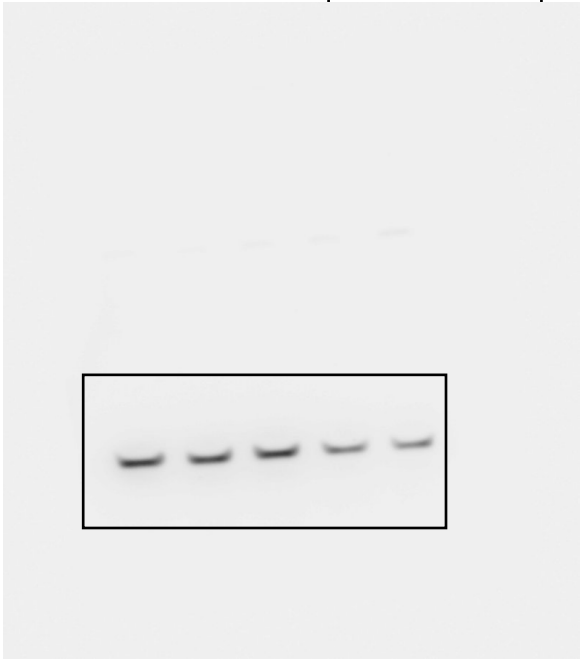

GAPDH

Figure 3B

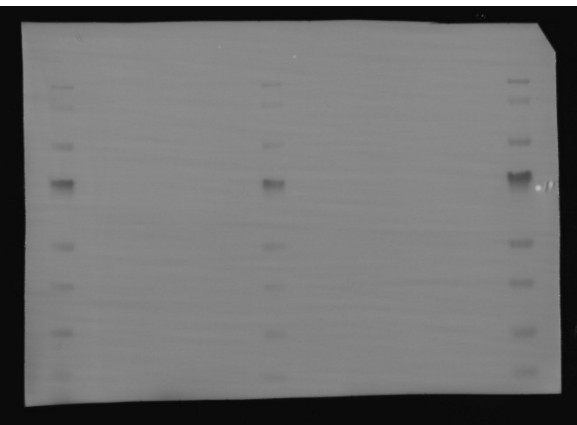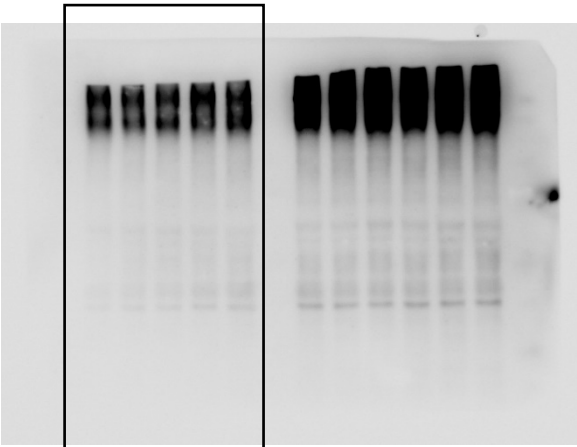

Lamp1

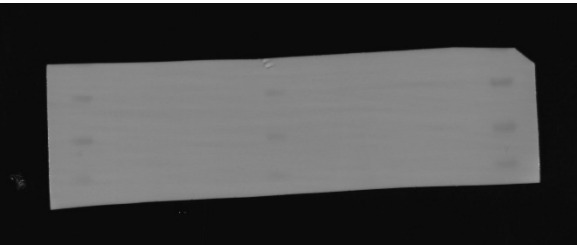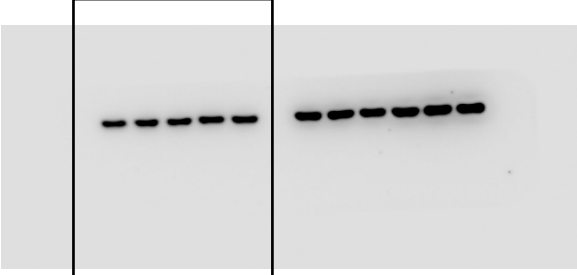

GAPDH

Supplement: Supplementary file 6 — Source Data for Figure 3 [file EMMM-11-e10469-s004.pdf]

Figure 8A

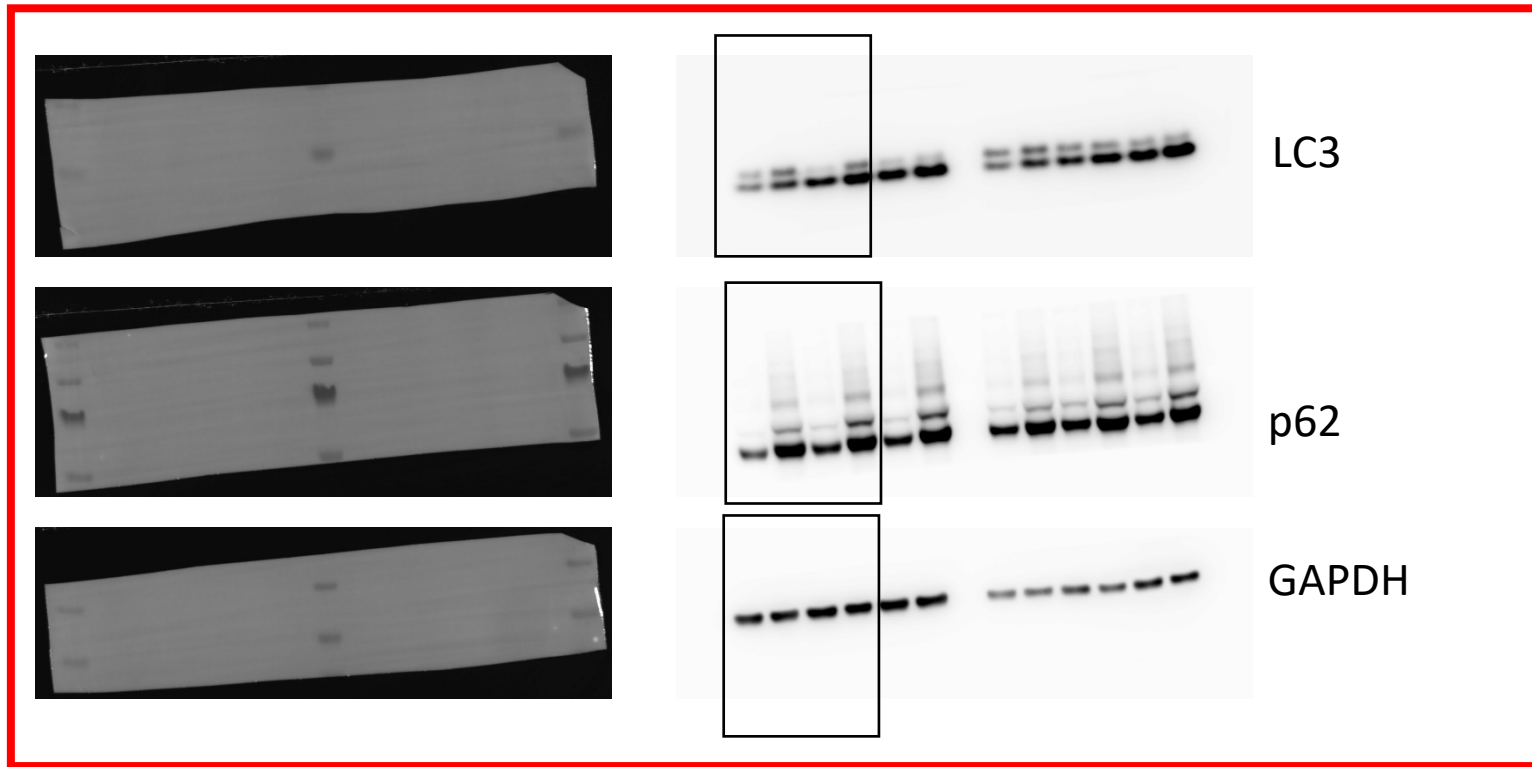

Figure 8H

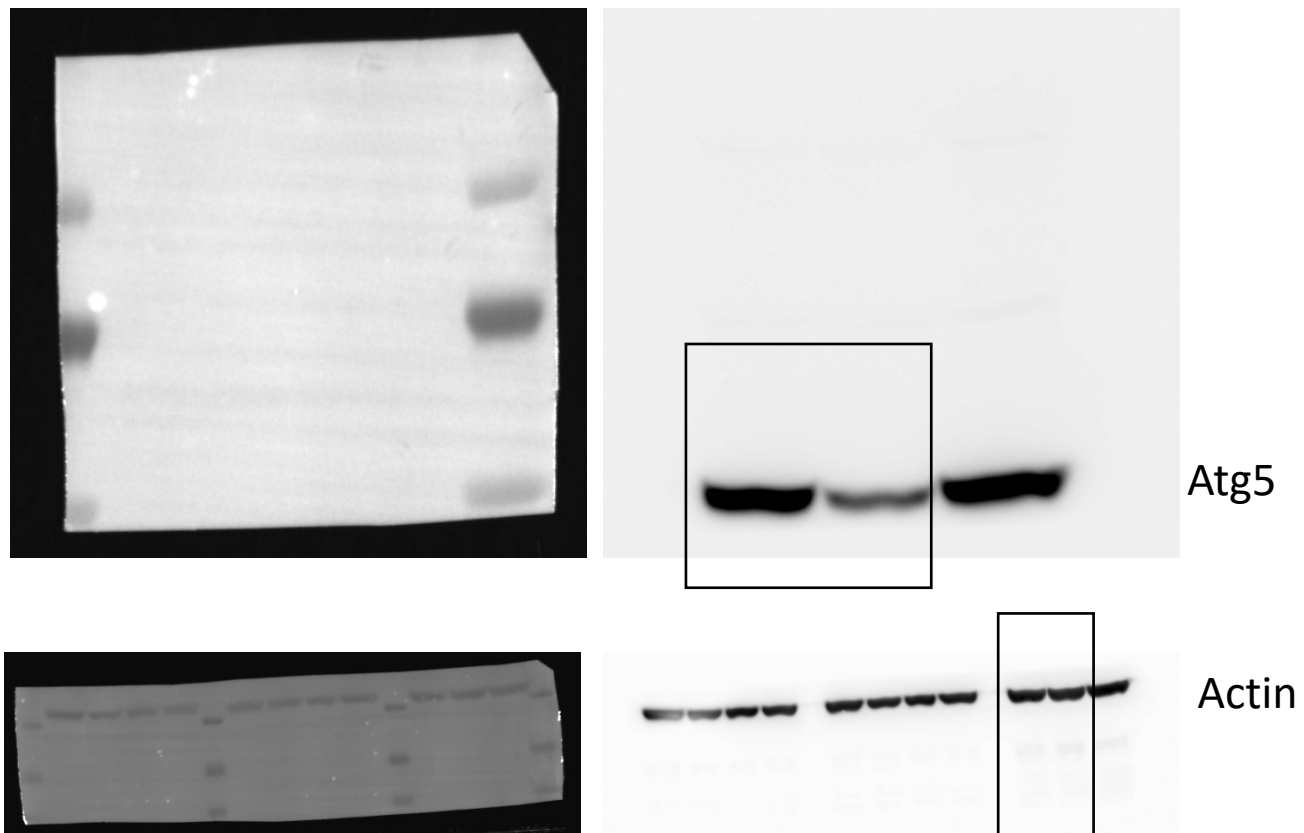

Figure 8K

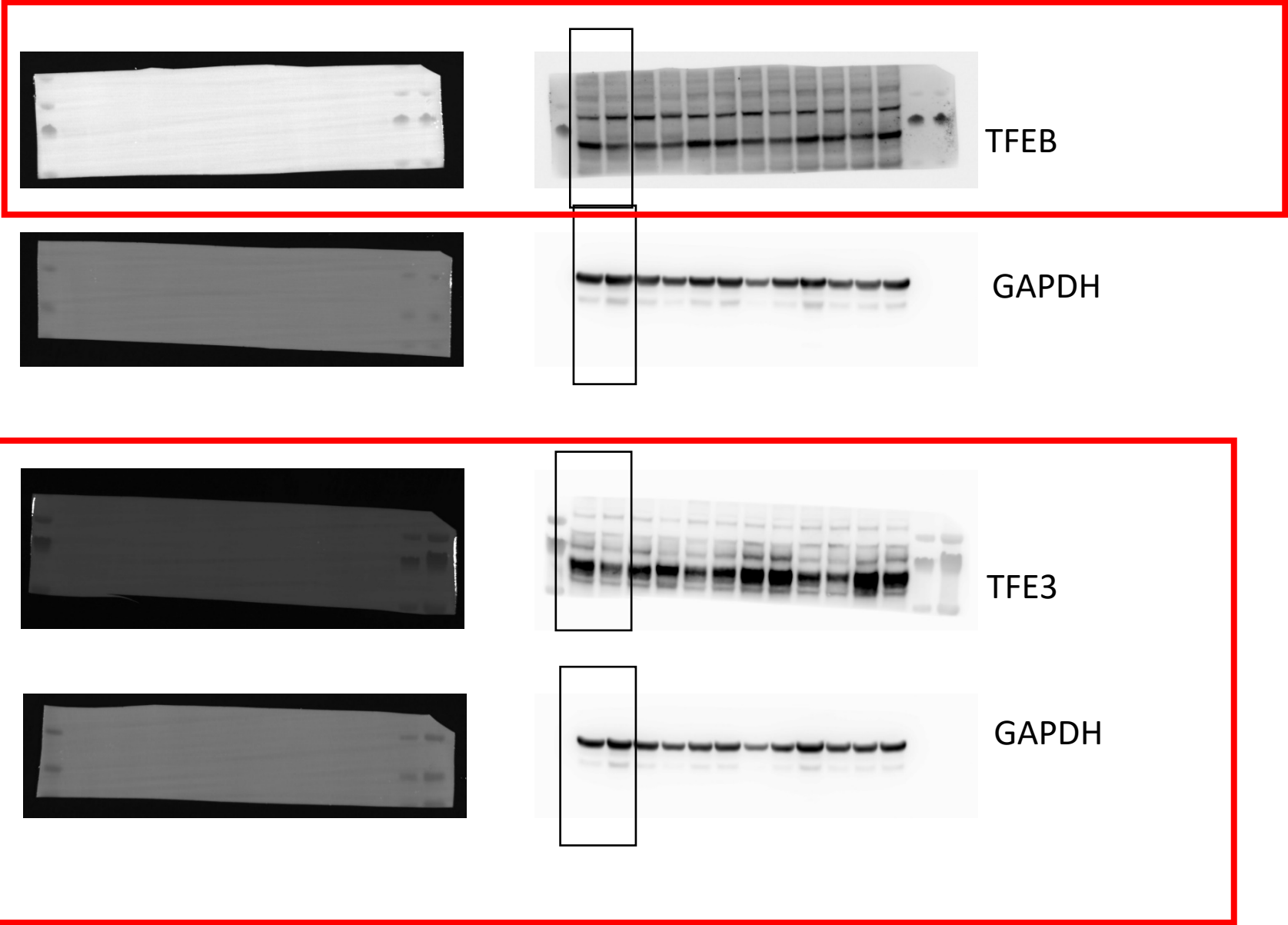

Supplement: Supplementary file 10 — Source Data for Figure 8 [file EMMM-11-e10469-s008.pdf]
