# Supplementary material for: 3,4‐Dimethoxychalcone induces autophagy through activation of the transcription factors TFE3 and TFEB
Source: EMBO Mol Med. 2019 Oct 14;11(11):e10469. doi: 10.15252/emmm.201910469 (PMC6835206; doi:10.15252/emmm.201910469)

Figure 2A

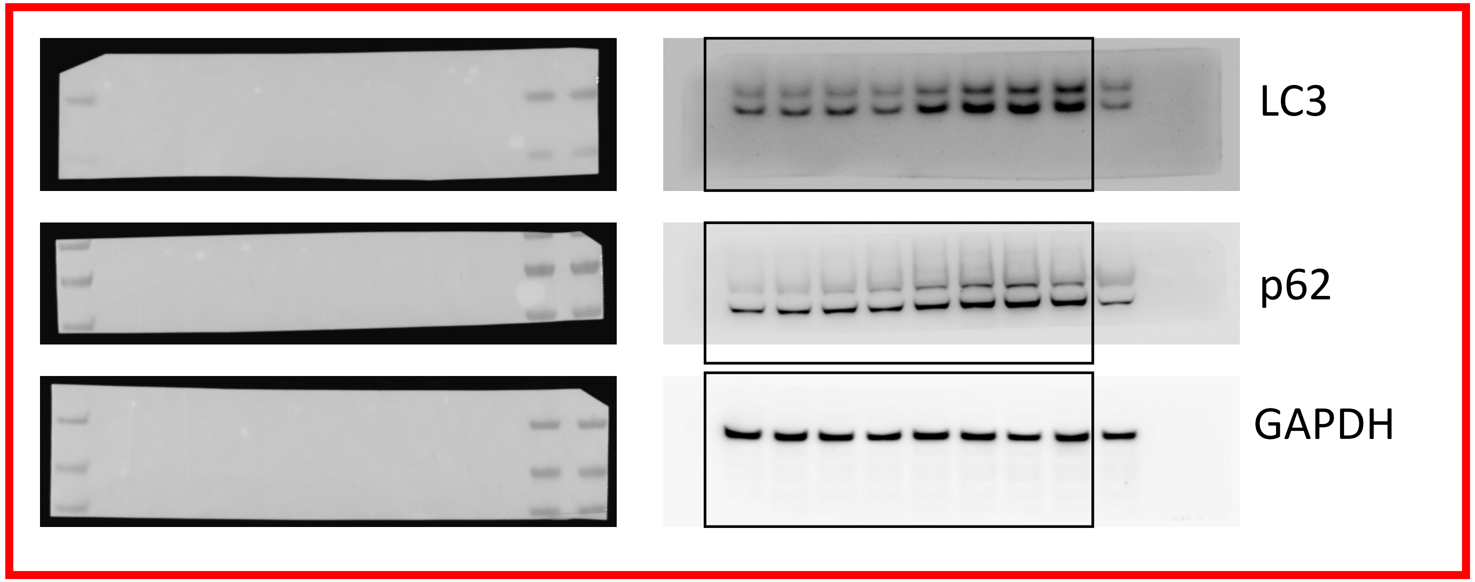

Figure 2C

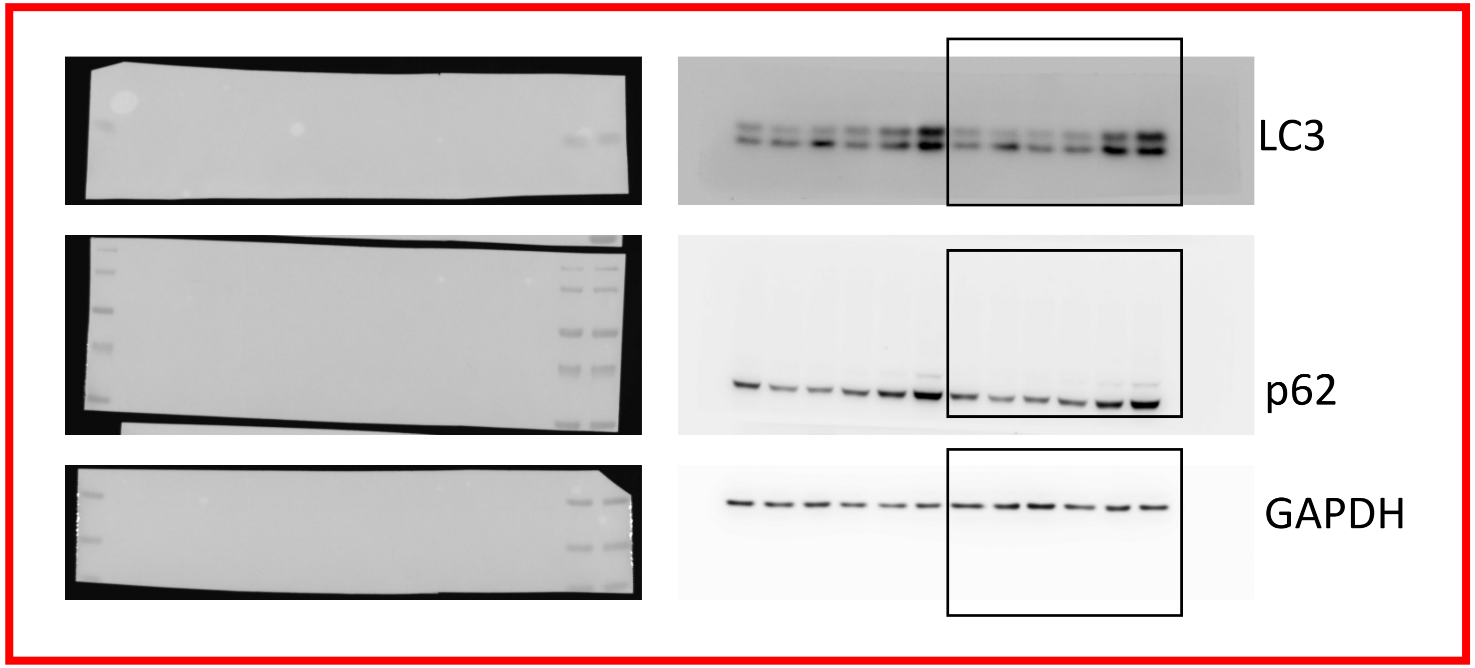

Figure 2E

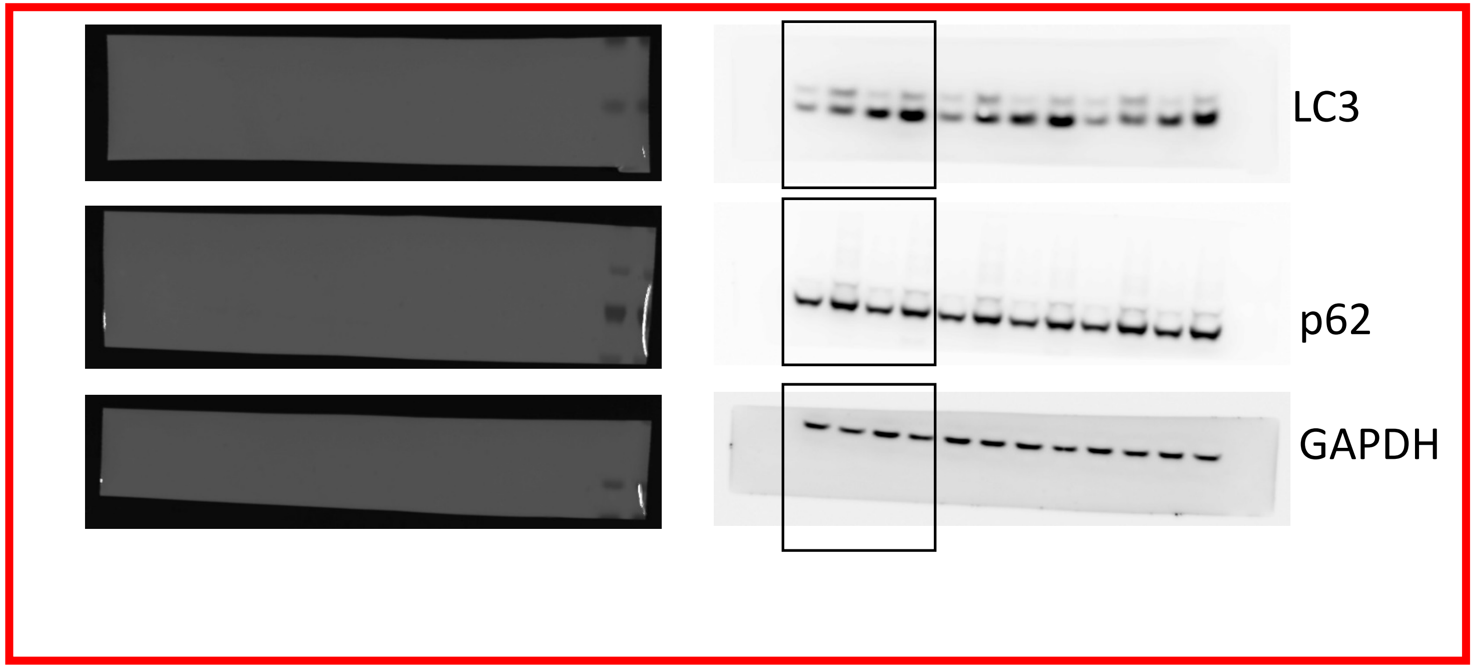

Supplement: Supplementary file 5 — Source Data for Figure 2 [file EMMM-11-e10469-s003.pdf]
