# Supplementary material for: 3,4‐Dimethoxychalcone induces autophagy through activation of the transcription factors TFE3 and TFEB
Source: EMBO Mol Med. 2019 Oct 14;11(11):e10469. doi: 10.15252/emmm.201910469 (PMC6835206; doi:10.15252/emmm.201910469)

Figure 4E

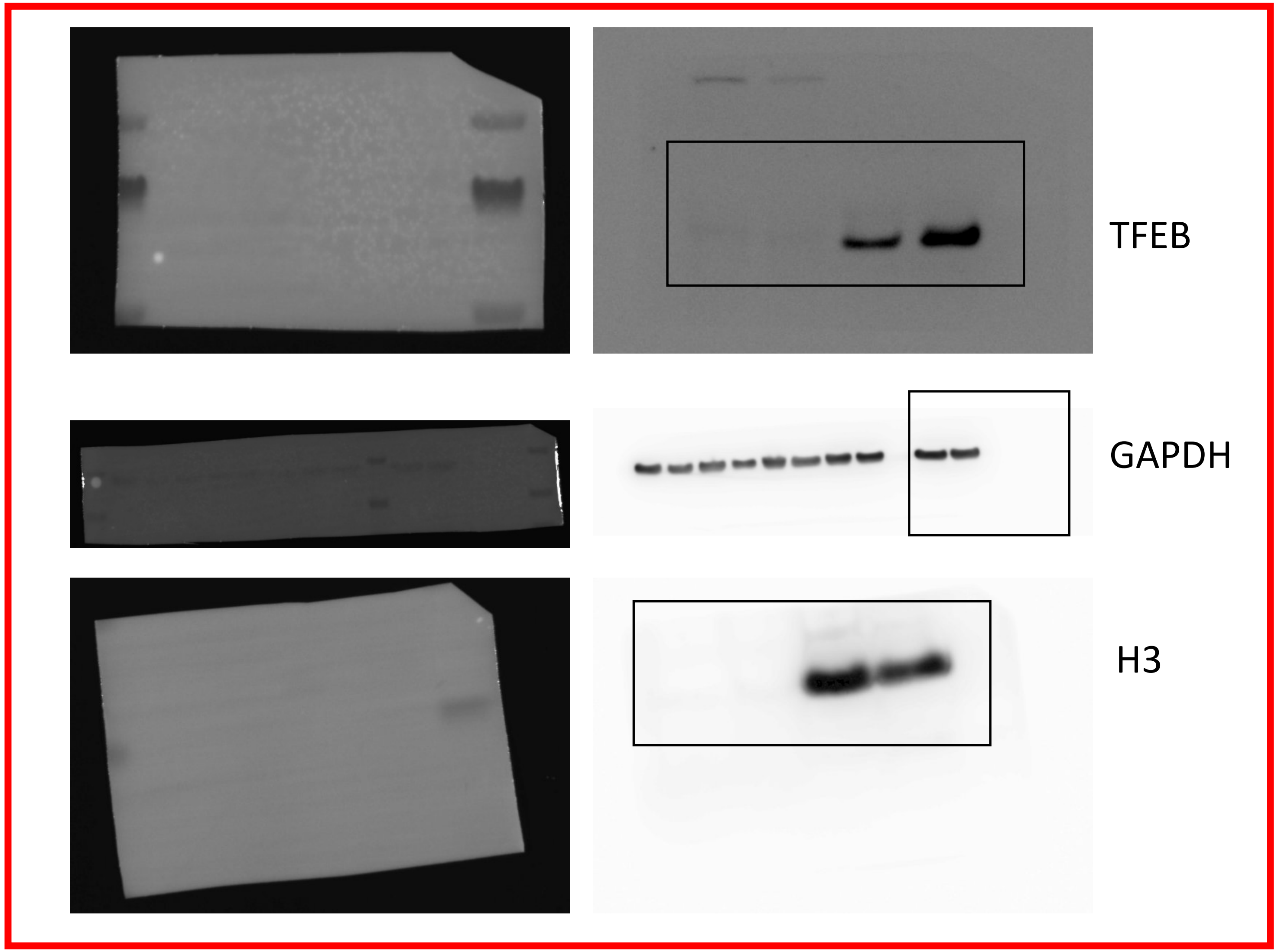

Figure 4F

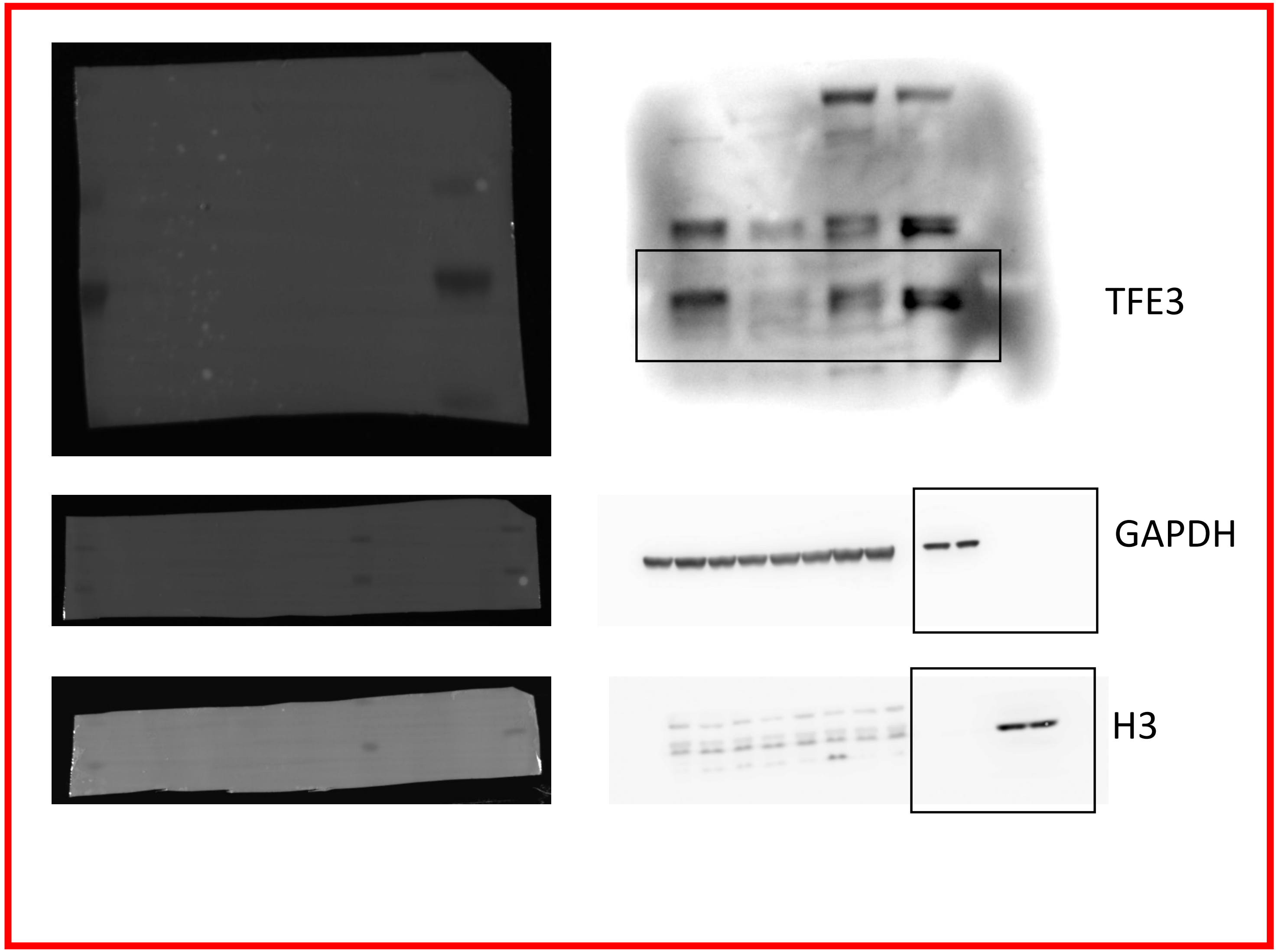

Figure 4l

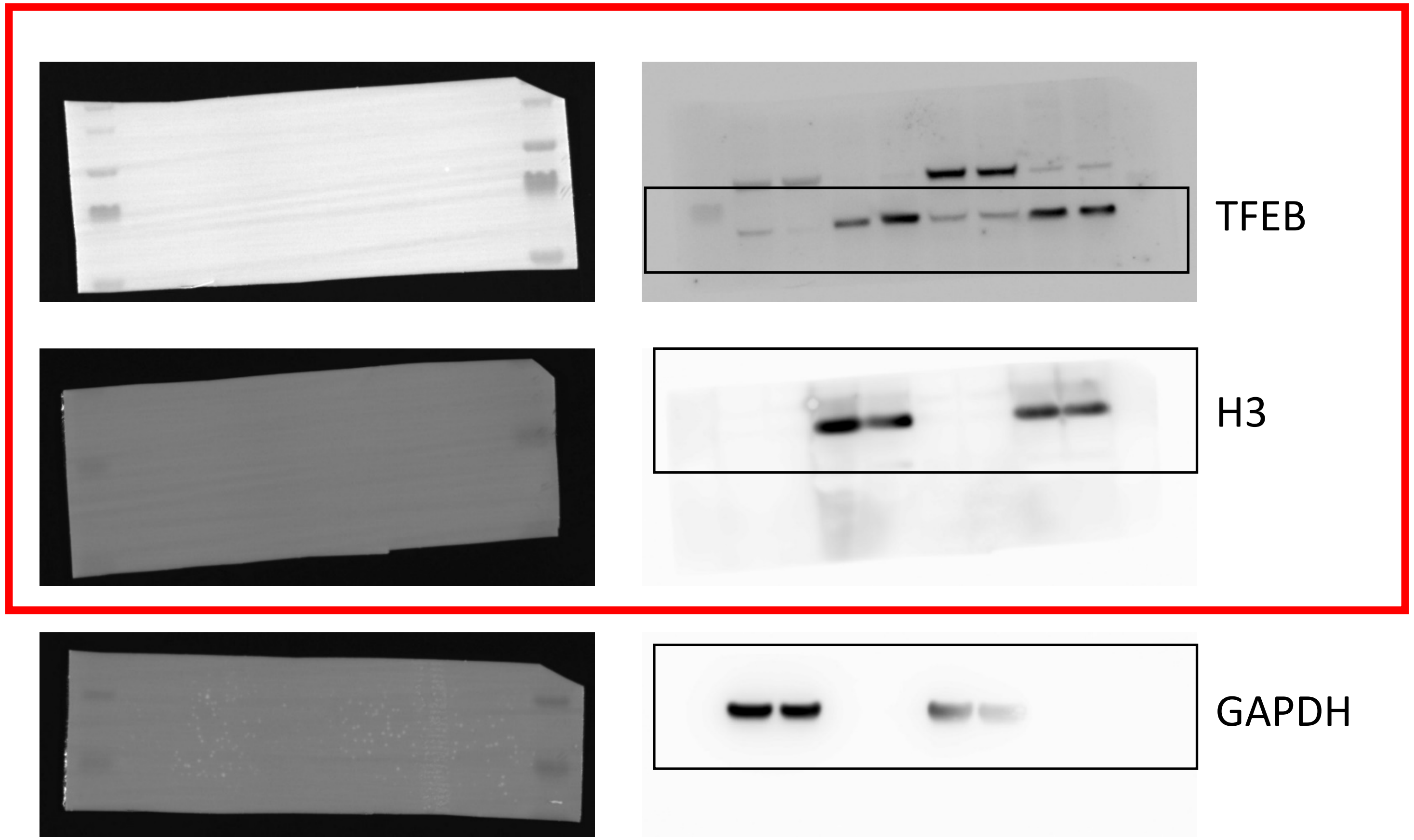

Figure 4I

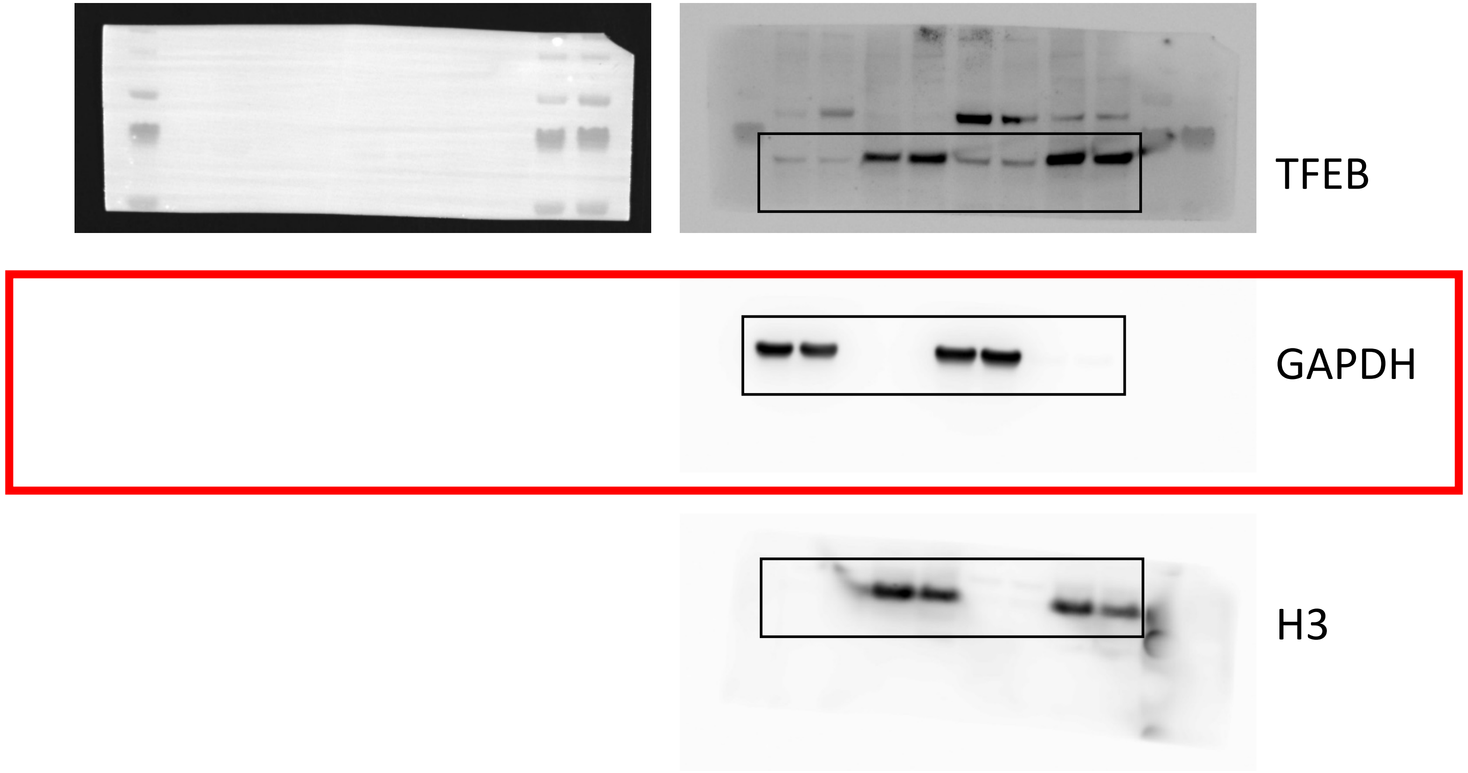

Figure 4J

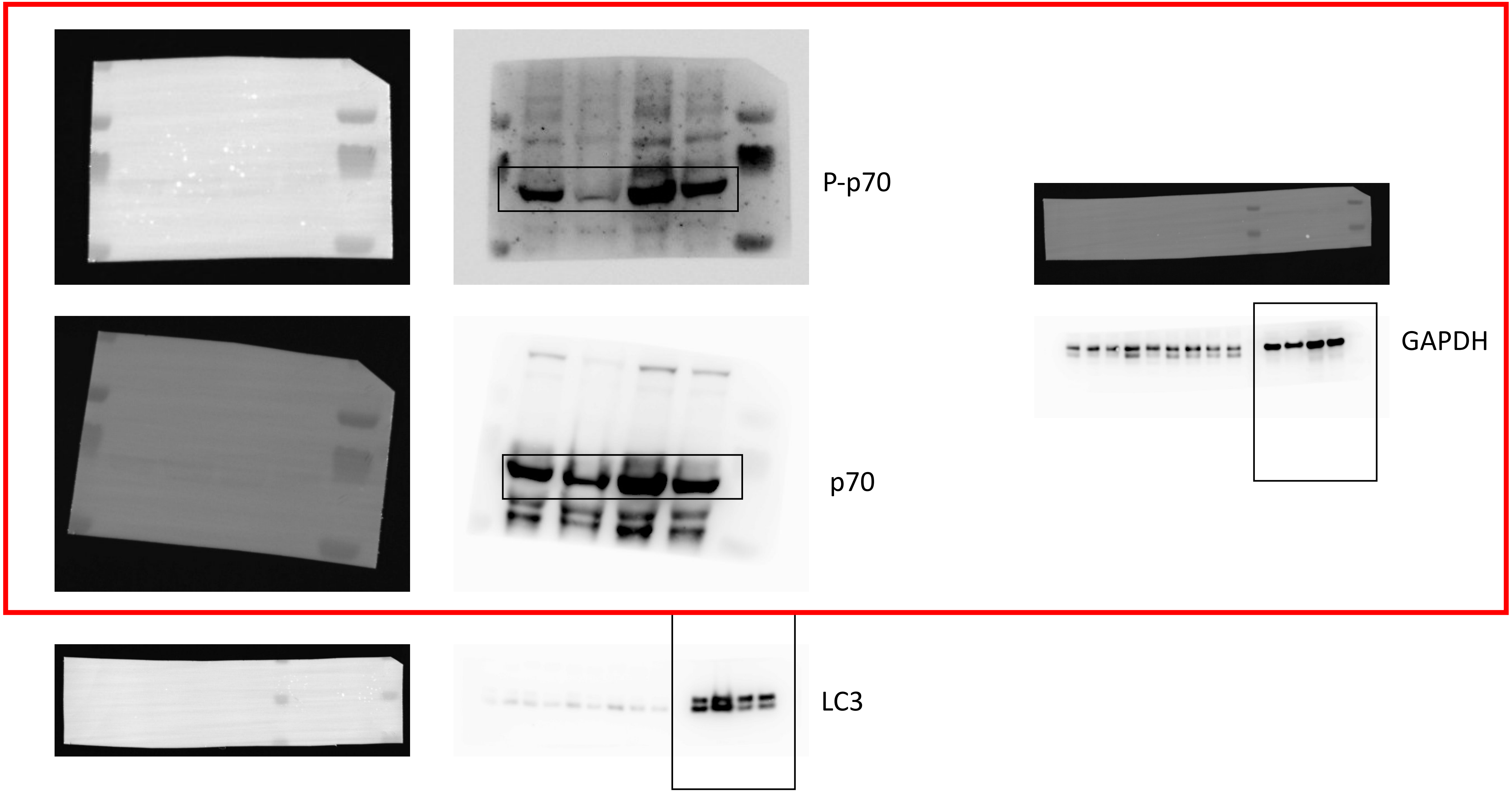

Figure 4J

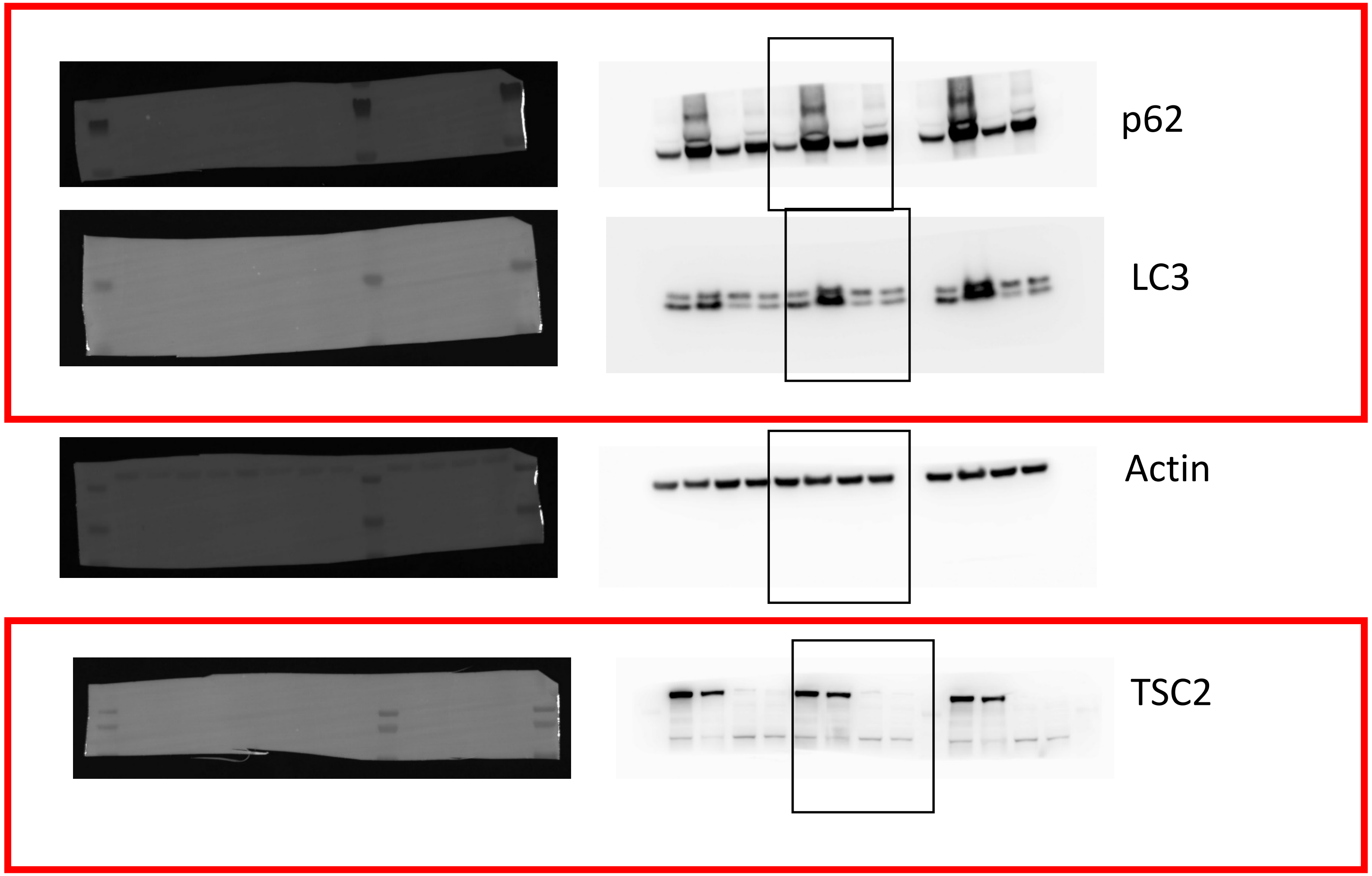

Figure 4M

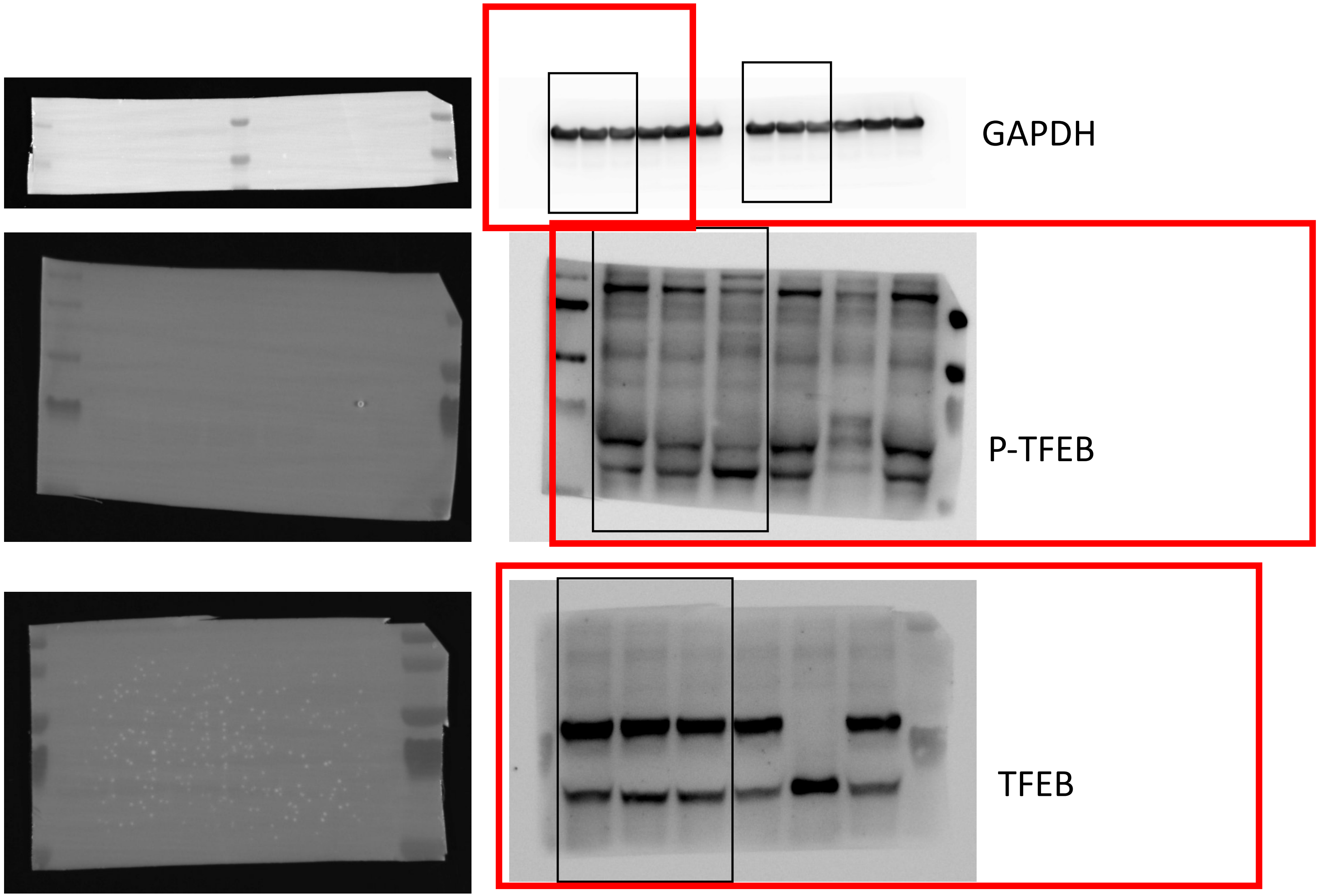

Supplement: Supplementary file 7 — Source Data for Figure 4 [file EMMM-11-e10469-s005.pdf]
