# Supplementary material for: 3,4‐Dimethoxychalcone induces autophagy through activation of the transcription factors TFE3 and TFEB
Source: EMBO Mol Med. 2019 Oct 14;11(11):e10469. doi: 10.15252/emmm.201910469 (PMC6835206; doi:10.15252/emmm.201910469)

Figure 5A

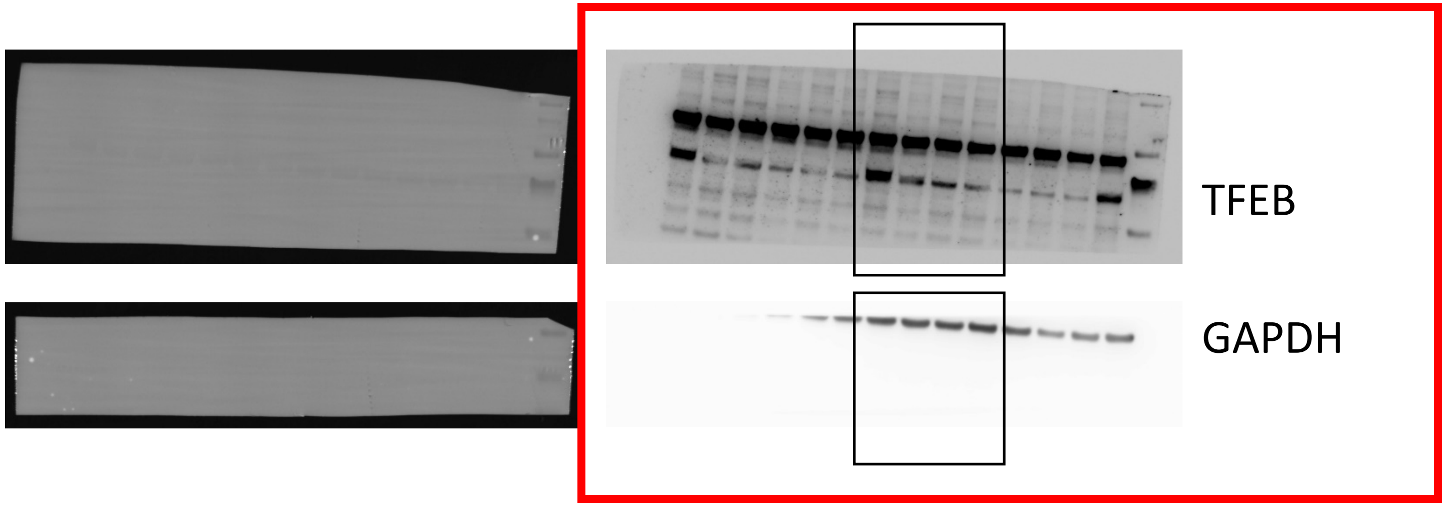

Figure 5D

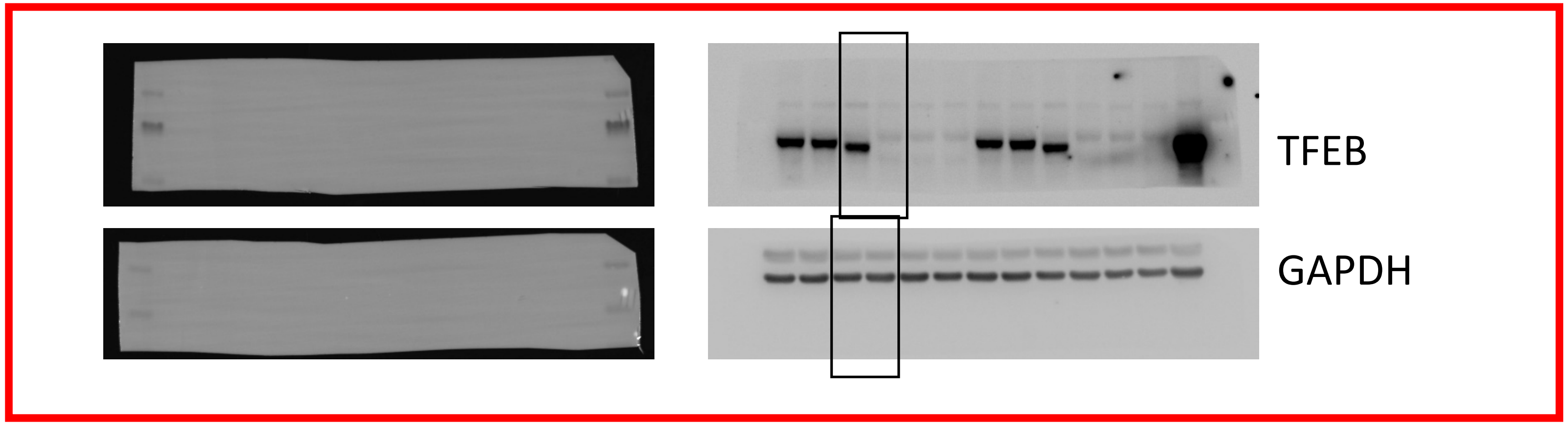

Figure 5G

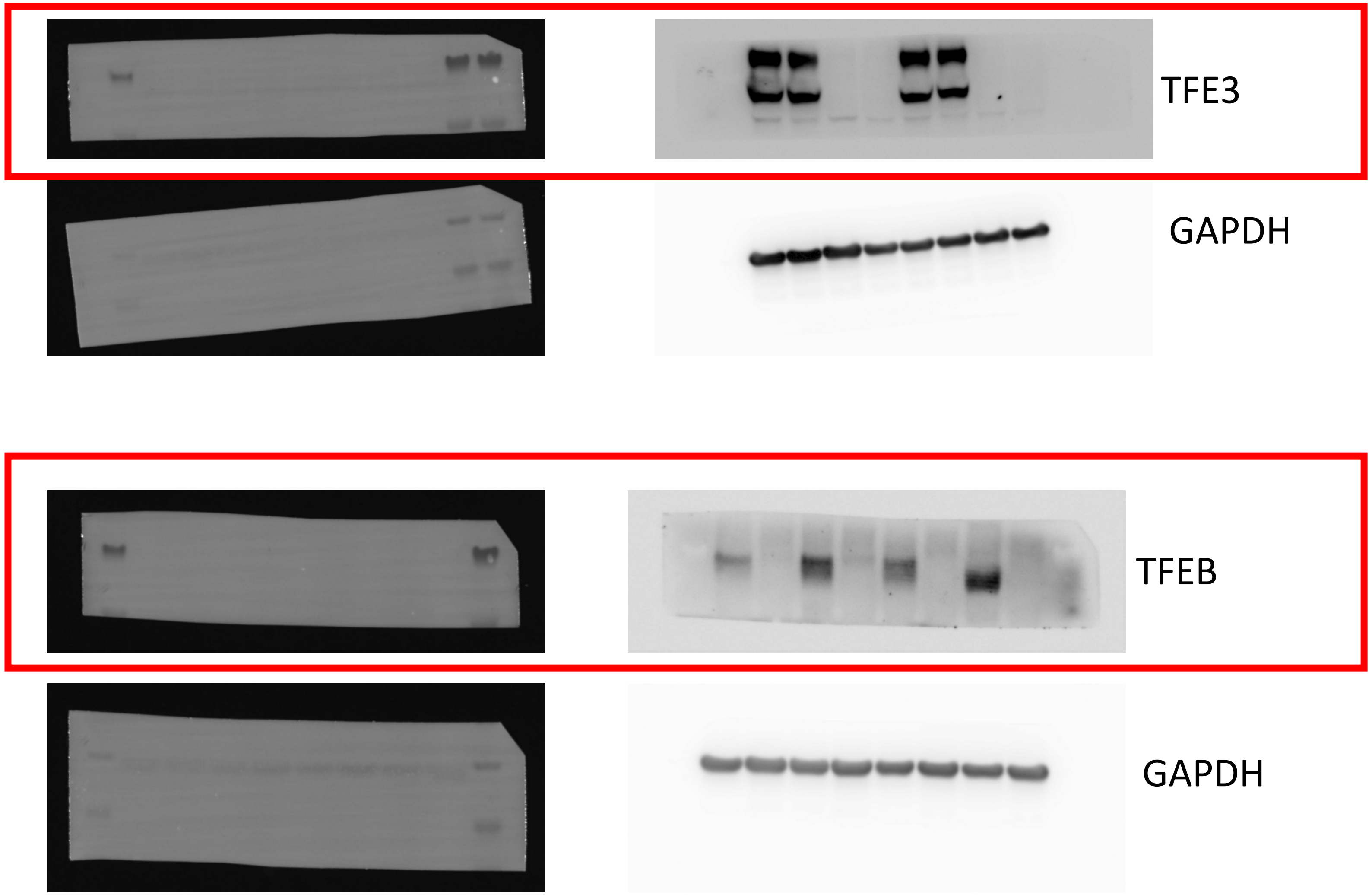

Figure 5G

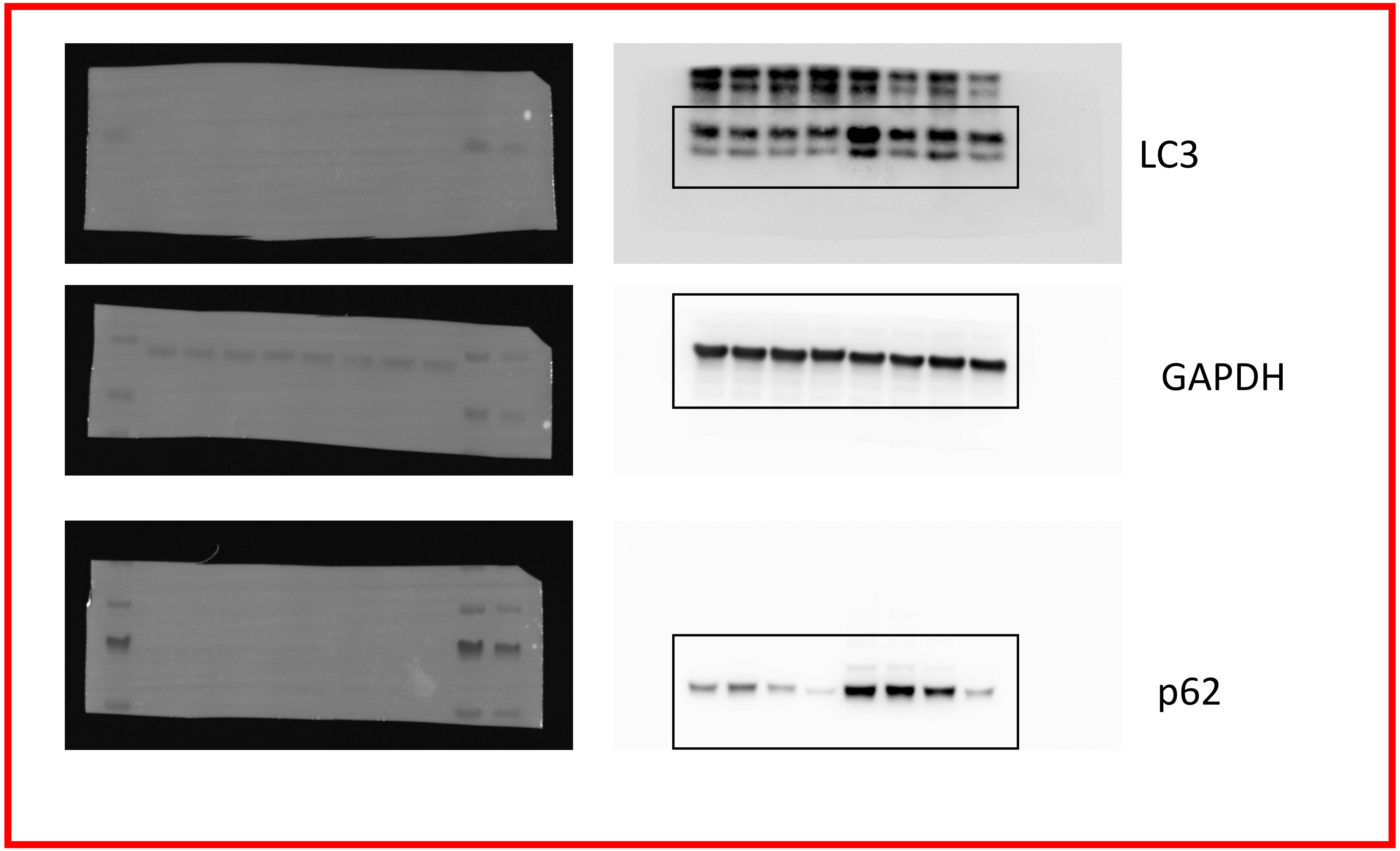

Supplement: Supplementary file 8 — Source Data for Figure 5 [file EMMM-11-e10469-s006.pdf]
